# Larson–Penston Self-similar Gravitational Collapse

**DOI:** 10.1007/s00220-021-04175-y

**Published:** 2021-08-02

**Authors:** Yan Guo, Mahir Hadžić, Juhi Jang

**Affiliations:** 1 Division of Applied Mathematics, Brown University, Providence, RI 02912 USA; 2 Department of Mathematics, University College London, London, WC1E 6XA UK; 3 Department of Mathematics, University of Southern California, Los Angeles, CA 90089 USA; 4 Korea Institute for Advanced Study, Seoul, Korea

## Abstract

Using numerical integration, in 1969 Penston (Mon Not R Astr Soc 144:425–448, 1969) and Larson (Mon Not R Astr Soc 145:271–295, 1969) independently discovered a self-similar solution describing the collapse of a self-gravitating asymptotically flat fluid with the isothermal equation of state $p=k\varrho$, $k>0$, and subject to Newtonian gravity. We rigorously prove the existence of such a Larson–Penston solution.

## Isothermal Euler–Poisson system

The classical model of a self-gravitating Newtonian star is given by the gravitational Euler–Poisson system. We work in three spatial dimensions and assume radial symmetry. The unknowns are the gas density $\varrho (t,r)$, the pressure *p*(*t*, *r*), and the radial velocity *u*(*t*, *r*), where *t* is the time coordinate and $r=|x|$ the radial coordinate in ${\mathbb {R}}^3$. The equations take the form

1.1 $$\begin{aligned} \partial _t \varrho + \left( \partial _r+\frac{2}{r}\right) (\varrho u)&= 0, \end{aligned}$$

1.2 $$\begin{aligned} \varrho \left( \partial _t u + u\partial _r u\right) + \partial _r p + \varrho \frac{m(t,r)}{r^2}&= 0, \end{aligned}$$

1.3 $$\begin{aligned} m(t,r)&= \int _0^r 4\pi \sigma ^2 \varrho (t,\sigma )\,d\sigma . \end{aligned}$$

Equation (1.1) is the continuity equation, Eq. (1.2) expresses the conservation of momentum, while the term $\frac{m(t,r)}{r^2}$ is the radial component of the gravitational force induced by an asymptotically flat gravitational potential $\phi$ solving the Poisson equation $\Delta \phi = 4\pi \varrho$. To complete the formulation of the problem we impose the *isothermal* equation of state, i.e. we let

1.4 $$\begin{aligned} p = k \varrho , \ \ k>0. \end{aligned}$$

Here $\sqrt{k}$ is the speed of sound and it is constant throughout the star. We are interested in the existence of self-similar solutions to (1.1)–(1.4) describing finite time gravitational collapse. The only invariant scaling for (1.1)–(1.4) is given through the transformation

1.5 $$\begin{aligned} \varrho \mapsto \lambda ^{-2}\varrho (\frac{t}{\lambda }, \frac{r}{\lambda }), \ \ u \mapsto u (\frac{t}{\lambda }, \frac{r}{\lambda }). \end{aligned}$$

Motivated by this invariance, we seek a self-similar solution of (1.1)–(1.4) of the form:

1.6 $$\begin{aligned} \varrho (t,r)&= (\sqrt{2\pi } \, t)^{-2}{{\tilde{\rho }}}(y), \ \ u(t,r) = \sqrt{k}{{\tilde{u}}}(y), \ \ \end{aligned}$$

where

1.7 $$\begin{aligned} y : = \frac{r}{-\sqrt{k} t}. \end{aligned}$$

It is convenient to introduce the relative velocity

1.8 $$\begin{aligned} {{\tilde{\omega }}} : = \frac{{{\tilde{u}}}(y)+y}{y}. \end{aligned}$$

Applying the above change of variables, the Euler–Poisson system (1.1)–(1.4) becomes

1.9 $$\begin{aligned} {{\tilde{\rho }}}'&= -\frac{2 y {{\tilde{\omega }}}{{\tilde{\rho }}}}{1- y^2{{\tilde{\omega }}}^2}({{\tilde{\rho }}}-{{\tilde{\omega }}}) \end{aligned}$$

1.10 $$\begin{aligned} {{\tilde{\omega }}}'&= \frac{1-3{{\tilde{\omega }}}}{y} + \frac{2y{{\tilde{\omega }}}^2}{1-y^2{{\tilde{\omega }}}^2}({{\tilde{\rho }}}-{{\tilde{\omega }}}), \end{aligned}$$

where the derivative notation $'$ is short for $\partial _y$. A simple Taylor expansion at the origin $y=0$ and the asymptotic infinity $y\rightarrow +\infty$ shows that in order for a solution $({{\tilde{\rho }}},{{\tilde{\omega }}})$ to (1.9)–(1.10) to be smooth and asymptotically flat, we must have

1.11 $$\begin{aligned} {{\tilde{\omega }}}(0)&= \frac{1}{3}, \ \ {{\tilde{\rho }}}(0)>0 \end{aligned}$$

1.12 $$\begin{aligned} {{\tilde{\rho }}}(y)&\sim _{y\rightarrow \infty } y^{-2}, \ \ \lim _{y\rightarrow \infty }{{\tilde{\omega }}}(y)=1. \end{aligned}$$

By continuity, for any continuous solution satisfying (1.11)–(1.12) there must exist at least one point $y_*$ such that $1-y_*^2{{\tilde{\omega }}}^2(y_*)=0$. At such a point the system (1.9)–(1.10) is in general singular. This leads us to one of the central notions in this paper.

### Definition 1.1

(*Sonic point*). A point $y_*>0$ is called a *sonic point* for the flow $({{\tilde{\rho }}}(\cdot ),{{\tilde{\omega }}}(\cdot ))$ if

1.13 $$\begin{aligned} 1-y_*^2{{\tilde{\omega }}}^2(y_*)=0. \end{aligned}$$

For a solution to be smooth through the sonic point $y_*$, it has to be the case that the sonic point is a removable singularity. Assuming smoothness, we can formally compute the Taylor coefficients of $({{\tilde{\rho }}},{{\tilde{\omega }}})$ around $y_*$. Two possibilities emerge (see e.g. [2])—either

1.14 $$\begin{aligned} {{\tilde{\rho }}}(y)&= \frac{1}{y_*} -\frac{1}{y_*^2} (y-y_*) + \frac{-y_*^2+6y_*-7}{2y_*^3(2y_*-3)} (y-y_*)^2 +O(|y-y_*|^3) \end{aligned}$$

1.15 $$\begin{aligned} {{\tilde{\omega }}}(y)&= \frac{1}{y_*} + \frac{1}{y_*}(1-\frac{2}{y_*}) (y-y_*) + \frac{-5y_*^2+19y_*-17}{2y_*^3(2y_*-3)}(y-y_*)^2 + O(|y-y_*|^3), \end{aligned}$$

or

1.16 $$\begin{aligned} {{\tilde{\rho }}}(y)&= \frac{1}{y_*} +\frac{1}{y_*}\left( 1-\frac{3}{y_*}\right) (y-y_*) +O((y-y_*)^2), \end{aligned}$$

1.17 $$\begin{aligned} {{\tilde{\omega }}}(y)&= \frac{1}{y_*} + O((y-y_*)^2). \end{aligned}$$

Using numerics, in 1969 in their seminal works Penston [22] and Larson [17] independently discovered an asymptotically flat smooth solution to (1.9)–(1.10) which satisfies the boundary conditions (1.11)–(1.12). Their solution passes through a single sonic point $y_*$ and conforms to the expansion of the type (1.14)–(1.15). In the literature, this solution is commonly referred to as the Larson–Penston (LP) collapsing solution. There have been numerous studies of self-similar collapse for isothermal stars in the astrophysics literature and here we only provide a brief overview. In 1977 Hunter [14] numerically discovered a further (discrete) family of smooth self-similar solutions, commonly referred to as Hunter solutions, see also the important work of Shu [24]. The Taylor expansion of the Hunter solutions around the sonic point is of the form (1.16)–(1.17). A thorough analysis of the various types of self-similar solutions is given by Whitworth and Summers [25]. In 1988 Ori and Piran [23] gave numerical evidence that the LP collapse is the only stable self-similar solution in the above family of solutions, and therefore physically the most relevant. Brenner and Witelski [2], Maeda and Harada [18] reached the same conclusion performing careful numerical analysis of the collapse. The LP-solutions also play an important role in the study of so-called critical phenomena [11] and are of central importance in astrophysics, see e.g. [12]. The central result of this work is the proof of existence of an LP-solution.

### Theorem 1.2

(Existence of a Larson–Penston self-similar collapsing solution). There exists a $y_*\in (2,3)$ such that (1.9)–(1.12) possesses a real-analytic solution $({{\tilde{\rho }}},{{\tilde{\omega }}})$ with a single sonic point at $y_*$. Moreover the solution satisfies the Larson–Penston expansion (1.14)–(1.15) at $y=y_*$ and

1.18 $$\begin{aligned} {{\tilde{\rho }}}(y)&>0, \ \ y\in [0,\infty ) \end{aligned}$$

1.19 $$\begin{aligned} -\frac{2}{3} y\le {{\tilde{u}}}(y)&<0, \ \ y\in [0,\infty ), \end{aligned}$$

where we recall (1.8).

### Remark 1.3

There are two known explicit solutions to (1.9)–(1.12). One of them is the Friedman solution

1.20 $$\begin{aligned} {{\tilde{\rho }}}_F(y) = {{\tilde{\omega }}}_F(y) \equiv \frac{1}{3} \end{aligned}$$

and the other one is the far-field solution

1.21 $$\begin{aligned} {{\tilde{\rho }}}_\infty (y) = \frac{1}{y^2}, \ \ {{\tilde{\omega }}}_\infty (y) \equiv 1. \end{aligned}$$

The Friedman solution (1.20) is the Newtonian analogue of the classical cosmological Friedman solution—it satisfies the boundary condition (1.11), but is not asymptotically flat. On the other hand, the far-field solution (1.21) is asymptotically flat, but blows up at the origin $y=0$.

If the linear equation of state (1.4) is replaced by the polytropic law $p=\varrho ^\gamma$, $\gamma >1$, it is well known that there cannot exist any collapsing solutions with finite mass and energy in the regime $\gamma >\frac{4}{3}$, see [4]. When $\gamma =\frac{4}{3}$ there exists a special class of self-similar collapsing and expanding solutions [4, 5, 7, 19]. The nonlinear stability in the expanding case was shown in [13]. When $1<\gamma <\frac{4}{3}$ the authors in [9] showed the existence of an infinite-dimensional class of collapsing solutions to the gravitational Euler–Poisson system. When one considers the Euler–Poisson system with an electric (instead of gravitational) force field, the dispersive nature of the problem becomes dominant. A lot of progress has been made in recent decades, we refer the reader to [6, 8, 10, 15] and references therein.

To prove Theorem 1.2, it is natural to consider the following change of variables

1.22 $$\begin{aligned} z = \frac{y}{y_*}, \ \ \ {{\tilde{\omega }}}(y) = \omega (z), \ \ \ {{\tilde{\rho }}}(y) = \rho (z). \end{aligned}$$

The unknown sonic point $y_*$ is mapped to $z=1$. The system (1.9)–(1.10) takes the form

1.23 $$\begin{aligned} \rho '&= -\frac{2y_*^2 z \omega \rho }{1-y_*^2 z^2\omega ^2}(\rho -\omega ) \end{aligned}$$

1.24 $$\begin{aligned} \omega '&= \frac{1-3\omega }{z} + \frac{2y_*^2z\omega ^2}{1-y_*^2z^2\omega ^2}(\rho -\omega ). \end{aligned}$$

We shall work with this formulation for the rest of the paper and often, by abuse of terminology, refer to the point $z=1$ as the sonic point. It is now obvious from the LP sonic point expansion (1.14)–(1.15) that

1.25 $$\begin{aligned} \omega (1) = \rho (1)= \frac{1}{y_*}, \end{aligned}$$

for any solution satisfying $\omega (1),\rho (1)>0$. If we define the infinitesimal increment

1.26 $$\begin{aligned} \delta z: = z-1, \end{aligned}$$

we formally assume that locally around the sonic point

1.27 $$\begin{aligned} \rho&= \sum _{N=0}^\infty \rho _N (\delta z)^N, \ \ \omega = \sum _{N=0}^\infty \omega _N (\delta z)^N \end{aligned}$$

In this notation $\omega _0=\rho _0 = \frac{1}{y_*}$ and (1.14)–(1.15) gives us

1.28 $$\begin{aligned} (\rho _1,\omega _1) = (-\frac{1}{y_*}, 1-\frac{2}{y_*}), \ \ (\rho _2,\omega _2) = \left( \frac{-y_*^2+6y_*-7}{2y_*(2y_*-3)}, \frac{-5y_*^2+19y_*-17}{2y_*(2y_*-3)}\right) \end{aligned}$$

For any $y_*>0$ we shall say that a solution of (1.23)–(1.24) is of *Larson-Penston (LP)-type* if the conditions (1.25) and (1.28) hold. We shall prove in Theorem 2.10 that for any $y_*>\frac{3}{2}$ the LP-type conditions (1.25) and (1.28) uniquely specify a real analytic solution in some small neighbourhood of $z=1$. We denote this flow by $(\rho (\cdot ;y_*),\omega (\cdot ;y_*))$.

### Methodology

The sonic point in the original (*t*, *r*)-variables corresponds to the backward cone emanating from the singularity (0, 0) and it takes the form $\frac{r}{-t} = -u(t,r)\pm \sqrt{k}$, $t<0$. More details on the geometric meaning of the sonic point in this context can be found for example in [2]. Sonic points appear naturally in self-similar formulation of equations of fluid mechanics (see [1, 3, 12, 16, 20] and references therein). They present a fundamental difficulty in our proof of Theorem 1.2, as we cannot use any standard ODE theory to construct a real analytic (or a $C^\infty$) solution. This is well illustrated in a recent pioneering study of sonic points for the compressible Euler system [20], where the authors use the equation of state $p=\varrho ^\gamma$, $\gamma >1$. Using delicate arguments the authors [20] systematically develop the existence theory for $C^\infty$ self-similar solutions of the Euler flow and such a smoothness is crucial in the proof of their nonlinear stability [21].

The self-similar problem associated with the Euler–Poisson system leads to an ODE-system which is not autonomous [see (1.23)–(1.24)]. We also emphasise that the presence of gravity fixes exactly one invariant scale in the problem, see (1.5). Our proof uses in essential way dynamic invariances specific to the flow (1.23)–(1.24). The sonic point separates the positive semi-axis $z\ge 0$ into an inner region [0, 1] and an outer region $[1,\infty )$ (i.e. $[0,y_*]$ and $[y_*,\infty )$ in the *y*-variable). The first and the easier step is to construct an LP-type solution in the outer region satisfying the boundary condition (1.12). This can be done for *any* value of $y_*\in [2,3]$. The remaining key step is to find a value of $y_*\in [2,3]$ such that the associated LP-type solution connects $z=1$ with the *singular* point $z=0$ in the inner region and satisfies the boundary condition (1.11). More specifically, our goal is to choose $y_*\in [2,3]$ so that the local LP-type solution extends to the left all the way to $z=0$ and satisfies $\lim _{z\rightarrow 0}\omega (z;y_*)=\frac{1}{3}$. This motivates us to consider

1.29 $$\begin{aligned} Y : = \left\{ y_*\in [2,3]\,\big | \ \exists \, z \ \ \text {such that } \omega (z;{{\tilde{y}}}_*)=\frac{1}{3} \text { for all } {{\tilde{y}}}_*\in [y_*,3]\right\} . \end{aligned}$$

The curve $(\rho ,\omega )\equiv (\frac{1}{3},\frac{1}{3})$ corresponds to the Friedman curve, see Remark 1.3. We will show that the solution curve $\omega (\cdot ;y_*)$ crosses the Friedman curve strictly inside the interior region and stays trapped below it for $y_*$ sufficiently close to 3. The idea is to lower the value of $y_*$ to the infimum of the set *Y*—we set ${\bar{y}}_*:=\inf Y$. The idea is that $\omega (\cdot ;{\bar{y}}_*)$ will achieve the value $\frac{1}{3}$ exactly at $z=0$ and this will lead to an LP-solution.

Using the minimality of ${\bar{y}}_*$ it is indeed possible to show that the solution exists on (0, 1] and satisfies $\liminf _{z\rightarrow } \omega (z;{\bar{y}}_*)\ge \frac{1}{3}$. To prove that $\lim _{z\rightarrow 0}\omega (z;{\bar{y}}_*)=\frac{1}{3}$ we use a contradiction argument in conjunction with a continuity argument. To explain this, it is necessary to consider the solution of the initial value problem (1.23)–(1.24) starting from $z=0$ *to the right* with the initial values

1.30 $$\begin{aligned} \omega (0)=\frac{1}{3}, \ \ \rho (0) = \rho _0>0. \end{aligned}$$

Just like we did in the vicinity of the sonic point, we resort to Taylor expansion around $z=0$ to prove that (Theorem 2.12) the initial condtions (1.30) specify a unique solution to (1.23)–(1.24) locally to the right of $z=0$. We denote this solution by $(\rho _-(\cdot ;\rho _0),\omega _-(\cdot ;\rho _0))$.

#### Definition 1.4

(*Upper and lower solution*). For any $y_*\in [2,3]$ we say that $(\rho (\cdot ;y_*), \omega (\cdot ;y_*))$ is an *upper* (resp. *lower*) solution at $z_0\in (0,1)$ if there exists $\rho _0>0$ such that

$$\begin{aligned} \rho (z_0;y_*) = \rho _-(z_0;\rho _0) \end{aligned}$$

and

$$\begin{aligned} \omega (z_0;y_*)> \ (\text {resp. } <) \ \omega _-(z_0;\rho _0). \end{aligned}$$

By way of contradiction we assume $\lim _{z\rightarrow 0}\omega (z;{\bar{y}}_*)>\frac{1}{3}$. The strategy is then the following.

- *Step 1: Upper solution.* By analysing distinct dynamic properties of the solution coming from the right $(\rho (\cdot ;{\bar{y}}_*),\omega (\cdot ;{\bar{y}}_*))$ and the $(\rho _-(\cdot ),\omega _-(\cdot ))$ emanating from the left in the region $0<z\ll 1$ we show that there is a choice of $z_0\ll 1$ and $\rho _0=\rho _1>\frac{1}{3}$ such that $(\rho (\cdot ;{\bar{y}}_*),\omega (\cdot ;{\bar{y}}_*))$ is an *upper solution* at $z_0$ in the sense of Definition 1.4.
- *Step 2: Lower solution.* Using the minimality property of $${\bar{y}}_*= \inf Y$$ and dynamic invariances associated with $$(\rho _-(\cdot ),\omega _-(\cdot ))$$ (see Lemma 4.14) it is possible to find $$y_{**}>{\bar{y}}_*$$ such that $$(\rho (\cdot ;y_{**}),\omega (\cdot ;y_{**}))$$ is a *lower solution* at the same $$z_0\ll 1$$ and some $$\rho _0=\rho _2>0$$ in the sense of Definition 1.4. We emphasise that $$z_0$$ is the same in both steps.
- *Step 3: Intersection at* $$z=z_0$$ *and contradiction.* With considerable technical care and the crucial proof of strict monotonicity of the map $$\rho _0\mapsto \rho _-(z;\rho _0)$$ in a region $$0<z_0\ll z\ll 1$$ (see Lemma 4.18), we show that for any $$y_*\in [{\bar{y}}_*, y_{**}]$$ there is a continuous map $$y_*\mapsto \rho _0(y_*)$$ such that
  $$\begin{aligned} \rho (z_0;y_*) = \rho _-(z_0;\rho _0(y_*)), \ \ \rho _0({\bar{y}}_*) = \rho _1, \ \ \rho _0(y_{**}) = \rho _2. \end{aligned}$$
  The Intermediate Value Theorem, Steps 1 and 2 show that there exists $$y_*\in ({\bar{y}}_*,y_{**})\subset Y$$ such that $$(\rho (\cdot ;y_*),\omega (\cdot ;y_*))$$ is a solution to (1.23)–(1.24) such that $$\inf _{z\in (0,1]}\omega (z; y_*)\ge \frac{1}{3}$$, which is a contradiction to the Definition (1.29) of the set *Y*.

Our work provides a general strategy to construct a solution connecting a sonic point and a singular point, such as the origin $z=0$ in this case. The crucial feature of the problem that allows us to find the solution is the contrast between the dynamics of the “right” solution $(\rho (\cdot ;{\bar{y}}_*),\omega (\cdot ;{\bar{y}}_*))$ and the “left" solution $(\rho _-(\cdot ;\rho _0),\omega _-(\cdot ;\rho _0))$ in the region $0<z\ll 1$. This is fundamentally caused by the presence of the singular denominator $\frac{1}{z}$ on the right-hand side of (1.24), which is a generic feature of the 3-dimensionality of the problem and radial symmetry.

**Plan of the paper.** Section 2 is devoted to the proof of the local existence, uniqueness, and regularity theorems for LP-type solutions locally around the sonic point (Theorem 2.10) and around the centre $z=0$ (Theorem 2.12). In Sect. 3 we analyse the solution in the outer region $z>1$. The main statement is Proposition 3.3 which states that for any $y_*\in [2,3]$ there exists a global forward-in-*z* solution to our problem starting from the sonic point $z=1$ (i.e. $y=y_*$). The most difficult part of our work is the analysis of the inner region $z\in [0,1)$ and it is contained in Sect. 4. In Sect. 4.1 we obtain various continuity results for the LP-type flows, including most importantly the upper semi-continuity of the so-called sonic time, see Proposition 4.5. In Section 4.2 we introduce the crucial set *Y* and show that the LP-type flow associated with ${\bar{y}}_*=\inf Y$ starting from $z=1$ exists all the way to $z=0$, see Proposition 4.12. Qualitative properties of the flow $(\rho _-,\omega _-)$ are investigated in Sect. 4.3. Finally, the key intersection argument and the proof that $\lim _{z\rightarrow 0^+}\omega (z;{\bar{y}}_*)=\frac{1}{3}$ is presented in Sect. 4.4, see Propositions 4.22 and 4.23 . Finally, in Sect. 5 we prove the main result—Theorem 1.2.

## Local Well-Posedness Near the Sonic Point and the Origin

### Existence, uniqueness, and regularity near the sonic point

Recalling (1.27), our goal is to compute a recursive relation that expresses the vector $(\rho _N,\omega _N)$ in terms of $\rho _0,\dots ,\rho _{N-1}, \omega _0,\dots , \omega _{N-1}$. For a given function *f* we shall write $(f)_M$ to mean the *M*-th Taylor coefficient in the expansion of *f* around the sonic point $z=1$. In particular,

$$\begin{aligned} (\omega ^2)_M&= \sum _{k+\ell =M} \omega _k\omega _\ell \\ (\omega \rho (\rho -\omega ))_M&= \sum _{k+\ell + m = M}\omega _k\rho _\ell (\rho _m-\omega _m) \\ (\omega ^2(\rho -\omega ))_M&= \sum _{k+\ell + m = M}\omega _k\omega _\ell (\rho _m-\omega _m), \end{aligned}$$

where the summation implicitly runs over all non-negative integers satisfying the indicated constraint.

To compute the Taylor coefficients in (1.27), we first multiply (1.23)–(1.24) by $(1-y_*^2z^2\omega ^2)$

2.31 $$\begin{aligned} \omega ' (1-y_*^2(1+\delta z)^2\omega ^2) - \left( 1-3\omega \right) (1-y_*^2z^2\omega ^2)\frac{1}{1+\delta z} - 2y_*^2(1+\delta z)\omega ^2(\rho -\omega )&=0, \end{aligned}$$

2.32 $$\begin{aligned} \rho ' (1-y_*^2(1+\delta z)^2\omega ^2) +2y_*^2 (1+\delta z) \omega \rho (\rho -\omega )&=0, \end{aligned}$$

where we have written *z* in the form $1+\delta z$.

#### Lemma 2.1

For any $N\ge 0$ the following formulas hold:

2.33 $$\begin{aligned}&(N+1)\rho _{N+1} - y_*^2 \left( \sum _{k+\ell = N}(k+1)\rho _{k+1}(\omega ^2)_\ell + 2\sum _{k+\ell = N-1}(k+1)\rho _{k+1}(\omega ^2)_\ell + \sum _{k+\ell = N-2}(k+1)\rho _{k+1}(\omega ^2)_\ell \right) \nonumber \\&\quad + 2 y_*^2 \left( \left( \omega \rho (\rho -\omega )\right) _N+\left( \omega \rho (\rho -\omega ) \right) _{N-1} \right) = 0 \end{aligned}$$

2.34 $$\begin{aligned}&0 = (N+1)\omega _{N+1} - y_*^2 \left( \sum _{k+\ell = N}(k+1)\omega _{k+1}(\omega ^2)_\ell + 2\sum _{k+\ell = N-1}(k+1)\omega _{k+1}(\omega ^2)_\ell + \sum _{k+\ell = N-2}(k+1)\omega _{k+1}(\omega ^2)_\ell \right) \nonumber \\&\quad - (-1)^N + 3 \sum _{k+m=N}\omega _k(-1)^m + y_*^2 \left( \sum _{\ell +m=N-2}(-1)^m(\omega ^2)_\ell +2\sum _{\ell +m=N-1} (-1)^m(\omega ^2)_\ell +\sum _{\ell +m=N}(-1)^m(\omega ^2)_\ell \right) \nonumber \\&\quad - 3y_*^2 \left( \sum _{k+\ell +m=N-2}(-1)^m\omega _k(\omega ^2)_\ell + 2 \sum _{k+\ell +m=N-1}(-1)^m\omega _k(\omega ^2)_\ell + \sum _{k+\ell +m=N}(-1)^m\omega _k(\omega ^2)_\ell \right) \nonumber \\&\quad - 2y_*^2 \left( \left( \omega ^2(\rho -\omega )\right) _N+\left( \omega ^2(\rho -\omega ) \right) _{N-1} \right) . \end{aligned}$$

#### Proof

We plug in (1.27) into (2.32) and obtain

2.35 $$\begin{aligned} 0&= \left( \sum _{k=0}^\infty k \rho _k (\delta z)^{k-1}\right) \left( 1 - y_*^2\sum _{\ell =0}^\infty (\omega ^2)_\ell \left( (\delta z)^{\ell +2} +2(\delta z)^{\ell +1}+(\delta z)^\ell \right) \right) \nonumber \\&\ \ \ \ + 2 y_*^2 \sum _{k=0}^\infty \left( \omega \rho (\rho -\omega ) \right) _k\left( (\delta z)^{k+1}+(\delta z)^k\right) \nonumber \\&= \sum _{N=0}^\infty (N+1)\rho _{N+1}(\delta z)^N \nonumber \\&\ \ \ \ - y_*^2 \sum _{N=0}^\infty \left( \sum _{k+\ell = N}(k+1)\rho _{k+1}(\omega ^2)_\ell + 2\sum _{k+\ell = N-1}(k+1)\rho _{k+1}(\omega ^2)_\ell \right. \nonumber \\&\quad \left. + \sum _{k+\ell = N-2}(k+1)\rho _{k+1}(\omega ^2)_\ell \right) (\delta z)^N \nonumber \\&\ \ \ \ + 2 y_*^2 \sum _{N=0}^\infty \left( \left( \omega \rho (\rho -\omega ) \right) _N+\left( \omega \rho (\rho -\omega )\right) _{N-1} \right) (\delta z)^N, \end{aligned}$$

where, by definition $\rho _k=\omega _k=0$ for $k<0$. Equating the coefficients above, we conclude that for any non-negative *N* we have

2.36 $$\begin{aligned}&(N+1)\rho _{N+1} - y_*^2 \left( \sum _{k+\ell = N}(k+1)\rho _{k+1}(\omega ^2)_\ell + 2\sum _{k+\ell = N-1}(k+1)\rho _{k+1}(\omega ^2)_\ell \right. \nonumber \\&\quad \left. + \sum _{k+\ell = N-2}(k+1)\rho _{k+1}(\omega ^2)_\ell \right) \nonumber \\&\quad + 2 y_*^2 \left( \left( \omega \rho (\rho -\omega )\right) _N +\left( \omega \rho (\rho -\omega )\right) _{N-1} \right) = 0, \end{aligned}$$

which is precisely (2.33).

To prove (2.34) we first note that

$$\begin{aligned} \frac{1}{1+\delta z}= \sum _{m=0}^\infty (-1)^m(\delta z)^m \end{aligned}$$

and therefore

2.37 $$\begin{aligned}&\left( 1-3\omega \right) (1-y_*^2z^2\omega ^2)\frac{1}{1+\delta z} \nonumber \\&= \left( 1-3\sum _{k=0}^\infty \omega _k(\delta z)^k\right) \left( 1 - y_*^2\sum _{\ell =0}^\infty (\omega ^2)_\ell \left( (\delta z)^{\ell +2}+2(\delta z)^{\ell +1}+(\delta z)^\ell \right) \right) \sum _{m=0}^\infty (-1)^m(\delta z)^m \nonumber \\&= \Big (1 - 3\sum _{k=0}^\infty \omega _k(\delta z)^k - y_*^2 \sum _{\ell =0}^\infty (\omega ^2)_\ell \left( (\delta z)^{\ell +2}+2(\delta z)^{\ell +1} +(\delta z)^\ell \right) \nonumber \\&\ \ \ \ + 3 y_*^2 \sum _{k=0}^\infty \omega _k(\delta z)^k \sum _{\ell =0}^\infty (\omega ^2)_\ell \left( (\delta z)^{\ell +2}+2(\delta z)^{\ell +1} +(\delta z)^\ell \right) \Big ) \times \sum _{m=0}^\infty (-1)^m(\delta z)^m \nonumber \\&= \sum _{N=0}^\infty (-1)^N(\delta z)^N - 3 \sum _{N=0}^\infty \sum _{k+m=N} \omega _k(-1)^m (\delta z)^N \nonumber \\&\ \ \ \ - y_*^2 \left( \sum _{\ell +m=N-2}(-1)^m(\omega ^2)_\ell +2\sum _{\ell +m=N-1}(-1)^m(\omega ^2)_\ell +\sum _{\ell +m=N}(-1)^m(\omega ^2)_\ell \right) (\delta z)^N \nonumber \\&\ \ \ \ + 3y_*^2 \left( \sum _{k+\ell +m=N-2}(-1)^m\omega _k(\omega ^2)_\ell + 2 \sum _{k+\ell +m=N-1}(-1)^m\omega _k(\omega ^2)_\ell \right. \nonumber \\&\quad \left. + \sum _{k+\ell +m=N}(-1)^m\omega _k(\omega ^2)_\ell \right) (\delta z)^N. \end{aligned}$$

We plug in (1.27) into (2.31) and obtain

$$\begin{aligned} 0&= \left( \sum _{k=0}^\infty k \omega _k (\delta z)^{k-1}\right) \left( 1 - y_*^2\sum _{\ell =0}^\infty (\omega ^2)_\ell \left( (\delta z)^{\ell +2}+2(\delta z)^{\ell +1} +(\delta z)^\ell \right) \right) \\&\ \ \ \ -\sum _{N=0}^\infty \left( \left( 1-3\omega \right) (1-y_*^2z^2\omega ^2)\frac{1}{1+\delta z} \right) _N(\delta z)^N \\&\ \ \ \ - 2 y_*^2 \sum _{N=0}^\infty \left( \left( \omega ^2(\rho -\omega )\right) _N+\left( \omega ^2(\rho -\omega ) \right) _{N-1} \right) (\delta z)^N \\&= \sum _{N=0}^\infty (N+1)\omega _{N+1}(\delta z)^N \\&\ \ \ \ - y_*^2 \sum _{N=0}^\infty \left( \sum _{k+\ell = N}(k+1)\omega _{k+1}(\omega ^2)_\ell + 2\sum _{k+\ell = N-1}(k+1)\omega _{k+1}(\omega ^2)_\ell \right. \\&\ \ \ \ \left. + \sum _{k+\ell = N-2}(k+1)\omega _{k+1}(\omega ^2)_\ell \right) (\delta z)^N \\&\ \ \ \ -\sum _{N=0}^\infty \left( \left( 1-3\omega \right) (1-y_*^2z^2\omega ^2)\frac{1}{1+\delta z} \right) _N(\delta z)^N \\&\ \ \ \ - 2 y_*^2 \sum _{N=0}^\infty \left( \left( \omega ^2(\rho -\omega )\right) _N+\left( \omega ^2(\rho -\omega )\right) _{N-1} \right) (\delta z)^N \end{aligned}$$

Equating the coefficients and using (2.37), we conclude that for any non-negative *N* we have

$$\begin{aligned}&0 = (N+1)\omega _{N+1} - y_*^2 \left( \sum _{k+\ell = N}(k+1)\omega _{k+1}(\omega ^2)_\ell + 2\sum _{k+\ell = N-1}(k+1)\omega _{k+1}(\omega ^2)_\ell + \sum _{k+\ell = N-2}(k+1)\omega _{k+1}(\omega ^2)_\ell \right) \\&- (-1)^N + 3 \sum _{k+m=N}\omega _k(-1)^m + y_*^2 \left( \sum _{\ell +m=N-2}(-1)^m(\omega ^2)_\ell +2\sum _{\ell +m=N-1}(-1)^m(\omega ^2)_\ell +\sum _{\ell +m=N} (-1)^m(\omega ^2)_\ell \right) \\&- 3y_*^2 \left( \sum _{k+\ell +m=N-2}(-1)^m\omega _k(\omega ^2)_\ell + 2 \sum _{k+\ell +m=N-1}(-1)^m\omega _k(\omega ^2)_\ell + \sum _{k+\ell +m=N}(-1)^m\omega _k(\omega ^2)_\ell \right) \\&- 2y_*^2 \left( \left( \omega ^2(\rho -\omega )\right) _N+\left( \omega ^2(\rho -\omega )\right) _{N-1} \right) , \end{aligned}$$

which is precisely (2.34). $\quad \square$

#### Lemma 2.2

The coefficients $(\rho _i,\omega _i)$, $i=0,1$ satisfy the following formulas:

$$\begin{aligned}&\rho _0 = \omega _0 = \frac{1}{y_*}, \\&\quad \text {Either} \ \ (\rho _1,\omega _1) = (-\omega _0, 1-2\omega _0) \ \ \text { or } \ \ (\rho _1,\omega _1) = (1-3\omega _0, 0) \end{aligned}$$

#### Proof

Letting $N=0$ in (2.33)–(2.34) respectively we obtain

$$\begin{aligned} \left( 1-y_*^2\omega _0^2\right) \rho _1+2y_*^2\omega _0^2(\rho _0-\omega _0)&= 0 \\ \left( 1-y_*^2\omega _0^2\right) \left( \omega _1-1+3\omega _0\right) - 2y_*^2\omega _0^2(\rho _0-\omega _0)&=0. \end{aligned}$$

This is of course consistent with the 0-th order sonic point condition (1.25). We now let $N=1$ in  (2.33)–(2.34) and obtain respectively

2.38 $$\begin{aligned} 2\omega _1\left( \frac{\rho _1}{\omega _0}+1\right)&= 0 \end{aligned}$$

2.39 $$\begin{aligned} \frac{\omega _1^2}{\omega _0}-\frac{\omega _1}{\omega _0}+ 3\omega _1+3\omega _0+\rho _1-1&=0. \end{aligned}$$

From (2.38) we have two possibilities: either $\rho _1=-\omega _0$ or $\omega _1=0$. If $\rho _1=-\omega _0$ we obtain from (2.39)

$$\begin{aligned} 0&= \frac{\omega _1^2}{\omega _0}-\frac{\omega _1}{\omega _0}+ 3\omega _1+2\omega _0-1 = \left( \omega _1 + 2\omega _0-1\right) \left( \frac{\omega _1}{\omega _0}+1\right) . \end{aligned}$$

In this case $\omega _1 = 1- 2 \omega _0$ (which corresponds to the Larson–Penston solution) or $\omega _1 = - \omega _0.$ We disregard the latter possibility as it corresponds to a trivial solution that appears due to multiplication of (1.23)–(1.24) by $1-y_*^2z^2\omega ^2$. If on the other hand $\omega _1=0$ we obtain $\rho _1 = 1-3\omega _0.$ from (2.39). $\quad \square$

By a careful tracking of top-order terms in Lemma 2.1 we will next express $(\rho _N,\omega _N)$ as a function of the Taylor coefficients with indices less or equal to $N-1$.

#### Lemma 2.3

Let $N\ge 2$. Then the following identity holds:

$$\begin{aligned} {\mathcal {A}}_N(\omega _0,\omega _1,\rho _1) \begin{pmatrix} \rho _N \\ \omega _N \end{pmatrix} = \begin{pmatrix} {\mathcal {F}}_N \\ {\mathcal {G}}_N \end{pmatrix}, \end{aligned}$$

where

2.40 $$\begin{aligned} {\mathcal {A}}_N(\omega _0,\omega _1,\rho _1) = \begin{pmatrix} -2N+2-2N\frac{\omega _1}{\omega _0} &{} -\frac{2\rho _1}{\omega _0}-2 \\ -2 &{} -2N-4+\frac{2}{\omega _0}-(2N+2)\frac{\omega _1}{\omega _0} \end{pmatrix} \end{aligned}$$

and

$$\begin{aligned} {\mathcal {F}}_N&= {\mathcal {F}}_N[\rho _0,\omega _0;\rho _1, \omega _1;\dots \rho _{N-1},\omega _{N-1}] \\ {\mathcal {G}}_N&= {\mathcal {G}}_N[\rho _0,\omega _0;\rho _1,\omega _1;\dots \rho _{N-1},\omega _{N-1}] \end{aligned}$$

are nonlinear polynomials of the first $N-1$ Taylor coefficients given explicitly by the formulas (2.42) and (2.44) below.

#### Proof

We first isolate all the coefficients in (2.36) that contain contributions from vectors $(\rho _{N+1},\omega _{N+1})$ and $(\rho _{N},\omega _{N})$. For $N\ge 2$ we obtain

2.41 $$\begin{aligned} 0&= (N+1)\rho _{N+1} - y_*^2\left( (N+1)\rho _{N+1}(\omega ^2)_0 + 2N \rho _N\omega _0\omega _1 +2 \rho _1 \omega _0\omega _N + 2 N \rho _N (\omega ^2)_0\right) \nonumber \\&\ \ \ \ + 2y_*^2 \omega _0\rho _0(\rho _N-\omega _N) - {\mathcal {F}}_N \nonumber \\&= (N+1)\rho _{N+1} - \omega _0^{-2}\left( (N+1)\rho _{N+1}\omega _0^2 + 2N \rho _N\omega _0\omega _1 +2 \rho _1 \omega _0\omega _N + 2 N \rho _N \omega _0^2\right) \nonumber \\&\ \ \ \ + 2\omega _0^{-2} \omega _0\rho _0(\rho _N-\omega _N) - {\mathcal {F}}_N \nonumber \\&= \left( -2N+2 - 2N\frac{\omega _1}{\omega _0}\right) \rho _N + \left( -\frac{2\rho _1}{\omega _0}-2\right) \omega _N - {\mathcal {F}}_N \end{aligned}$$

where we have used

2.42 $$\begin{aligned} {\mathcal {F}}_N = ~&y_*^2 \left( \sum _{\begin{array}{c} k+\ell = N \\ 0<k<N-1 \end{array}}(k+1)\rho _{k+1}(\omega ^2)_\ell + \sum _{\begin{array}{c} m+n=N \\ 0<m<N \end{array}}\rho _1\omega _m\omega _n + 2\sum _{\begin{array}{c} k+\ell = N-1 \\ k<N-1 \end{array}}(k+1)\rho _{k+1}(\omega ^2)_\ell \right. \nonumber \\&\quad \left. + \sum _{k+\ell = N-2}(k+1)\rho _{k+1}(\omega ^2)_\ell \right) \nonumber \\&+ 2y_*^2 \left( \sum _{\begin{array}{c} k+\ell +n=N \\ 0<n<N \end{array}}\omega _k \rho _\ell (\rho _n-\omega _n)+\left( \omega \rho (\rho -\omega )\right) _{N-1} \right) . \end{aligned}$$

We now isolate all the coefficients in (2.34) that contain contributions from vectors $(\rho _{N+1},\omega _{N+1})$ and $(\rho _{N},\omega _{N})$. For $N\ge 2$ we obtain

2.43 $$\begin{aligned} 0&= (N+1)\omega _{N+1} -y_*^2\left( (N+1)\omega _{N+1}\omega _0^2 + 2N \omega _0\omega _1\omega _N+2\omega _0\omega _1\omega _N + 2N \omega _0^2 \omega _N\right) \nonumber \\&\ \ \ \ - 2y_*^2 \omega _0^2\left( \rho _N-\omega _N\right) + 3\omega _N + y_*^2\left( 2\omega _0\omega _N-9\omega _0^2\omega _N\right) - {\mathcal {G}}_N \nonumber \\&= -2\rho _N +\left( -2N-4+\frac{2}{\omega _0}-(2N+2)\frac{\omega _1}{\omega _0}\right) \omega _N - {\mathcal {G}}_N, \end{aligned}$$

where

2.44 $$\begin{aligned} {\mathcal {G}}_N =&y_*^2 \left( \sum _{\begin{array}{c} k+\ell = N \\ 0<k<N-1 \end{array}}(k+1)\omega _{k+1}(\omega ^2)_\ell + \sum _{\begin{array}{c} m+n=N \\ 0<m<N \end{array}}\omega _1\omega _m\omega _n + 2\sum _{\begin{array}{c} k+\ell = N-1 \\ k<N-1 \end{array}}(k+1)\omega _{k+1}(\omega ^2)_\ell + \sum _{k+\ell = N-2}(k+1)\omega _{k+1}(\omega ^2)_\ell \right) \nonumber \\&- 2y_*^2 \left( \sum _{\begin{array}{c} k+\ell +n=N \\ 0<n<N \end{array}}\omega _k \omega _\ell (\rho _n-\omega _n)+\left( \omega ^2(\rho -\omega )\right) _{N-1} \right) + (-1)^N - 3 \sum _{\begin{array}{c} k+m=N\\ k<N \end{array}}\omega _k(-1)^m \nonumber \\&- y_*^2 \left( \sum _{\ell +m=N-2}(-1)^m(\omega ^2)_\ell +2\sum _{\ell +m=N-1}(-1)^m(\omega ^2)_\ell +\sum _{\begin{array}{c} \ell +m=N \\ \ell<N \end{array}}(-1)^m(\omega ^2)_\ell + \sum _{\begin{array}{c} k+n=N \\ 0<k<N \end{array}}\omega _k\omega _n\right) \nonumber \\&+ 3y_*^2 \Big (\sum _{k+\ell +m=N-2}(-1)^m\omega _k(\omega ^2)_\ell + 2 \sum _{k+\ell +m=N-1}(-1)^m\omega _k(\omega ^2)_\ell + \sum _{\begin{array}{c} k+\ell +m=N \\ k\ne N, \ell \ne N \end{array}}(-1)^m\omega _k(\omega ^2)_\ell + \omega _0\sum _{\begin{array}{c} k+\ell =N\\ 0<k<N \end{array}}\omega _k\omega _\ell \Big ) . \end{aligned}$$

Finally, Eqs. (2.41) and (2.43) give (2.40). $\quad \square$

#### Lemma 2.4

Let $y_*>0$ be given. Then the matrix

$$\begin{aligned} {\mathcal {A}}_N^{LP} : = {\mathcal {A}}_N(\omega _0,-\omega _0,1-2\omega _0) \end{aligned}$$

associated with an LP-type solution is singular if and only if

2.45 $$\begin{aligned} y_*=1 \ \ \text { or } \ \ y_*= \frac{N+1}{N}, \ \ \text { for some } N\ge 2. \end{aligned}$$

As a consequence, the matrix ${\mathcal {A}}_N^{LP}$ is invertible for any $y_*>\frac{3}{2}$ for all $N\ge 2$.

#### Proof

Since in the case of LP-type solutions (i.e. $(\rho _1,\omega _1) = (-\omega _0, 1-2\omega _0)$) the matrix ${\mathcal {A}}_N$ takes the form

$$\begin{aligned} {\mathcal {A}}_N^{LP} = 2\begin{pmatrix} N(1-\frac{1}{\omega _0})+1 &{} 0 \\ -1 &{} N(1-\frac{1}{\omega _0}) \end{pmatrix} \end{aligned}$$

In particular

$$\begin{aligned} \frac{1}{4}\det {\mathcal {A}}^{LP}_N&= N\left( N(1-\frac{1}{\omega _0})+1 \right) \left( 1-\frac{1}{\omega _0}\right) . \end{aligned}$$

Therefore, the matrix ${\mathcal {A}}_N^{LP}$ is singular when $\omega _0=1$ or $\omega _0=\frac{N}{N+1}$, $N\ge 2$. This together with (1.25) implies (2.45). Since $\frac{N}{N+1}\ge \frac{2}{3}$ for all $N\ge 2$ ${\mathcal {A}}_N^{LP}$ is invertible for any $0<\omega _0<\frac{2}{3}$ and $N\ge 2$. $\quad \square$

#### Remark 2.5

(Hunter solutions) In the case of Hunter-type solutions (i.e. $(\rho _1,\omega _1) = (1-3\omega _0, 0)$) the matrix ${\mathcal {A}}_N$ takes the form

$$\begin{aligned} {\mathcal {A}}_N^{H} = 2\begin{pmatrix} -N+1 &{} 2-\frac{1}{\omega _0} \\ -1 &{} -N-2+\frac{1}{\omega _0} \end{pmatrix} \end{aligned}$$

In particular

2.46 $$\begin{aligned} \frac{1}{4}\det {\mathcal {A}}^{H}_N&= (N-1)(N+2-\frac{1}{\omega _0}) +2-\frac{1}{\omega _0} \nonumber \\&= N (N+1-\frac{1}{\omega _0}) \end{aligned}$$

It follows that the matrix ${\mathcal {A}}_N^H$ is singular if and only if $y_*=N+1$ for some $N\ge 2$.

For any $y_*>\frac{3}{2}$ consider the formal series (1.27) of LP-type, i.e. with conditions (1.25) and (1.28) satisfied. By Lemmas 2.3 and 2.4 we have the explicit relations

2.47 $$\begin{aligned} \rho _N&= \frac{1}{2\left( N(1-\frac{1}{\omega _0})+1\right) } {\mathcal {F}}_N \end{aligned}$$

2.48 $$\begin{aligned} \omega _N&= \frac{1}{2N(1-\frac{1}{\omega _0})} {\mathcal {G}}_N + \frac{1}{2N(1-\frac{1}{\omega _0})\left( N(1-\frac{1}{\omega _0})+1\right) } {\mathcal {F}}_N \end{aligned}$$

The assumption $\omega _0<\frac{2}{3}$ [recall $\omega _0=\frac{1}{y_*}$ by (1.25)] implies that there exists a universal constant $\alpha >0$ such that

2.49 $$\begin{aligned} \left| \rho _N\right|&\le \frac{\alpha }{N\left( \frac{2}{3}-\omega _0\right) } \left| {\mathcal {F}}_N\right| \end{aligned}$$

2.50 $$\begin{aligned} \left| \omega _N\right|&\le \frac{\alpha }{N\left( \frac{2}{3}-\omega _0\right) } \left( \left| {\mathcal {G}}_N\right| + \frac{1}{N} \left| {\mathcal {F}}_N\right| \right) . \end{aligned}$$

Our goal is to show that the formal power series $\sum _{N=0}^\infty \rho _N(\delta z)^N$, $\sum _{N=0}^\infty \omega _N(\delta z)^N$ converge. To that end we need some simple technical bounds which will be important in establishing convergence later on.

#### Lemma 2.6

There exists a constant $c>0$ such that for all $N\in {\mathbb {N}}$ the following bounds hold

2.51 $$\begin{aligned} \sum _{k=1}^{N-1}\frac{1}{k^2(N-k)^2}&\le c N^{-2} \end{aligned}$$

2.52 $$\begin{aligned} \sum _{\begin{array}{c} k+\ell + m = N \\ 0<k,\ell ,m \end{array}}\frac{1}{k^2\ell ^2m^2}&\le c N^{-2}, \end{aligned}$$

2.53 $$\begin{aligned} \sum _{\begin{array}{c} k+\ell + m = N \\ 0<k,\ell ,m \end{array}}\frac{1}{k\ell ^2m^2}&\le c N^{-1}. \end{aligned}$$

#### Proof

We note that

$$\begin{aligned} \sum _{k=1}^{N-1}\frac{1}{k^2(N-k)^2} = \sum _{k=1}^{N-1}\frac{1}{N^2}\left( \frac{1}{k}+\frac{1}{N-k}\right) ^2 \le \frac{2}{N^2}\sum _{k=1}^\infty \frac{1}{k^2} \lesssim N^{-2} \end{aligned}$$

and this proves (2.51). Next

$$\begin{aligned} \sum _{\begin{array}{c} k+\ell + m = N \\ 0<k,\ell ,m \end{array}}\frac{1}{k^2\ell ^2m^2}&\le \sum _{k=1}^{N-1}\frac{1}{k^2}\sum _{\begin{array}{c} \ell +m=N-k \\ 0<\ell ,m \end{array}} \frac{1}{\ell ^2m^2} \lesssim \sum _{k=1}^N\frac{1}{k^2}\frac{1}{(N-k)^2} \lesssim N^{-2}, \end{aligned}$$

where we have used the already established bound (2.51) in each of the last two lines above. This proves (2.52). Finally,

$$\begin{aligned} \sum _{\begin{array}{c} k+\ell + m = N \\ 0<k,\ell ,m \end{array}}\frac{1}{k\ell ^2m^2}&\le \sum _{k=1}^N\frac{1}{k}\sum _{\begin{array}{c} \ell +m=N-k \\ 0<\ell ,m \end{array}} \frac{1}{\ell ^2m^2} \lesssim \sum _{k=1}^N\frac{1}{k}\frac{1}{(N-k)^2}\\&= \sum _{k=1}^N\frac{1}{N} \left( \frac{1}{k}+\frac{1}{N-k}\right) \frac{1}{N-k} \lesssim \frac{1}{N}, \end{aligned}$$

and this completes the proof of (2.53). $\quad \square$

Let $M\ge 1$ be such that

2.54 $$\begin{aligned} |\rho _0|, \ |\omega _0|, \ |\rho _1|, \ |\omega _1| <M. \end{aligned}$$

#### Lemma 2.7

Let $y_*>\frac{3}{2}$ and $\alpha \in (1,2)$. Assume that

2.55 $$\begin{aligned} \left| \rho _k \right|&\le \frac{C^{k-\alpha }}{k^2}, \ \ 2\le k \le N-1 \end{aligned}$$

2.56 $$\begin{aligned} \left| \omega _k \right|&\le \frac{C^{k-\alpha }}{k^2}, \ \ 2\le k \le N-1, \end{aligned}$$

for some $C\ge 1$ and $N\ge 3$. Then there exists a constant $D=D(M)>M$ such that

2.57 $$\begin{aligned} |(\omega ^2)_\ell | +\left| (\omega \rho )_\ell \right| +|(\rho ^2)_\ell | \le {\left\{ \begin{array}{ll} D &{} \ \text { if } \ \ell =0,1 \\ D + D \frac{C^{\ell -\alpha }}{\ell ^2} &{} \ \text { if } \ \ell =2\\ D \frac{C^{\ell -\alpha }}{\ell ^2} &{} \ \text { if } \ 3\le \ell \le N-1. \end{array}\right. } \end{aligned}$$

#### Proof

We first prove the bounds for $|(\omega ^2)_\ell |$, $\ell \ge 0$. The bounds $|(\omega ^2)_0|\le M^2$ and $|(\omega ^2)_1|\le 2M^2$ are obvious from (2.54). Clearly

2.58 $$\begin{aligned} |(\omega ^2)_2|\le 2M|\omega _2|+M^2 \le 2M \frac{C^{2-\alpha }}{2^2}+M^2. \end{aligned}$$

If $\ell \ge 3$ we then have

2.59 $$\begin{aligned} |(\omega ^2)_\ell |&\le \sum _{k=0}^\ell |\omega _k||\omega _{\ell -k}| \le 2|\omega _0||\omega _\ell | + 2|\omega _1||\omega _{\ell -1}| +2 \sum _{k=2}^{\ell -2}|\omega _k||\omega _{\ell -k}| \nonumber \\&\le 2M \frac{C^{\ell -\alpha }}{\ell ^2} + 2M \frac{C^{\ell -1-\alpha }}{(\ell -1)^2} + 2\sum _{k=2}^{\ell -2} \frac{C^{\ell -2\alpha }}{k^2(\ell -k)^2} \nonumber \\&\le 2M C^{\ell -\alpha } \left( \frac{1}{\ell ^2} + \frac{1}{(\ell -1)^2} + \frac{1}{M}\sum _{k=2}^{\ell -2} \frac{1}{k^2(\ell -k)^2} \right) \nonumber \\&\le 2M {{\tilde{C}}} \frac{C^{\ell -\alpha }}{\ell ^2}, \end{aligned}$$

for some universal constant ${{\tilde{C}}}$. It is now clear, that the estimates for $(\omega \rho )_\ell$ and $(\rho ^2)_\ell$, $\ell \ge 0$ follow in the same way, as the only estimates we have used are (2.54) and the inductive assumptions (2.55)–(2.56), which both depend only on the index, and are symmetric with respect to $\rho$ and $\omega$. Finally, from (2.58) it is clear that $D\ge M^2\ge M$ since $M\ge 1$. $\quad \square$

#### Lemma 2.8

Let $y_*>\frac{3}{2}$ and $\alpha \in (1,2)$. Then there exists a constant $C_*>0$ such that if $C>C_*$ and for any $N\ge 3$ the following assumptions hold:

2.60 $$\begin{aligned} \left| \rho _k \right|&\le \frac{C^{k-\alpha }}{k^2}, \ \ 2\le k \le N-1 \end{aligned}$$

2.61 $$\begin{aligned} \left| \omega _k \right|&\le \frac{C^{k-\alpha }}{k^2}, \ \ 2\le k \le N-1, \end{aligned}$$

then there exists a constant ${{\tilde{c}}}={{\tilde{c}}}(D)$ such that

2.62 $$\begin{aligned} \left| {\mathcal {F}}_N\right|&\le \frac{{{\tilde{c}}}}{N} C^{N-\alpha } \left( \frac{1}{CN} + \frac{1}{C} + \frac{1}{C^{\alpha -1}}\right) , \end{aligned}$$

2.63 $$\begin{aligned} \left| {\mathcal {G}}_N\right|&\le \frac{{{\tilde{c}}}}{N} C^{N-\alpha }\left( \frac{1}{CN} + \frac{1}{C} + \frac{1}{C^{\alpha -1}}\right) . \end{aligned}$$

#### Proof

By the assumption (2.54) and the bound $D\ge M$ we trivially have

2.64 $$\begin{aligned} |\rho _0|, \ |\omega _0|, \ |\rho _1|, \ |\omega _1| <D, \end{aligned}$$

where *D* is the constant from Lemma 2.7. In the following, the constant *c* is a generic constant which depends on *D*, but not on *N*, and may change from line to line. Throughout the proof we use the convention that any summation of the form $\sum _{k=a}^b$ with $a>b$ is zero.

We start with the estimate on the first term in the definition (2.42) of ${\mathcal {F}}_N$.

2.65 $$\begin{aligned} \sum _{\begin{array}{c} k+\ell = N \\ 0<k<N-1 \end{array}}(k+1)\rho _{k+1}(\omega ^2)_\ell&\le (N-1)|\rho _{N-1}| |(\omega ^2)_2 | + \sum _{k=1}^{N-3}(k+1) |\rho _{k+1}| |(\omega ^2)_{N-k}| \nonumber \\&\le \frac{DC^{N-1-\alpha }(1+ \frac{C^{2-\alpha }}{2^2})}{N-1} + DC^{N+1-2\alpha } \sum _{k=1}^{N-3}\frac{1}{k+1}\frac{1}{(N-k)^2} \nonumber \\&\le c \frac{C^{N+1-2\alpha }}{N}, \end{aligned}$$

where we have used Lemma 2.7 to estimate $|(\omega ^2)_2|$ and $|(\omega ^2)_\ell |$, $\ell \ge 3$, and the inductive assumption (2.61) in the second line. To obtain the bound in the third line we used the estimate (2.53), and the bound $1<\frac{C^{2-\alpha }}{2^2}$ which holds for *C* sufficiently large since $\alpha <2$.

2.66 $$\begin{aligned}&\left| \sum _{\begin{array}{c} k+\ell = N-1 \\ k<N-1 \end{array}}(k+1)\rho _{k+1}(\omega ^2)_\ell \right| \le |\rho _1| |(\omega ^2)_{N-1} | + (N-1)|\rho _{N-1}||(\omega ^2)_1|+ \sum _{k=1}^{N-3}(k+1)|\rho _{k+1}| |(\omega ^2)_{N-1-k}| \nonumber \\&\le D^2 \frac{C^{N-1-\alpha }}{(N-1)^2} + D \frac{C^{N-1-\alpha }}{N-1}+DC^{N-2\alpha }\sum _{k=1}^{N-3}\frac{1}{(k+1)(N-1-k)^2} \nonumber \\&\le c \frac{C^{N-1-\alpha }}{N^2} + \frac{c}{C} \frac{C^{N-\alpha }}{N}, \end{aligned}$$

where we have used Lemma 2.7, bound (2.64), the inductive assumptions (2.60)–(2.56) in the second line, and the bounds $C^{N-2\alpha }\le C^{N-\alpha }$, (2.53) in the third. A similar argument gives us

2.67 $$\begin{aligned}&\left| \sum _{k+\ell = N-2}(k+1)\rho _{k+1}(\omega ^2)_\ell \right| \nonumber \\&\le |\rho _1| |(\omega ^2)_{N-2}| + (N-1) |\rho _{N-1}| |(\omega ^2)_{0}| + (N-2)|\rho _{N-2}| |(\omega ^2)_{1}|+ \sum _{k=1}^{N-4}(k+1) |\rho _{k+1}| |(\omega ^2)_{N-2-k}| \nonumber \\&\le D^2 \frac{C^{N-2-\alpha }}{(N-2)^2} + D \frac{C^{N-1-\alpha }}{N-1} + D \frac{C^{N-2-\alpha }}{N-2} + DC^{N-1-2\alpha }\sum _{k=1}^{N-4} \frac{1}{(k+1)(N-2-k)^2} \nonumber \\&\le \frac{c}{C} \frac{C^{N-\alpha }}{N}, \end{aligned}$$

where we have used Lemma 2.7, bound (2.64), the inductive assumptions (2.55)–(2.61), and the bound $\sum _{k=1}^{N-4}\frac{1}{(k+1)(N-2-k)^2} \lesssim \frac{1}{N-1}\lesssim \frac{1}{N}$.

In a similar way

2.68 $$\begin{aligned}&\left| \sum _{\begin{array}{c} k+\ell +n=N \\ 0<n<N \end{array}}\omega _k\rho _\ell (\rho _n-\omega _n)\right| = \left| \sum _{n=1}^{N-1}(\rho \omega )_{N-n} (\rho _{n}-\omega _{n}) \right| \nonumber \\&\le \left( |\rho _1|+|\omega _1|\right) \left| (\rho \omega )_{N-1} \right| + \left( |\rho _{N-1}|+|\omega _{N-1}|\right) \left| (\rho \omega )_{1} \right| + \sum _{n=2}^{N-2}\left| (\rho \omega )_{N-n} \right| (|\rho _{n}|+|\omega _{n}|) \nonumber \\&\le 2 D^2 \frac{C^{N-1-\alpha }}{(N-1)^2} + 2D\frac{C^{N-1-\alpha }}{(N-1)^2} +2C^{N-2\alpha } \sum _{n=2}^{N-2} \frac{1}{n^2}\frac{1}{(N-n)^2} \nonumber \\&\le c \frac{C^{N-1-\alpha }}{N^2}, \end{aligned}$$

where we have used the Lemma 2.7, the inductive assumptions (2.60)–(2.56), and the bound (2.51). In an entirely analogous way we obtain the bound

2.69 $$\begin{aligned} \left| \left( \omega \rho (\rho -\omega )\right) _{N-1}\right| \le \frac{c}{C} \frac{C^{N-\alpha }}{N^2} \end{aligned}$$

Finally, using the bound (2.64) and the inductive assumptions (2.55)–(2.61), we obtain

2.70 $$\begin{aligned} \left| \sum _{\begin{array}{c} m+n=N \\ 0<m<N \end{array}}\rho _1\omega _m\omega _n \right|&\le 2|\rho _1| |\omega _1||\omega _{N-1}| + |\rho _1|\sum _{m=2}^{N-2}| \omega _m||\omega _{N-m}| \nonumber \\&\le 2D^2 \frac{C^{N-1-\alpha }}{(N-1)^2} + D C^{N-2\alpha } \sum _{m=2}^{N-2}\frac{1}{m^2(N-m)^2} \nonumber \\&\le c \frac{C^{N-1-\alpha }}{N^2} \end{aligned}$$

where we have used (2.51), bound (2.64), and the inductive assumptions. From the definition (2.42) of ${\mathcal {F}}_N$ and bounds (2.65)–(2.70) we conclude that (2.62) holds.

We now turn our attention to the source term ${\mathcal {G}}_N$. Note that the first line of (2.44) can be transformed into the first line of (2.42) by formally replacing some of the $\rho _k$-s with $\omega _k$-s. Similarly, the negative of the second line of (2.44) is formally equal to the second line of (2.42) after formally replacing some of the $\rho _k$-s and $\omega _k$-s. Since our estimates only depend on the bounds (2.64) and the inductive assumptions (2.60)–(2.56)—which only depend on the index of $\rho$ and $\omega$ and are therefore invariant under the formal exchange of $\rho$ and $\omega$—the estimates analogous to (2.65)–(2.70) imply that the first two lines of (2.44) are bounded by

2.71 $$\begin{aligned} \frac{c}{N} C^{N-\alpha }\left( \frac{1}{CN} + \frac{1}{C} + \frac{1}{C^{\alpha -1}}\right) . \end{aligned}$$

Clearly

2.72 $$\begin{aligned} \left| (-1)^N - 3 \sum _{\begin{array}{c} k+m=N\\ k<N \end{array}}\omega _k(-1)^m \right|&\lesssim 1 + \sum _{k=0}^{N-1} \left| \omega _k\right| \lesssim 1+ \sum _{k=2}^{N-1} \frac{C^{k-\alpha }}{k^2} \nonumber \\&= 1 + C^{-\alpha } \sum _{k=2}^{\lfloor \frac{N}{2}\rfloor } \frac{C^k}{k^2}+ C^{-\alpha }\sum _{k=\lfloor \frac{N}{2}\rfloor +1}^{N-1} \frac{C^{k}}{k^2} \nonumber \\&\lesssim 1 + C^{\lfloor \frac{N}{2}\rfloor -\alpha } + \frac{C^{N-1-\alpha }}{N} \le c \frac{C^{N-1-\alpha }}{N}, \end{aligned}$$

where we note that the last estimate follows from $N\le c C^{N-1-\lfloor \frac{N}{2}\rfloor }$, for some constant $c>0$ and all $N\ge 3$, and $C>1$ independent of *N*. By the proof of (2.72) we have

2.73 $$\begin{aligned} \sum _{\ell =2}^{N-1} \frac{C^{\ell -\alpha }}{\ell ^2} \le c \frac{C^{N-1-\alpha }}{N}, \end{aligned}$$

for $N\ge 3$ and *C* sufficiently large, but independent of *N*.

To bound the quadratic nonlinearities in the 4-th line of (2.44) we note the bound

2.74 $$\begin{aligned} \left| \sum _{\begin{array}{c} \ell +m=N \\ \ell <N \end{array}}(-1)^m(\omega ^2)_\ell \right|&\le 2D +\sum _{\ell =2}^{N-1} \left| (\omega ^2)_\ell \right| \le 2D+ D\sum _{\ell =2}^{N-1} \frac{C^{\ell -\alpha }}{\ell ^2} \le c \frac{C^{N-1-\alpha }}{N}, \end{aligned}$$

where we have used Lemmas 2.7 and (2.73). By the same reasoning we obtain

2.75 $$\begin{aligned} \left| \sum _{\ell +m=N-2}(-1)^m(\omega ^2)_\ell +2\sum _{\ell +m=N-1} (-1)^m(\omega ^2)_\ell \right| \lesssim c \frac{C^{N-1-\alpha }}{N}. \end{aligned}$$

The last term in the fourth line of (2.44) has already been estimated in the proof of (2.70) and we obtain

2.76 $$\begin{aligned} \left| \sum _{\begin{array}{c} k+n=N\\ 0<k<N \end{array}}\omega _k\omega _n\right| \le c \frac{C^{N-1-\alpha }}{N^2}. \end{aligned}$$

It remains to bound the cubic expressions in the last two lines of (2.44). We start with

2.77 $$\begin{aligned}&\left| \sum _{k+\ell +m=N-2}(-1)^m\omega _k(\omega ^2)_\ell \right| \le \sum _{m=0}^{N-2}\sum _{\ell =0}^{N-2-m} |(\omega ^2)_{\ell }| |\omega _{N-2-\ell -m}| \nonumber \\&\le |(\omega ^2)_0| |\omega _0| + \sum _{\ell =0}^1 |(\omega ^2)_{\ell }| |\omega _{1-\ell }| + \sum _{\ell =0}^2 |(\omega ^2)_{\ell }| |\omega _{2-\ell }| + \sum _{m=0}^{N-5}\sum _{\ell =0}^{N-2-m} |(\omega ^2)_{\ell }| |\omega _{N-2-\ell -m}| \nonumber \\&\le 4D^2 + 2D \frac{C^{2-\alpha }}{2^2}+ \sum _{m=0}^{N-5}\sum _{\ell =0}^{N-2-m} |(\omega ^2)_{\ell }| |\omega _{N-2-\ell -m}|. \end{aligned}$$

Here we have used the bound (2.64), Lemma 2.7, and the inductive assumption (2.61). For any $m\le N-5$ we have

2.78 $$\begin{aligned}&\sum _{\ell =0}^{N-2-m} |(\omega ^2)_{\ell }| |\omega _{N-2-\ell -m}| \nonumber \\&\le |(\omega ^2)_{0}| |\omega _{N-2-m}| + |(\omega ^2)_{1}| |\omega _{N-3-m}| + \sum _{\ell =2}^{N-2-m} |(\omega ^2)_{\ell }| |\omega _{N-2-\ell -m}| \nonumber \\&\le D \left( \frac{C^{N-2-m-\alpha }}{(N-2-m)^2}+\frac{C^{N-3-m-\alpha }}{(N-3-m)^2} \right) + \sum _{\ell =2}^{N-4-m} |(\omega ^2)_{\ell }| |\omega _{N-2-\ell -m}| \nonumber \\&\ \ \ \ + |(\omega ^2)_{N-3-m}| |\omega _{1}| + |(\omega ^2)_{N-2-m}| | \omega _{0}| \nonumber \\&\le cD\frac{C^{N-2-m-\alpha }}{(N-m)^2} +D C^{N-2-m-2\alpha } \sum _{\ell =2}^{N-m-4}\frac{1}{\ell ^2(N-2-m-\ell )^2} \nonumber \\&\ \ \ \ + D^2 \frac{C^{N-3-m-\alpha }}{(N-m-3)^2} + D^2 \frac{C^{N-2-m-\alpha }}{(N-m-2)^2} \nonumber \\&\le c \frac{C^{N-2-m-\alpha }}{(N-m)^2}, \end{aligned}$$

where we have used the bound (2.64), Lemma 2.7, the inductive assumptions (2.55)–(2.56), and (2.51). Using (2.78) in (2.77) we obtain

2.79 $$\begin{aligned} \left| \sum _{k+\ell +m=N-2}(-1)^m\omega _k(\omega ^2)_\ell \right|&\le 4D^2 + 2D \frac{C^{2-\alpha }}{2^2} + c C^{-\alpha }\sum _{m=0}^{N-5} \frac{C^{N-2-m}}{(N-m)^2} \nonumber \\&\le c\frac{C^{N-1-\alpha }}{N^2} + c C^{-\alpha }\sum _{k=5}^{N} \frac{C^{k-2}}{k^2}\nonumber \\&\le \frac{c}{N} C^{N-\alpha }\left( \frac{1}{CN}+\frac{1}{C^2}\right) , \end{aligned}$$

where we have used the bound $4D^2 + 2D \frac{C^{2-\alpha }}{2^2} \le c \frac{C^{N-1-\alpha }}{N^2}$ for some constant *c* and all $N\ge 3$, and (2.73) in the last line. By the same proof we obtain

2.80 $$\begin{aligned}&\left| \sum _{k+\ell +m=N-1}(-1)^m\omega _k(\omega ^2)_\ell \right| \le c \frac{C^{N-1-\alpha }}{N} \end{aligned}$$

We next estimate

2.81 $$\begin{aligned}&\left| \sum _{\begin{array}{c} k+\ell +m=N \\ k< N, \ell < N \end{array}}(-1)^m\omega _k(\omega ^2)_\ell \right| \le \sum _{\ell =1}^{N-1}|(\omega ^2)_\ell | |\omega _{N-\ell }| + \sum _{m=1}^{N}\sum _{\ell =0}^{N-m} |(\omega ^2)_{\ell }| |\omega _{N-\ell -m}| \nonumber \\&\le \sum _{\ell =1}^{N-1}|(\omega ^2)_\ell | |\omega _{N-\ell }| + |(\omega ^2)_0 | |\omega _0| + \sum _{\ell =0}^{1}|(\omega ^2)_\ell ||\omega _{1-\ell }| + \sum _{m=1}^{N-2}\sum _{\ell =0}^{N-m} |(\omega ^2)_{\ell }| |\omega _{N-\ell -m}| \nonumber \\&\le c D^2 + \sum _{\ell =1}^{N-1}|(\omega ^2)_\ell | |\omega _{N-\ell }| + \sum _{m=1}^{N-2}\sum _{\ell =0}^{N-m} |(\omega ^2)_{\ell }| |\omega _{N-\ell -m}| , \end{aligned}$$

where we have used Lemma 2.7 and (2.64). Proceeding as in the proof of (2.78) we have

2.82 $$\begin{aligned} \sum _{\ell =0}^{N-m} |(\omega ^2)_{\ell }| |\omega _{N-\ell -m}|\le c \frac{C^{N-m-\alpha }}{(N-m)^2}, \ \ m\le N-2. \end{aligned}$$

On the other hand,

2.83 $$\begin{aligned} \sum _{\ell =1}^{N-1}|(\omega ^2)_\ell | |\omega _{N-\ell }|&= |(\omega ^2)_1 | |\omega _{N-1}| + |(\omega ^2)_{N-1} | |\omega _{1}| + \sum _{\ell =2}^{N-2}|(\omega ^2)_\ell | |\omega _{N-\ell }| \nonumber \\&\le c \frac{C^{N-1-\alpha }}{(N-1)^2} + c C^{N-2\alpha } \sum _{\ell =2}^{N-2}\frac{1}{\ell ^2(N-\ell )^2} \le c \frac{C^{N-1-\alpha }}{N^2},\nonumber \\ \end{aligned}$$

where we have used Lemma 2.7 in the second line, and the bounds $\alpha >1$ and (2.51) in the last line. Using (2.82)–(2.83) in (2.81) we obtain

2.84 $$\begin{aligned}&\left| \sum _{\begin{array}{c} k+\ell +m=N \\ k< N, \ell < N \end{array}}(-1)^m\omega _k(\omega ^2)_\ell \right| \le c D^2 + c \frac{C^{N-1-\alpha }}{N^2} + c\sum _{m=1}^{N-2} \frac{C^{N-m-\alpha }}{(N-m)^2}\nonumber \\&\le c \frac{C^{N-1-\alpha }}{N^2} + c \sum _{k=2}^{N-1} \frac{C^{k-\alpha }}{k^2} \le c \frac{C^{N-1-\alpha }}{N^2} + c \frac{C^{N-1-\alpha }}{N} \nonumber \\&\le \frac{c}{N} C^{N-\alpha }\left( \frac{1}{CN} + \frac{1}{C} \right) . \end{aligned}$$

Finally, the last remaining term to estimate is

2.85 $$\begin{aligned} \left| \omega _0\sum _{\begin{array}{c} k+\ell =N\\ 0<k<N \end{array}}\omega _k\omega _\ell \right| \le D \sum _{k=1}^{N-1}|\omega _k||\omega _{N-k}| \le c \frac{C^{N-1-\alpha }}{N^2}, \end{aligned}$$

where we have used the same argument as in (2.83). From the definition (2.44) of ${\mathcal {G}}_N$ and the bounds (2.71)–(2.72), (2.74)–(2.76), (2.79)–(2.80), and (2.84)–(2.85), we conclude (2.63). $\quad \square$

#### Lemma 2.9

Let $y_*>\frac{3}{2}$ and $\alpha \in (1,2)$. Let $\{\rho _k,\omega _k\}_{k\in {\mathbb {N}}}$ be the coefficients associated with an LP-type solution. Then there exists a constant $C>1$ such that

2.86 $$\begin{aligned} \left| \rho _N\right|&\le \frac{C^{N-\alpha }}{N^2} \end{aligned}$$

2.87 $$\begin{aligned} \left| \omega _N\right|&\le \frac{C^{N-\alpha }}{N^2}. \end{aligned}$$

for all $N\ge 2$. Moreover, for any closed interval $K\subset (0,\frac{2}{3})$ we can choose the same constant *C* for all $\omega _0\in K$.

#### Proof

The proof proceeds by induction. Recall that *c* is a constant which may change from line to line and depends on $y_*,\alpha$, but not on *N*. When $N=2$ it is clear that there exists a $C_0=C_0(y_*,\alpha )>1$ such that for any $C>C_0$ the bound

2.88 $$\begin{aligned} |\rho _2|, \ |\omega _2| \le \frac{C^{2-\alpha }}{2^2}, \end{aligned}$$

is true. Fix an $N\ge 3$ and assume that the claim is true for all $2\le k \le N-1$. That means that the assumptions (2.60)–(2.61) are satisfied and thus by Lemma 2.8 we conclude that (2.62)–(2.63) hold. Bounds (2.49) and (2.62) together give

$$\begin{aligned} \left| \rho _N\right| \le \frac{c}{N} \frac{{{\tilde{c}}}}{N} C^{N-\alpha }\left( \frac{1}{CN} + \frac{1}{C} + \frac{1}{C^{\alpha -1}}\right) \le \frac{c}{C^{\alpha -1}} \frac{C^{N-\alpha }}{N^2}. \end{aligned}$$

Similarly, bounds (2.50) and (2.63) give

$$\begin{aligned} \left| \omega _N\right|&\le \frac{c}{N} \left( \left| {\mathcal {G}}_N\right| + \frac{1}{N} \left| {\mathcal {F}}_N\right| \right) \le \frac{c}{C^{\alpha -1}} \frac{C^{N-\alpha }}{N^2}. \end{aligned}$$

It is now clear that we can choose $C>C_0$ large enough so that (2.86)–(2.87) is true, since $\alpha >1$. Therefore, for a sufficiently large choice of *C* the inductive claim follows. The uniformity statement with respect to a closed subinterval $K\subset (0,\frac{2}{3})$ is clear, as the constant *c* varies continuously as a function of $\omega _0$. $\quad \square$

#### Theorem 2.10

Let $K\subset (0,\frac{2}{3})$ be a closed interval. Let $\{\rho _k,\omega _k\}_{k\in {\mathbb {N}}}$ be the coefficients associated with an LP-type solution. There exists an $1>r=r_K>0$ such that for any $\omega _0=\frac{1}{y_*}\in K$ the formal power series

2.89 $$\begin{aligned} {\varvec{\rho }}(z) : = \sum _{N=0}^\infty \rho _N (\delta z)^N , \ \ {\varvec{\omega }}(z) : = \sum _{N=0}^\infty \rho _N (\delta z)^N \end{aligned}$$

converge for all *z* such that $\left| \delta z\right| =\left| z-1\right| <r$. In particular, functions $\rho (z)$ and $\omega (z)$ are real analytic inside the ball $|z-1|<r$. We can differentiate the infinite sums term by term and $(\rho (z),\omega (z))$ is an LP-type solution of (1.23)–(1.24) for $\left| z-1\right| <r$. Moreover, the density $\rho (\cdot ;y_*)$ is strictly positive for $\left| z-1\right| <r$.

#### Proof

Fix an $\alpha \in (0,2)$. By Lemma 2.9 there exists a $C=C(K,\alpha )>0$ such that

$$\begin{aligned} \sum _{N=1}^\infty \left| \rho _N\right| |\delta z|^N + \sum _{N=1}^\infty \left| \omega _N\right| |\delta z|^N \le 2\sum _{N=1}^\infty \frac{\left| C\delta z\right| ^N}{C^\alpha N^2} <\infty , \end{aligned}$$

when $|\delta z|<\frac{1}{C}=:r$. The claim follows by the comparison test. The real analyticity and differentiability statements are clear. Since

$$\begin{aligned} 1-y_*^2z^2\omega ^2&= 1-y_*^2(1+\delta z)^2\omega ^2 \\&= - 2y_*(1-\omega _0) \delta z - y_*^2 \sum _{N=2}^\infty \left( (\omega ^2)_{N-2}+2(\omega ^2)_{N-1}+(\omega ^2)_N\right) (\delta z)^N \\&\ne 0, \ \ 0<|\delta z| \ll 1, \end{aligned}$$

it follows that for $r>0$ sufficiently small, the function $1-y_*^2z^2\omega ^2\ne 0$ for all $|z-1|<r$ and $z\ne 1$. Functions $\rho (z)$ and $\omega (z)$ are indeed the solutions, as can be seen by plugging the infinite series (2.89) into the left-hand sides of (2.31)–(2.32); all the functions appearing on the left-hand side are analytic for $|z-1|<r$. $\quad \square$

We note that $\rho _i,\omega _i$ are smooth with respect to $\omega _0$ (or equivalently $y_*$) for $i=0,1$ for any $\omega _*>0$. This follows from the explicit formula for the Taylor coefficients around the sonic point for LP-type solutions. We next note that for any $N\ge 2$ we can express $\rho _N, \omega _N$ as polynomial function of $\rho _0,\dots \rho _{N-1}, \omega _0,\dots \omega _{N-1}$ for any $0<\omega _0<\frac{2}{3}$ and therefore it is clear that $\rho _N,\omega _N$ are smooth functions of $\omega _0$ for all $N\in {\mathbb {N}}$ for $\omega _0\in (0,\frac{2}{3})$.

#### Lemma 2.11

Let $\alpha \in (1,2)$. Let $\{\rho _k,\omega _k\}_{k\in {\mathbb {N}}}$ be the coefficients associated with an LP-type solution. For any $\omega _0\in (0,\frac{2}{3})$ there exists a constant $C =C(\omega _0,\alpha )>0$ such that

2.90 $$\begin{aligned} \left| \partial _{\omega _0}\rho _1\right| , \ \left| \partial _{\omega _0}\omega _1\right| \le C, \end{aligned}$$

and for all $N\ge 2$:

2.91 $$\begin{aligned} \left| \partial _{\omega _0}\rho _N\right|&\le \frac{C^{N-\alpha }}{N^2} \end{aligned}$$

2.92 $$\begin{aligned} \left| \partial _{\omega _0}\omega _N\right|&\le \frac{C^{N-\alpha }}{N^2} . \end{aligned}$$

In particular, there exists an $r>0$ such that the formal power series

$$\begin{aligned} \sum _{N=0}^\infty \partial _{\omega _0}\rho _N (\delta z)^N, \ \ \sum _{N=0}^\infty \partial _{\omega _0}\omega _N (\delta z)^N, \end{aligned}$$

converge for all *z* satisfying $|z-1|<r$. Moreover, the function $(0,\frac{2}{3})\ni \omega _0\rightarrow (\rho (z;\omega _0),\omega (z,\omega _0)$ is $C^1$ and the derivatives $\partial _{\omega _0}\rho$ and $\partial _{\omega _0}\omega$ are given by the infinite series above.

#### Proof

When $N=1$ we have by the Larson-Penston sonic condition (1.28) $\partial _{\omega _0}\rho _1 = -1$, $\partial _{\omega _0}\omega _1=-2$ and the claim is obvious. When $N=2$, the claim follows again by differentiating the expressions for $\rho _2$ and $\omega _2$ in (1.28), keeping in mind that $y_*= \frac{1}{\omega _0}>\frac{3}{2}$ and $\alpha <2$. Let now $N\ge 3$. We note that by (2.47)–(2.48)

2.93 $$\begin{aligned} \partial _{\omega _0}\rho _N =&- \frac{N}{\omega _0^2\left( N(1-\frac{1}{\omega _0})+1\right) } \rho _N + \frac{1}{2\left( N(1-\frac{1}{\omega _0})+1\right) } \partial _{\omega _0}{\mathcal {F}}_N \end{aligned}$$

2.94 $$\begin{aligned} \partial _{\omega _0}\omega _N&= - \frac{1}{2N\omega _0^2(1-\frac{1}{\omega _0})^2} {\mathcal {G}}_N - \frac{2N\left( 1-\frac{1}{\omega _0}\right) +1}{2N\omega _0^2 \left( 1-\frac{1}{\omega _0}\right) ^2\left( N(1-\frac{1}{\omega _0})+1\right) ^2}{\mathcal {F}}_N \nonumber \\&\ \ + \frac{1}{2N(1-\frac{1}{\omega _0})} \partial _{\omega _0}{\mathcal {G}}_N + \frac{1}{2N(1-\frac{1}{\omega _0})\left( N(1-\frac{1}{\omega _0})+1\right) } \partial _{\omega _0}{\mathcal {F}}_N. \end{aligned}$$

From (2.93) and Lemma 2.8 we immediately have the bound

$$\begin{aligned} \left| \partial _{\omega _0}\rho _N \right|&\lesssim \frac{1}{\omega _0^2(\frac{2}{3} -\omega _0)} |\rho _N| + \frac{1}{N\left( \frac{2}{3}-\omega _0\right) } \left| \partial _{\omega _0}{\mathcal {F}}_N\right| \\&\le \frac{C^{N-\alpha }}{N^2\omega _0^2(\frac{2}{3}-\omega _0)} + \frac{1}{N\left( \frac{2}{3}-\omega _0\right) }\left| \partial _{\omega _0}{\mathcal {F}}_N\right| \end{aligned}$$

Similarly, from (2.94) and Lemma 2.8 we obtain

$$\begin{aligned} \left| \partial _{\omega _0}\omega _N \right|&\lesssim \frac{1}{N} \left| {\mathcal {G}}_N\right| + \frac{1}{N^2(\frac{2}{3}-\omega _0)}\left| {\mathcal {F}}_N\right| + \frac{1}{N^2(\frac{2}{3}-\omega _0)}\left| \partial _{\omega _0}{\mathcal {F}}_N\right| + \frac{1}{N}\left| \partial _{\omega _0}{\mathcal {G}}_N\right| \\&\le \frac{{{\tilde{c}}}}{C^{\alpha -1}} \frac{C^{N-\alpha }}{N^2} + \frac{{{\tilde{c}}}}{C^{\alpha -1}} \frac{C^{N-\alpha }}{N^3} + \frac{1}{N^2(\frac{2}{3}-\omega _0)}\left| \partial _{\omega _0}{\mathcal {F}}_N\right| + \frac{1}{N}\left| \partial _{\omega _0}{\mathcal {G}}_N\right| \end{aligned}$$

We now recall that ${\mathcal {F}}_N$ and ${\mathcal {G}}_N$ are cubic polynomials in 2*N* variables $\rho _0,\omega _0,\dots , \rho _{N-1},\omega _{N-1}$. When we differentiate with respect to $\omega _0$, at most one term, indexed by $\rho _k$ or $\omega _k$, $0\le k\le N-1$ is differentiated. In particular, the same combinatorial structure in the problem is maintained and the same inductive proof relying on the already established bounds (2.86)–(2.87) gives (2.91)–(2.92). The remaining conclusions now follow easily. $\quad \square$

### Existence, uniqueness, and regularity near the origin

#### Theorem 2.12

Let $\rho _0>0$ be given. There exists an $0<{{\tilde{r}}}<1$ such that the formal power series

$$\begin{aligned} \rho _-(z,\rho _0):=\sum _{N=0}^\infty {{\tilde{\rho }}}_N z^N, \ \ \omega _-(z;\rho _0):= \sum _{N=0}^\infty {{\tilde{\omega }}}_N z^N \end{aligned}$$

converge for all *z* such that $0\le z<{{\tilde{r}}}$. In particular, functions $\rho _-(\cdot ;\rho _0)$ and $\omega _-(\cdot ;\rho _0)$ are real analytic on $[0,{{\tilde{r}}})$. We can differentiate the infinite sums term by term and the functions $\rho _-(\cdot ;\rho _0)$ and $\omega _-(\cdot ;\rho _0)$ solve (1.23)–(1.24) with the initial conditions $\omega _-(0;\rho _0)=\frac{1}{3}$ and $\rho _-(0;\rho _0)=\rho _0$.

#### Proof

By analogy to the previous section, we must Taylor-expand the solution at the origin $z=0$ in order to prove a local existence theorem starting from the left. An immediate consistency condition follows from the presence of $\frac{1-3\omega }{z}$ on the right-hand side of (1.24). Namely, in order to have a well-posed problem with initial data prescribed at $z=0$ we must have $\omega (0)=\frac{1}{3}$.

Assume that locally around $z=0$

2.95 $$\begin{aligned} \rho&= \sum _{N=0}^\infty {{\tilde{\rho }}}_N z^N, \ \ \omega = \sum _{N=0}^\infty {{\tilde{\omega }}}_N z^N \end{aligned}$$

Our starting point are the equations

2.96 $$\begin{aligned} \omega ' (1-y_*^2 z^2\omega ^2) - \left( 1-3\omega \right) (1-y_*^2z^2\omega ^2)\frac{1}{z} - 2y_*^2z\omega ^2(\rho -\omega )&=0, \end{aligned}$$

2.97 $$\begin{aligned} \rho ' (1-y_*^2z^2\omega ^2) +2y_*^2 z \omega \rho (\rho -\omega )&=0, \end{aligned}$$

We plug in (2.95) into (2.97) and obtain

$$\begin{aligned} 0&= \left( \sum _{k=0}^\infty k {{\tilde{\rho }}}_k z^{k-1}\right) \left( 1 - y_*^2\sum _{\ell =0}^\infty (\omega ^2)_\ell z^{\ell +2}\right) + 2 y_*^2 \sum _{k=0}^\infty \left( \omega \rho (\rho -\omega )\right) _k z^{k+1} \\&= \sum _{N=0}^\infty (N+1){{\tilde{\rho }}}_{N+1}z^N - y_*^2 \sum _{N=0}^\infty \sum _{k+\ell = N-2}(k+1){{\tilde{\rho }}}_{k+1}(\omega ^2)_\ell z^N + 2 y_*^2 \sum _{N=0}^\infty \left( \omega \rho (\rho -\omega )\right) _{N-1} z^N, \end{aligned}$$

where, by definition ${{\tilde{\rho }}}_k={{\tilde{\omega }}}_k=0$ for $k<0$. Equating the coefficients above, we conclude that for any non-negative *N* we have

2.98 $$\begin{aligned}&(N+1){{\tilde{\rho }}}_{N+1} - y_*^2 \sum _{k+\ell = N-2}(k+1){{\tilde{\rho }}}_{k+1}(\omega ^2)_\ell + 2 y_*^2 \left( \omega \rho (\rho -\omega )\right) _{N-1} = 0, \end{aligned}$$

which is precisely (2.33). Similarly, after plugging in (2.95) into (2.96) we obtain

$$\begin{aligned} 0&= \left( \sum _{k=0}^\infty k {{\tilde{\omega }}}_k z^{k-1}\right) \left( 1 - y_*^2\sum _{\ell =0}^\infty (\omega ^2)_\ell z^{\ell +2}\right) + 3 \sum _{k=0}^\infty {{\tilde{\omega }}}_{k+1}z^k\left( 1 - y_*^2\sum _{\ell =0}^\infty (\omega ^2)_\ell z^{\ell +2}\right) \\&\ \ \ \ - 2 y_*^2 \sum _{k=0}^\infty \left( \omega ^2(\rho -\omega )\right) _k z^{k+1} \\&= \sum _{N=0}^\infty (N+1){{\tilde{\omega }}}_{N+1}z^N - y_*^2 \sum _{N=0}^\infty \sum _{k+\ell = N-2}(k+1){{\tilde{\omega }}}_{k+1}(\omega ^2)_\ell z^N + 3 \sum _{N=0}^\infty {{\tilde{\omega }}}_{N+1}z^N \\&\ \ \ \ - 3 y_*^2 \sum _{N=0}^\infty \sum _{k+\ell = N-2} {{\tilde{\omega }}}_{k+1}(\omega ^2)_\ell z^N - 2 y_*^2 \sum _{N=0}^\infty \left( \omega ^2(\rho -\omega )\right) _{N-1} z^N. \end{aligned}$$

Therefore for any $N\ge 0$ we have

2.99 $$\begin{aligned} (N+4){{\tilde{\omega }}}_{N+1} - y_*^2 \sum _{k+\ell = N-2}(k+1){{\tilde{\omega }}}_{k+1}(\omega ^2)_\ell - 3 y_*^2\sum _{k+\ell = N-2} {{\tilde{\omega }}}_{k+1}(\omega ^2)_\ell - 2 y_*^2 \left( \omega ^2(\rho -\omega )\right) _{N-1} =0 \end{aligned}$$

It is clear from (2.98) that $\rho _0=\rho (0)$ is a free parameter. Identities (2.98)–(2.99) give the recursive relationships

2.100 $$\begin{aligned} {{\tilde{\rho }}}_{N+1}&= \frac{1}{N+1} \tilde{{\mathcal {F}}}_{N+1}, \ \ N\ge 0 \end{aligned}$$

2.101 $$\begin{aligned} {{\tilde{\omega }}}_{N+1}&= \frac{1}{N+4} \tilde{{\mathcal {G}}}_{N+1}, \ \ N\ge 0. \end{aligned}$$

where

2.102 $$\begin{aligned} \tilde{{\mathcal {F}}}_{N+1}&: = y_*^2 \sum _{k+\ell = N-2}(k+1){{\tilde{\rho }}}_{k+1}(\omega ^2)_\ell - 2 y_*^2 \left( \omega \rho (\rho -\omega )\right) _{N-1} \end{aligned}$$

2.103 $$\begin{aligned} \tilde{{\mathcal {G}}}_{N+1}&: =y_*^2 \sum _{k+\ell = N-2}(k+1){{\tilde{\omega }}}_{k+1}(\omega ^2)_\ell +3 y_*^2\sum _{k+\ell = N-2} {{\tilde{\omega }}}_{k+1}(\omega ^2)_\ell - 2 y_*^2 \left( \omega ^2(\rho -\omega )\right) _{N-1} \end{aligned}$$

The rest of the proof is now entirely analogous to the proof of Theorem 2.10 and we leave out the details. $\square$

#### Remark 2.13

Letting $N=0$ in (2.100)–(2.101) we immediately see from (2.102)–(2.103) that $\tilde{{\mathcal {F}}}_{1}=\tilde{{\mathcal {G}}}_{1}=0$ and therefore ${{\tilde{\rho }}}_1={{\tilde{\omega }}}_1=0$. Letting $N=1$ in (2.98)–(2.99) we obtain

2.104 $$\begin{aligned} {{\tilde{\rho }}}_2&= - y_*^2 \frac{1}{3}\rho _0(\rho _0-\frac{1}{3}) = - \frac{1}{3} y_*^2 \rho _0^2 + \frac{1}{9} y_*^2 \rho _0 \nonumber \\ {{\tilde{\omega }}}_2&= \frac{2}{45} y_*^2(\rho _0-\frac{1}{3}) = - \frac{2y_*^2}{135}+ \frac{2y_*^2}{45} \rho _0 \end{aligned}$$

By (2.104) we have in the vicinity of $z=0$

$$\begin{aligned} \partial _{\rho _0} \omega _-(z;\rho _0) = \frac{2y_*^2}{45} z^2 + \sum _{N=3}^\infty {{\tilde{\omega }}}_Nz^N >0, \ \ 0<z<{{\tilde{r}}}. \end{aligned}$$

## The Outer Region $z>1$

.

In this section we show that for any value of $y_*\in [2,3]$ there exists a unique LP-type solution in the exterior region. Such a statement is true because the flow “moves” in a stable direction as $z\rightarrow \infty$. This should be contrasted to the more delicate analysis of the flow in the inner region. Our first preparatory lemma lists a number of simple properties in the vicinity of $z=1$, which follow by continuity and careful use of the LP condition (1.28).

### Lemma 3.1

(Initialisation). Let $y_*\in [2,3]$ and let $(\rho (\cdot ),\omega (\cdot )):=(\rho (\cdot ;y_*),\omega (\cdot ;y_*))$ be the unique local LP-type solution to (1.23)–(1.24) given by Theorem 2.10. Then there exists a $\delta >0$ such that the following bounds hold:

3.105 $$\begin{aligned} \rho '(z)&<0, \ \ z\in (1,1+\delta ), \end{aligned}$$

3.106 $$\begin{aligned} \omega '(z)&>0, \ \ z\in (1, 1+\delta ) \end{aligned}$$

3.107 $$\begin{aligned} \frac{1}{3}< \omega (z)&< 1, \ \ z\in (1, 1+\delta ) \end{aligned}$$

3.108 $$\begin{aligned} -2\frac{\rho (z)}{z}<\rho '(z)&<-\frac{\rho (z)}{ z}, \ \ z\in (1, 1+\delta ) \end{aligned}$$

3.109 $$\begin{aligned} \omega (z)&>\frac{1}{y_*z}, \ \ z\in (1, 1+\delta ) \end{aligned}$$

3.110 $$\begin{aligned} \frac{1}{y_*z}&> \rho (z), \ \ \ \ z\in (1, 1+\delta ) \end{aligned}$$

3.111 $$\begin{aligned} \rho (z) \omega (z)&>(y_*z)^{-2}, \ \ z\in (1, 1+\delta ) \end{aligned}$$

### Proof

Claims (3.105)–(3.107) are clear and follow by a continuity argument from (1.28). To prove claim (3.108) we first note that due to $\rho '(1)=-\frac{1}{y_*}$ we have $\rho '(z)+\frac{\rho (z)}{z}=0$ at $z=1$. Notice that for any solutions of LP-type by (1.28)

2.112 $$\begin{aligned} 2\rho _2y_*-2 = - \frac{y_*^2-2y_*+1}{(2y_*-3)} <0, \end{aligned}$$

where we recall $\rho _2=\frac{1}{2}\rho ''(1)$ by (1.27). Since $\frac{d}{dz}\left( \rho '(z)+\frac{\rho (z)}{z}\right) \Big |_{z=1}= \rho ''(z)-\frac{\rho (z)}{z^2}+\frac{\rho '(z)}{z}\Big |_{z=1} =2\rho _2 - 2 \frac{1}{y_*}<0$ by (2.112), claim follows by a continuity argument. Claim (3.109) follows since $\omega (1)=\frac{1}{y_*}$ and $\omega$ is by (3.106) locally strictly increasing and $\frac{1}{y_*z}$ is clearly strictly decreasing. To prove (3.110), consider

$$\begin{aligned} F(z): = \frac{1}{y_*z} - \rho (z). \end{aligned}$$

Note that $F(1)=F'(1)=0$ and it is therefore necessary to evaluate the second derivative of *F*. A direct calculation shows that $F''(1) = \frac{2}{y_*}-2\rho _2$ which is strictly positive by  (2.112). Thus *F* is strictly increasing on $(1, 1+\delta )$ for a sufficiently small $\delta$.

Finally, from (1.23)–(1.24) we obtain the equation

2.113 $$\begin{aligned} \left( \rho \omega z^2\right) ' = (1-\omega ) \rho z. \end{aligned}$$

By (3.107) we conclude that $\rho \omega z^2$ is strictly increasing on the interval $(1-\delta , 1+\delta )$ and therefore, since $\rho \omega =y_*^{-2}$ at $z=1$, claim (3.111) follows. $\quad \square$

The next lemma shows the crucial dynamic trapping property.

### Lemma 3.2

(Invariant set). Let $y_*\in [2,3]$ and let $(\rho (\cdot ;y_*),\omega (\cdot ;y_*))$ be the unique local LP-type solution to (1.23)–(1.24) given by Theorem 2.10. Let $I = (1, T)$ be the maximal interval of existence to the right of $z=1$ on which the properties

2.114 $$\begin{aligned} \frac{1}{y_*z}&> \rho (z), \end{aligned}$$

2.115 $$\begin{aligned} \rho (z)\omega (z)&>(y_*z)^{-2}, \end{aligned}$$

hold. Note that $T\ge 1+\delta >1$ by Lemma 3.1. Then the following bounds hold:

2.116 $$\begin{aligned} \frac{1}{3}&< \omega (z) < 1, \ \ z\in (1, T) \end{aligned}$$

2.117 $$\begin{aligned} -2\frac{\rho (z)}{ z}&<\rho '(z)<-\frac{\rho (z)}{ z}, \ \ z\in (1, T) \end{aligned}$$

### Proof

By (2.114)–(2.115) we have $\omega >\rho$ on (1, *T*) and

2.118 $$\begin{aligned} \omega (z) >\frac{1}{y_*z} \ \ z\in (1, T). \end{aligned}$$

Therefore from (1.23) $\rho '<0$ on (1, *T*).

**Proof of** (2.116). We note that on (1, *T*) due to $\omega >\rho$ and $\omega (z) >\frac{1}{y_*z}$ we have from (1.24) $\omega '(z)>\frac{1-3\omega (z)}{z}.$ Integrating over [1, *z*] for any $z\in (1,T)$ we conclude

$$\begin{aligned} \omega (z) > (\frac{1}{y_*}-\frac{1}{3}) z^{-3} + \frac{1}{3} \ge \frac{1}{3}, \ \ z\in (1,T). \end{aligned}$$

We may rewrite (1.24) in the form

2.119 $$\begin{aligned} \omega ' = \frac{1-\omega }{z} + \frac{-2\omega +2y_*^2z^2\omega ^2\rho }{z\left( 1-y_*^2z^2\omega ^2 \right) }. \end{aligned}$$

From (2.115) we have on (1, *T*) $-2\omega +2y_*^2z^2\omega ^2\rho >0$ and therefore from (2.119) (together with $\omega (z) y_*z >1$) we conclude that $\omega '(z)<\frac{1-\omega (z)}{z}.$ Integrating over [1, *z*] for any $z\in (1,T)$ we conclude from $y_*>1$

$$\begin{aligned} \omega (z)< (\frac{1}{y_*}-1) z^{-1} + 1 < 1, \ \ z\in (1,T). \end{aligned}$$

Therefore (2.116) holds.

**Proof of** (2.117). We may rewrite (1.23) in the form

2.120 $$\begin{aligned} \frac{\rho 'z}{\rho } = -2 +2 \frac{1-y_*^2 z^2\omega \rho }{1-y_*^2 z^2\omega ^2}. \end{aligned}$$

Since by (2.115) $1-y_*^2 z^2 \omega \rho <0$ the lower bound follows immediately. To prove the upper bound we rewrite (1.23) in the form

2.121 $$\begin{aligned} \frac{\rho 'z}{\rho } = -1 + \frac{1+y_*^2z^2 \omega ^2-2y_*^2z^2 \omega \rho }{1-y_*^2z^2 \omega ^2} = -1 + \frac{(1-y_*z\omega )^2+2y_*z \omega (1-y_*z \rho )}{1-y_*^2 z^2 \omega ^2}. \end{aligned}$$

The last expression is strictly less than $-1$ by (2.118) and (2.114). $\quad \square$

Finally, combining the previous two lemmas we obtain the desired forward global existence result in the outer region $z\ge 1$.

### Proposition 3.3

(Forward global existence). Let $y_*\in [2,3]$ and let $(\rho (\cdot ;y_*),\omega (\cdot ;y_*))$ be the unique local LP-type solution to (1.23)–(1.24) given by Theorem 2.10. Then there exists a unique forward global solution to (1.23)–(1.24) on $[1,\infty )$ satisfying the following properties:

2.122 $$\begin{aligned} \frac{1}{3}&<\omega (z)<1 \end{aligned}$$

2.123 $$\begin{aligned} -2&< \frac{\rho '(z) z}{\rho (z)} <-1, \end{aligned}$$

Moreover,

2.124 $$\begin{aligned} \rho (z)= \frac{C}{z^2} \left( 1+ O_{z\rightarrow \infty }(\frac{1}{z})\right) , \ \ \omega (z)=1+ O_{z\rightarrow \infty }\left( \frac{1}{z}\right) \end{aligned}$$

### Proof

Let *T* be defined as in Lemma 3.2 and assume that $T<\infty$. Notice that by the bounds in Lemma 3.2, both $\omega$ and $\rho$ remain bounded and away from the sonic point singularity for $z\in (1,T)$. At *T* we must have either $\frac{1}{y_*T} =\rho (T)$ or $\rho (T)\omega (T) = \frac{1}{y_*^2 T^2}$.

Let $\frac{1}{y_*T} =\rho (T)$. Since $\left( \rho y_*z\right) '=\rho y_*(1+\frac{\rho 'z}{\rho })<0$ by (2.117) for all $z\in (1,T)$, we must have $\rho (T)y_*T<\rho (1) y_*= 1$, a contradiction.

Let now $\rho (T)\omega (T) = \frac{1}{y_*^2 T^2}$. Since $\omega <1$ on (1, *T*) we conclude from (2.113) that $z^2 \rho (z)\omega (z)$ is strictly increasing on (1, *T*). Therefore

2.125 $$\begin{aligned} T^2\rho \omega > y_*^2 \rho (1)\omega (1) =1 \end{aligned}$$

a contradiction. Therefore, the solution $(\rho (\cdot ;y_*),\omega (\cdot ;y_*))$ exists for all $z>1$. Finally, since $\omega (z)>\frac{1}{3}$ on $(1,\infty )$ and $z\rho \le \frac{1}{y_*}$ by the above bounds, we conclude easily that $\left| \frac{1-y_*^2 z^2\omega \rho }{1-y_*^2 z^2\omega ^2}\right| \lesssim \frac{1}{z}$, $z>1$. It follows from (2.120) that $\frac{\rho 'z}{\rho } = -2 + O(\frac{1}{z})$ and this implies the $\rho$-asymptotics in (2.124). From (1.23)–(1.24) it is easy to see that $(\omega z)' = 1-\frac{\rho '}{\rho }z\omega -2\omega = 1+ \omega O(\frac{1}{z^2})$, where in the last equality we have used the $\rho$-asymptotics (2.124) and (2.120). This easily gives the $\omega$-asymptotics in (2.120). $\quad \square$

## The Inner Region $0\le z<1$

By Theorem 2.10 and Lemma 2.11 there exists an $r>0$ such that for any $y_*\in [2,3]$ there is unique LP-type solution $(\rho (z;y_*),\omega (z;y_*))$ on $(1-r,1+r)$, which is analytic-in-*z* and uniformly continuous with respect to $y_*$. The next lemma records the obvious statement that one can extend the existence interval as long as we are away from the sonic line.

### Lemma 4.1

(Local existence and uniqueness away from the sonic line). Let $y_*\in [2,3]$ be given and assume that for some $z\in (0,1)$ the conditions

$$\begin{aligned} 1 - z^2y_*^2\omega (z)^2>0, \ \ \rho (z)>0 \end{aligned}$$

hold. Then there exists a unique smooth local-in-*z* solution to the initial value problem (1.23)–(1.24) on some time interval $(z-T,z+T)\subset (0,1)$.

### Proof

The proof is a standard consequence of the local well-posedness theory for ordinary differential equations. $\quad \square$

To every $y_*\in [2,3]$ (i.e. $\omega _0=\omega (1)\in [\frac{1}{3},\frac{1}{2}]$) we associate the *sonic* time of existence to the left

4.126 $$\begin{aligned} s(y_*): = \inf \left\{ z\in (0,1)\,\big | \text { solution exists on} (z,1]\text { and } \omega ^2(z;y_*)z^2y_*^2<1\right\} . \end{aligned}$$

Clearly $0\le s(y_*)< 1-r$. By Lemma 4.1 the solution can be continued to the left starting at $z=1-r$ for a short time and the maximal time of existence to the left is smaller or equal to $s(y_*)$. Sonic time is of central importance in our analysis and our first goal is to show that there exists a $y_*\in [2,3]$ such that $s(y_*)=0$. To that end we prove a number of preparatory lemmas. We next collect important a priori bounds that hold on $(s(y_*),1)$.

### Lemma 4.2

Let $y_*\in [2,3]$ be given and consider the unique LP-type solution $(\rho (\cdot ;y_*), \omega (\cdot ;y_*))$ to the left of $z=1$. For any $z\in (s(y_*),1)$ we have the a priori bounds

4.127 $$\begin{aligned} 0< \rho (z)&<\frac{1}{y_*z}, \end{aligned}$$

4.128 $$\begin{aligned} |\omega (z)|&<\frac{1}{y_*z}, \end{aligned}$$

4.129 $$\begin{aligned} (z\rho (z))'&>0. \end{aligned}$$

### Proof

Let $\mathring{z}\in (s(y_*),1)$ be given. From the definition (4.126) it follows that there exists an $\eta >0$ such that $\omega (z)^2z^2y_*^2<1-\eta$ for all $z\in [\mathring{z},1-r]$ and in particular

4.130 $$\begin{aligned} \omega (z)^2 \le \frac{1-\eta }{y_*^2\mathring{z}^2}=:C_\eta , \ \ z\in [\mathring{z},1-r]. \end{aligned}$$

We first show that $\rho$ remains positive on $(s(y_*),1)$. Let ${\bar{z}}:=\inf _{z\in [\mathring{z},1]}\left\{ \rho (\zeta )>0 \ \text {for}\right. \left. \text {all} \ \zeta \in (z,1]\right\}$. By Theorem 2.10 we have ${\bar{z}} < 1-r$. Since $\rho >0$ on $({\bar{z}},1]$ Eq. (1.23) gives

$$\begin{aligned} \left( \log \rho \right) ' = -\frac{2y_*^2 z \omega }{1-y_*^2 z^2\omega ^2}(\rho -\omega ), \ \ z\in ({\bar{z}},1]. \end{aligned}$$

Therefore for any $z\in ({\bar{z}},1-r]$ we get

$$\begin{aligned} \rho (z)&=\rho (1-r) \exp \left( \int _{z}^{1-r} \frac{2y_*^2 \zeta \omega }{1-y_*^2 \zeta ^2\omega ^2}(\rho -\omega )\,d\zeta \right) \\&\ge \rho (1-r) \exp \left( - \frac{2 y_*^2 C_\eta ^2}{\eta }\right) \exp \left( - \frac{2 y_*^2 C_\eta }{\eta } \int _z^{1-r} \rho (\zeta )\,d\zeta \right) . \end{aligned}$$

The right-hand side is strictly positive and as $z\rightarrow {\bar{z}}$ it clearly remains strictly positive. Therefore ${\bar{z}} = \mathring{z}$.

In order to prove the upper bound for $\rho$, we consider

$$\begin{aligned} f(z) : = 1 - y_*z \rho (z). \end{aligned}$$

Using (1.23) it is checked that

4.131 $$\begin{aligned} f'(z) + f(z) \frac{2zy_*^2\omega \rho }{1-y_*^2z^2\omega ^2} = - \frac{y_*(1-y_*z \omega )^2 \rho }{1-y_*^2z^2\omega ^2} \end{aligned}$$

By the LP-type sonic conditions  (1.25) and (1.28) it is easy to see that $f(1)=f'(1)=0$. To determine the sign of *f* near $z=1$ it is therefore necessary to compute the second derivative of *f*. Since

$$\begin{aligned} f''(z)\big |_{z=1} = -2y_*\rho '(1) - y_*\rho ''(1) = 2-\frac{-y_*^2+6y_*-7}{ 2y_*-3} = \frac{y_*^2-2y_*+1}{2y_*-3}, \end{aligned}$$

we conclude that $f>0$ locally around 1 as the above expression is strictly positive for $y_*\in [2,3]$. In fact, by choosing a possibly smaller *r*, we may assume $f(z)>0$ for all $z\in [1-r,1)$. We let ${{\tilde{z}}}:=\inf _{z\in [\mathring{z},1]}\left\{ f(\zeta )>0 \ \text {for all} \ \zeta \in (z,1]\right\}$. Since $\rho >0$ on $[\mathring{z},1]$ it follows that the right-hand side of (4.131) is negative for any $z\in ({{\tilde{z}}}, 1]$. Integrating (4.131) for any $z\in [{{\tilde{z}}}, 1-r]$ we get

$$\begin{aligned} f(z) \ge f(1-r) \exp \left( \int _{z}^{1-r} \frac{2\zeta y_*^2\omega \rho }{1-y_*^2\zeta ^2\omega ^2}\,d\zeta \right) . \end{aligned}$$

By an analogous argument as above, we conclude $f({{\tilde{z}}})>0$ and therefore ${{\tilde{z}}} = \mathring{z}$. Therefore $f(z)>0$ on $(s(y_*),1)$ as claimed. From (4.131) we then conclude $f'(z)<0$ which is equivalent to (4.129). $\square$

The following lemma shows that solutions which are a finite distance $\eta$ away from the sonic line and defined for all $z\ge {\bar{z}}>0$, can be extended to the left by a finite time depending only on $\eta$ and ${\bar{z}}$.

### Lemma 4.3

Let $y_*\in [2,3]$ be given and consider the unique LP-type solution $(\rho (\cdot ;y_*), \omega (\cdot ;y_*))$ to the left of $z=1$, given by Theorem 2.10. Assume that for some ${\bar{z}}\in (0,1-r)$ and $\eta >0$ we have ${\bar{z}}>s(y_*)$ and the conditions

4.132 $$\begin{aligned} 1 - z^2y_*^2\omega (z;y_*)^2>\eta , \ \ \rho (z)>0, \ \ z\in [{\bar{z}},1-r], \end{aligned}$$

hold. Then there exists a $t=t(\eta ,{\bar{z}})>0$ such that the solution can be continued to the interval $[{\bar{z}}- t,1)$ so that

$$\begin{aligned} 1 - z^2y_*^2\omega (z;y_*)^2>0, \ \ \rho (z)>0, \ \ z\in [{\bar{z}}- t,1-r]. \end{aligned}$$

### Proof

By Lemma 4.2 the following a priori bounds hold:

4.133 $$\begin{aligned} \Vert \omega \Vert _{C^0([{\bar{z}},1-r])}&< \frac{1}{y_*{\bar{z}}}, \end{aligned}$$

4.134 $$\begin{aligned} \Vert \rho \Vert _{C^0([{\bar{z}},1-r])}&< \frac{1}{y_*{\bar{z}}}. \end{aligned}$$

Formally, for any $0<z\le {\bar{z}}$ we write the Eqs. (1.23)–(1.24) in their integral form

4.135 $$\begin{aligned} \omega (z)&= \omega ({\bar{z}}) + \int _{z}^{{\bar{z}}} \frac{3\omega -1}{\tau }\,d\tau - 2y_*^2\int _{z}^{{\bar{z}}} {\mathcal {F}}(y_*,\rho ,\omega )(\tau )\, d\tau , \end{aligned}$$

4.136 $$\begin{aligned} \rho (z)&=\rho ({\bar{z}}) + 2y_*^2\int _{z}^{{\bar{z}}} {\mathcal {G}}(y_*,\rho ,\omega )(\tau )\, d\tau , \end{aligned}$$

where

4.137 $$\begin{aligned} {\mathcal {F}}(y_*,\rho ,\omega )(z)&: = \frac{z \omega ^2(\rho -\omega )}{1-y_*^2 z^2\omega ^2}, \end{aligned}$$

4.138 $$\begin{aligned} {\mathcal {G}}(y_*,\rho ,\omega )(z)&: = \frac{z \omega \rho (\rho -\omega )}{1-y_*^2 z^2\omega ^2}. \end{aligned}$$

This motivates the following Picard iteration, where we let

4.139 $$\begin{aligned} \rho _n(z)&=\rho ({\bar{z}}) + 2y_*^2\int _{z}^{{\bar{z}}} {\mathcal {G}}(y_*,\rho _{n-1},\omega _{n-1})(\tau )\, d\tau . \end{aligned}$$

4.140 $$\begin{aligned} \omega _n(z)&= \omega ({\bar{z}}) + \int _{z}^{{\bar{z}}} \frac{3\omega _{n-1}-1}{\tau }\,d\tau - 2y_*^2\int _{z}^{{\bar{z}}} {\mathcal {F}}(y_*,\rho _{n-1},\omega _{n-1})(\tau )\, d\tau . \end{aligned}$$

For an $M>1$ sufficiently large and $t = t({\bar{z}},\eta )<\frac{{\bar{z}}}{2}$ sufficiently small (both to be specified below), we make the inductive assumptions

4.141 $$\begin{aligned} \left| \omega _k(z) \right|&\le \frac{4}{y_*{\bar{z}}}, \ \ z \in [{\bar{z}}- t, {\bar{z}}], \ \ k=0,1,2,\dots , n-1, \end{aligned}$$

4.142 $$\begin{aligned} \left| \rho _k(z) \right|&\le M, \ \ z\in [{\bar{z}}-t, {\bar{z}}], \ \ k =0,1,2,\dots , n-1, \end{aligned}$$

4.143 $$\begin{aligned} 1 - z^2y_*^2\omega _k^2(z)&\ge \frac{\eta }{2}, \ \ z\in [{\bar{z}}- t, {\bar{z}}], \ \ k=0,1,2,\dots , n-1. \end{aligned}$$

Here we choose to start the iteration with constant functions $(\rho _0(z),\omega _0(z))\equiv (\rho ({\bar{z}}),\omega ({\bar{z}}))$, $z\in [{\bar{z}}-t,{\bar{z}}]$ so that it satisfies the inductive assumptions. From (4.139), we easily conclude

$$\begin{aligned} \left| \rho _n(z)\right| \le \left| \rho ({\bar{z}})\right| + \frac{CM^2}{{\bar{z}}^2 \eta } |z-{\bar{z}}|, \ \ z\in [{\bar{z}}-t, {\bar{z}}], \end{aligned}$$

and therefore, for *t* sufficiently small and a sufficiently large *M* (but from now on fixed), we obtain the bound (4.142) for $k=n$. From (4.140) we easily conclude

4.144 $$\begin{aligned} \left| \omega _n(z)\right| \le \left| \omega ({\bar{z}})\right| + C \left( 1+ 3|\omega _{n-1}|_{C^0}\right) \frac{|z-{\bar{z}}|}{{\bar{z}}}+ \frac{CM}{{\bar{z}}^3 \eta } |z-{\bar{z}}|, \ \ z\in [{\bar{z}}-t, {\bar{z}}],\quad \end{aligned}$$

and therefore, for $t=t(\eta ,{\bar{z}})$ sufficiently small we obtain the bound (4.141) for $k=n$ using (4.133). We also observe that for any $z\in [{\bar{z}}- t,{\bar{z}}]$

4.145 $$\begin{aligned} 1-y_*^2z^2\omega _n^2&= 1-y_*^2z^2\omega _0^2 + y_*^2z^2 \sum _{k=1}^{n} \left( \omega _{k-1}^2-\omega _k^2\right) \nonumber \\&\ge 1-y_*^2z^2\omega _0^2 - C{\bar{z}} \sum _{k=1}^{n}|\omega _k-\omega _{k-1}| \end{aligned}$$

Subtracting two iterates $(\omega _n,\rho _n)$ and $(\omega _{n-1},\rho _{n-1})$ we conclude

4.146 $$\begin{aligned} \omega _n(z)-\omega _{n-1}(z)&=3\int _{z}^{{\bar{z}}} \frac{\omega _n-\omega _{n-1}}{\tau }\,d\tau - 2y_*^2\int _{z}^{{\bar{z}}} \left( {\mathcal {F}}(y_*,\rho _{n-1},\omega _{n-1})\right. \nonumber \\&\left. -{\mathcal {F}} (y_*,\rho _{n-2},\omega _{n-2})\right) \,d\tau , \end{aligned}$$

4.147 $$\begin{aligned} \rho _n(z)-\rho _{n-1}(z)&= 2y_*^2\int _{z}^{{\bar{z}}} \left( {\mathcal {G}}(y_*,\rho _{n-1},\omega _{n-1})-{\mathcal {G}} (y_*,\rho _{n-2},\omega _{n-2})\right) \,d \tau , \end{aligned}$$

A simple algebraic manipulation and the bounds (4.132), (4.133), and (4.134) imply that there exists a constant ${{\tilde{C}}}={{\tilde{C}}}(M,{\bar{z}})$ such that for all $1\le k\le n-1$ and $z\in [{\bar{z}}- t, {\bar{z}}]$

4.148 $$\begin{aligned} \left| {\mathcal {F}}(y_*,\rho _k,\omega _k) - {\mathcal {F}}(y_*,\rho _{k-1}, \omega _{k-1}) \right|&\le \frac{{{\tilde{C}}}}{\eta ^2} \left( |\omega _{k}-\omega _{k-1}|+|\rho _{k}-\rho _{k-1}| \right) , \end{aligned}$$

4.149 $$\begin{aligned} \left| {\mathcal {G}}(y_*,\rho _k,\omega _k) - {\mathcal {G}}(y_*,\rho _{k-1}, \omega _{k-1}) \right|&\le \frac{{{\tilde{C}}}}{\eta ^2}\left( |\omega _{k}-\omega _{k-1}| +|\rho _{k}-\rho _{k-1}| \right) . \end{aligned}$$

Allowing the constants *C* to change from line to line, but to possibly depend on ${\bar{z}}, \eta$, we plug (4.148)–(4.149) back into (4.146)–(4.147) and using ${\bar{z}}-t\ge \frac{{\bar{z}}}{2}$ we obtain for $k=1,2,\dots n$

$$\begin{aligned} u_k(z)&\le C u_{k-1}(z) |z-{\bar{z}}|, \\ u_k(z)&: = \left| \omega _n(z)-\omega _{n-1}(z) \right| _{C^0([z,{\bar{z}}])} + \left| \rho _n(z)-\rho _{n-1}(z) \right| _{C^0([z,{\bar{z}}])}. \end{aligned}$$

Choosing $t=t(\eta ,{\bar{z}})$ sufficiently small we conclude that there exists a constant $0<c<1$ such that $u_k\le c u_{k-1}$ for all $k=1,2,\dots , n$. By (4.144) and (4.145)

4.150 $$\begin{aligned} 1-y_*^2z^2\omega _n^2> \eta - C{\bar{z}}\sum _{k=1}^{n} c^k > \frac{\eta }{2}, \ \ z\ge {\bar{z}}- t, \end{aligned}$$

for $t=t(\eta ,{\bar{z}})$ and therefore *c* sufficiently small. Since we can choose *t* so small that $t<\frac{{\bar{z}}}{2}$ bound (4.150) gives us (4.143) with $k=n$. By the standard arguments we pass to a limit as $n\rightarrow \infty$ and obtain the unique LP-type solution on the interval $[{\bar{z}}-t,1]$. $\quad \square$

### Lemma 4.4

(No blow up before the sonic line). Let $y_*\in [2,3]$ be given and consider the unique LP-type solution $(\rho (\cdot ;y_*),\omega (\cdot ;y_*))$ to the left of $z=1$. If $s(y_*)>0$ then

$$\begin{aligned} \lim _{z\rightarrow s(y_*)}\omega (z)^2 = \frac{1}{y_*^2s(y_*)^2}. \end{aligned}$$

### Proof

Suppose the opposite. Then there exists an $\eta >0$ such that $1-z^2y_*^2\omega (z)^2>\eta$ for all $z\in (s(y_*),1-r)$. By Lemmas 4.2–4.3 there exists a constant $t=t(\eta ,s(y_*))$ such that the solution can be continued to the interval $(s(y_*)-t,1-r)$ and stay below the sonic line. A contradiction. $\quad \square$

### Sonic time continuity properties

Using a continuity argument we next show that the sonic time function $y_*\rightarrow s(y_*)$ is upper semi-continuous.

#### Proposition 4.5

Let $y_*\in [2,3]$ be given and consider the unique LP-type solution to the problem (1.23)–(1.24) to the left of $z=1$.

(a): (Upper semi-continuity of the sonic time). Then
  $$\begin{aligned} \limsup _{{{\tilde{y}}}_*\rightarrow y_*} s({{\tilde{y}}}_*) \le s(y_*), \end{aligned}$$
  i.e. the map $y_*\rightarrow s(y_*)$ is upper semi-continuous. In particular, if $s(y_*)=0$ then the map $s(\cdot )$ is continuous at $y_*$.
(b): ([Continuity of the flow away from the sonic time]) Let $\{y^n_*\}_{n\in {\mathbb {N}}}\subset [2,3]$ and $y_*\subset [2,3]$ satisfy $\lim _{n\rightarrow \infty }y^n_*= y_*$. Let $1-r>z>\max \{s(y_*),\sup _{n\in {\mathbb {N}}}s(y_*^n)\}$. Then
  $$\begin{aligned} \lim _{n\rightarrow \infty }\omega (z;y_*^n) = \omega (z;y_*), \ \ \lim _{n\rightarrow \infty }\rho (z;y_*^n) = \rho (z;y_*). \end{aligned}$$
(c): Let $\{y^n_*\}_{n\in {\mathbb {N}}}\subset [2,3]$ and $y_*\subset [2,3]$ satisfy $\lim _{n\rightarrow \infty }y^n_*= y_*$. Assume that there exist $0<Z<1-r$ and $\eta >0$ such that $s(y_*^n)<Z$ for all $n\in {\mathbb {N}}$ and the following uniform bound holds:
  4.151 $$\begin{aligned} 1 - (y_*^n)^2 z^2 \omega (z;y_*^n)^2>\eta , \ \ n\in {\mathbb {N}}, \ \ z\in [Z,1-r]. \end{aligned}$$
  Then there exists a $T=T(\eta ,Z)>0$ such that
  4.152 $$\begin{aligned} s(y_*)<Z-T, \ \ s(y_*^n)<Z-T, \ \ n\in {\mathbb {N}}. \end{aligned}$$

#### Proof

*Proof of part (a)* For any $y_*\in [2,3]$, on the interval $(s(y_*), 1]$ by Lemma 4.2 we have the a priori bounds

4.153 $$\begin{aligned} |\rho (z;y_*)|\le \frac{1}{y_*z}\le \frac{1}{2z}, \ \ |\omega (z,y_*)| \le \frac{1}{y_*z}\le \frac{1}{2z}, \ \ y_*\in [2,3], \ z\in (s(y_*),1). \end{aligned}$$

Fix an arbitrary $\mathring{z} \in (s(y_*),1-r)$. By the definition of the sonic time $s(y_*)$, there exists an $\eta >0$ such that

4.154 $$\begin{aligned} 1 - z^2y_*^2\omega (z)^2>\eta , \ \ z\in [\mathring{z},1-r], \end{aligned}$$

where $\omega (z):=\omega (z;y_*)$. By (4.153) it is clear that there exists a constant $C = C(\mathring{z})$ such that for any ${{\tilde{y}}}_*\in [2,3]$

4.155 $$\begin{aligned} |\rho (z;{{\tilde{y}}}_*)|\le C, \ \ |\omega (z,{{\tilde{y}}}_*)| \le C, \ \ z\in [\mathring{z}, 1-r]\cap (s({{\tilde{y}}}_*), 1-r). \end{aligned}$$

Let $1>\delta >0$ be a small number to be specified later. Let $|{{\tilde{y}}}_*-y_*|<\delta$ and consider the two solutions $(\rho (z;y_*),\omega (z;y_*))$ and $(\rho (z;{{\tilde{y}}}_*), \omega (z;{{\tilde{y}}}_*))$ on the interval $(Z,1-r]$, where

$$\begin{aligned} Z : = \max \{s({{\tilde{y}}}_*), \mathring{z}\}. \end{aligned}$$

Clearly both solutions are well-defined on $( Z,1-r]$..

Our goal is to show that $Z=\mathring{z}$ if $\delta$ is sufficiently small. To that end, assume the opposite, i.e. $Z=s({{\tilde{y}}}_*)$. In the rest of the proof the constant *C* may change from line to line, but may depend only on $\mathring{z}$ and $y_*$.

For any $z\in (Z,1-r)$ integrating (1.23)–(1.24) over $[z,1-r]$ to obtain

4.156 $$\begin{aligned} \omega (z)&= \omega (1-r) + \int _{z}^{1-r} \frac{3\omega -1}{\tau }\,d\tau - 2y_*^2\int _{z}^{1-r} \frac{\tau \omega ^2(\rho -\omega )}{1-y_*^2\tau ^2\omega ^2}\, d\tau , \end{aligned}$$

4.157 $$\begin{aligned} \rho (z)&=\rho (1-r) + 2y_*^2\int _{z}^{1-r} \frac{\tau \omega \rho (\rho -\omega )}{1-y_*^2\tau ^2\omega ^2}\, d\tau . \end{aligned}$$

For any $y_*,{{\tilde{y}}}_*\in [2,3]$ denote the corresponding LP- type solutions by $(\rho ,\omega )$ and $({{\tilde{\rho }}},{{\tilde{\omega }}})$ respectively. From (4.135)–(4.136) we obtain

4.158 $$\begin{aligned} \omega (z)-{{\tilde{\omega }}}(z)&= \omega (1-r)-{{\tilde{\omega }}}(1-r)+3\int _{z}^{1-r} \frac{\omega -{{\tilde{\omega }}}}{\tau }\,d\tau - 2y_*^2\int _{z}^{1-r}\nonumber \\&\left( {\mathcal {F}}(y_*,\rho ,\omega )-{\mathcal {F}}({{\tilde{y}}}_*, {{\tilde{\rho }}},{{\tilde{\omega }}})\right) \,d\tau , \end{aligned}$$

4.159 $$\begin{aligned} \rho (z)-{{\tilde{\rho }}}(z)&= \rho (1-r) - {{\tilde{\rho }}}(1-r)+2y_*^2\int _{z}^{1-r} \left( {\mathcal {G}}(y_*,\rho ,\omega )-{\mathcal {G}}({{{\tilde{y}}}}_*, {{\tilde{\rho }}},{{\tilde{\omega }}})\right) \,d \tau , \end{aligned}$$

where the nonlinearities ${\mathcal {F}}$ and ${\mathcal {G}}$ are defined in (4.137) and (4.138).

We let

$$\begin{aligned} g(z): = \left| \omega (z)-{{\tilde{\omega }}}(z)\right| + \left| \rho (z)-{{\tilde{\rho }}}(z)\right| . \end{aligned}$$

Since $1 - {{\tilde{y}}}_*^2 z^2 {{\tilde{\omega }}}^2 = 1 - y_*^2 z^2 \omega ^2 + z^2\left( \omega ^2-{{\tilde{\omega }}}^2\right) {{\tilde{y}}}_*^2 +z^2\omega ^2\left( y_*^2-{{\tilde{y}}}_*^2\right)$, from (4.155) we conclude

4.160 $$\begin{aligned} 1 - {{\tilde{y}}}_*^2 z^2 {{\tilde{\omega }}}^2&\ge 1 - y_*^2 z^2 \omega ^2 - C \left( g(z)+\left| y_*- {{\tilde{y}}}_*\right| \right) \nonumber \\&\ge \eta - C \left( g(z)+\left| y_*- {{\tilde{y}}}_*\right| \right) . \end{aligned}$$

We let

4.161 $$\begin{aligned} {{\bar{\eta }}}(z) : = \eta - C \left( g(z)+\left| y_*- {{\tilde{y}}}_*\right| \right) . \end{aligned}$$

Clearly, for $\delta >0$ and $|1-r-z|$ sufficiently small, we have ${{\bar{\eta }}}>\frac{\eta }{2}$ by continuity. Let

4.162 $$\begin{aligned} {\bar{Z}} : = \inf _{Z<z<1-r} \left\{ {{\bar{\eta }}}(z)>\frac{\eta }{2}\right\} . \end{aligned}$$

For any $z\ge {\bar{Z}}$ a simple algebraic manipulation and the bounds (4.154), (4.160), and (4.155) give

4.163 $$\begin{aligned} \left| {\mathcal {F}}(y_*,\rho ,\omega ) - {\mathcal {F}}({{{\tilde{y}}}}_*, {{\tilde{\rho }}},{{\tilde{\omega }}}) \right|&\le \frac{C}{\eta {{\bar{\eta }}}}\left( |\omega -{{\tilde{\omega }}}| +|\rho -{{\tilde{\rho }}}| + |y_*-{{{\tilde{y}}}}_*|\right) , \ \ |y_*-{{\tilde{y}}}_*|<\delta , \end{aligned}$$

4.164 $$\begin{aligned} \left| {\mathcal {G}}(y_*,\rho ,\omega ) - {\mathcal {G}}({{{\tilde{y}}}}_*, {{\tilde{\rho }}},{{\tilde{\omega }}}) \right|&\le \frac{C}{\eta {{\bar{\eta }}}}\left( |\omega -{{\tilde{\omega }}}| +|\rho -{{\tilde{\rho }}}| + |y_*-{{{\tilde{y}}}}_*|\right) , \ \ |y_*-{{\tilde{y}}}_*|<\delta . \end{aligned}$$

The identities (4.158)–(4.159), (4.162), and estimates (4.163)–(4.164) now give

4.165 $$\begin{aligned} g(z)&\le g(1-r) + \frac{C}{\eta ^2} |y_*-{{\tilde{y}}}_*| + \frac{3}{\mathring{z}} \int _z^{1-r} |\omega (\tau )-{{\tilde{\omega }}}(\tau )|\,d\tau + \frac{C}{\eta ^2} \int _z^{1-r} g(\tau )\,d\tau \nonumber \\&\le g(1-r) + \frac{C}{\eta ^2} |y_*-{{\tilde{y}}}_*|+ \frac{C}{\eta ^2} \int _z^{1-r} g(\tau )\,d\tau , \ \ z\in [{\bar{Z}}, 1-r] . \end{aligned}$$

It follows by a Grönwall argument and (4.162) that

$$\begin{aligned} g(z)&\le \left( g(1-r) + \frac{C}{\eta ^2} |y_*-{{\tilde{y}}}_*|\right) \frac{C}{\eta ^2} e^{ \frac{C}{\eta ^2}(1-r-z)}, \ \ z\in [{\bar{Z}}, 1-r]. \end{aligned}$$

We note that for any given $\delta '>0$, there exists a $\delta >0$ such that $g(1-r)<\delta '$ for all $|y_*-{{\tilde{y}}}_*|<\delta$. Therefore, for any given $\epsilon >0$ we can choose a $\delta =\delta (\eta ,\epsilon )$ sufficiently small so that for all $|y_*-{{\tilde{y}}}_*|<\delta$ we have the bound

$$\begin{aligned} g(z)<\epsilon , \ \ {\bar{Z}} < z \le 1-r. \end{aligned}$$

However, by (4.161) we then have

$$\begin{aligned} {{\bar{\eta }}}({\bar{Z}}) \ge \eta - C\left( \epsilon +\delta \right) >\frac{\eta }{2}, \ \ \text { for }\delta \text { sufficiently small}. \end{aligned}$$

This is only possible if ${\bar{Z}} = Z$. This gives a uniform lower bound for $1 - {{\tilde{y}}}_*^2 z^2 {{\tilde{\omega }}}^2$ on $(Z,1-r]$ and this contradicts the assumption $Z=s({{\tilde{y}}}_*)$. Therefore $Z=\mathring{z}$ and $s({{\tilde{y}}}_*)$ is strictly smaller that $\mathring{z}$ by Lemma 4.3. Since $\mathring{z}>s(y_*)$ is arbitrary it follows that for any $\varepsilon >0$ there exists a $\delta >0$ such that $|{{\tilde{y}}}_*-y_*|<\delta$ implies $s({{\tilde{y}}}_*)-s(y_*)<\varepsilon$, which is equivalent to upper semi-continuity. If $s(y_*)=0$ this implies the continuity of $s(\cdot )$ at $y_*$.

*Proof of part (b).* Since *z* is a fixed distance away from the sonic time $s(y_*)$, there exists a constant ${{\tilde{\eta }}}>0$ such that $1-\tau ^2y_*^2\omega (\tau ;y_*)^2>{{\tilde{\eta }}}$ for all $\tau \in [z,1-r]$. By the proof of part (a) there exists a neighbourhood of $y_*$ depending on ${{\tilde{\eta }}}$ and *z*, such that all LP- type solutions launched from that neighbourhood have a sonic time strictly less than $s(y_*)$. The claim now follows from (4.165).

*Proof of part (c).* This is again a consequence of the arguments in the proof of part (a). By Lemma 4.3 it is clear that there exists a $T=T(\eta ,Z)$ such that $s(y_*^n)<Z-T$ for all $n\in {\mathbb {N}}$. On the other hand, due to the lower bound (4.151) and the proof of part (a) there exists a $\delta =\delta (\eta ,Z)$ such that for all $|{{\tilde{y}}}_*-y_*^n|<\delta$ the sonic time $s({{\tilde{y}}}_*)<Z-T$ for some, possibly smaller time $T=T(\eta ,Z)>0$. Letting *n* large enough, this concludes the proof. $\quad \square$

### The set *Y* and the minimality property

We now partition the interval [2, 3] in the the sets ${\mathcal {X}},{\mathcal {Y}},{\mathcal {Z}}$ that will play an important role in our analysis. We let

4.166 $$\begin{aligned}&{\mathcal {X}}: = \left\{ y_*\in [2,3]\,\Big | \inf _{z\in (s(y_*),1)}\omega (z;y_*)>\frac{1}{3} \right\} , \end{aligned}$$

4.167 $$\begin{aligned}&{\mathcal {Y}}: = \left\{ y_*\in [2,3]\,\big | \ \exists z\in (s(y_*), 1) \ \text {such that } \omega (z;y_*)=\frac{1}{3}\right\} \end{aligned}$$

4.168 $$\begin{aligned}&{\mathcal {Z}}: = \left\{ y_*\in [2,3]\,\Big | \omega (z;y_*)>\frac{1}{3} \ \text { for all } \ z\in (s(y_*),1) \ \text { and } \ \inf _{z\in (s(y_*),1)}\omega (z;y_*)\le \frac{1}{3}\right\} . \end{aligned}$$

Clearly $[2,3] = {\mathcal {X}}\cup {\mathcal {Y}}\cup {\mathcal {Z}}$ and the sets ${\mathcal {X}},{\mathcal {Y}},{\mathcal {Z}}$ are disjoint. We introduce the fundamental set $Y\subset {\mathcal {Y}}$

4.169 $$\begin{aligned} Y : = \left\{ y_*\in [2,3]\,\big | \ \exists \, z\in (s({{\tilde{y}}}_*), 1) \ \text {such that } \omega (z;{{\tilde{y}}}_*)=\frac{1}{3} \ \text { for all } \ {{\tilde{y}}}_*\in [y_*,3]\right\} , \end{aligned}$$

and let

4.170 $$\begin{aligned} {\bar{y}}_*&: = \inf _{y_*\in Y} y_*. \end{aligned}$$

The next statement shows that sets ${\mathcal {Y}}$ and ${\mathcal {X}}$ are not empty.

#### Lemma 4.6

*(a)*: There exists an $\epsilon >0$ such that $(3-\epsilon ,3]\subset Y\subset {\mathcal {Y}}$
*(b)*: $$2\in {\mathcal {X}}$$.

#### Proof

*Proof of part (a) * By the mean value theorem, we write $\omega (z;y_*)$ as

$$\begin{aligned} \omega (z;y_*) = \frac{1}{y_*} + \omega '({\bar{z}}; y_*) (z-1), \ z\in (s(y_*), 1) \end{aligned}$$

for some ${\bar{z}}\in (z,1)$. From (1.28), we have $\omega '(1; y_*) =1-\frac{2}{y_*}$ with $\omega '(1; 3) =\frac{1}{3}$. By Theorem 2.10 and Lemma 2.11, there exist small enough $r>0$ and $\epsilon _1>0$ such that $\omega '(z; y_*)>\frac{1}{6}$ for all $z\in (1-r,1]$ and $y_*\in (3-\epsilon _1,3]$. Then for $z\in (1-r,1]$ and $y_*\in (3-\epsilon _1,3]$, we have

$$\begin{aligned} \omega (z;y_*) \le \frac{1}{y_*} +\frac{1}{6} (z-1). \end{aligned}$$

Note $\frac{1}{y_*} + \frac{1}{6} (z-1) = \frac{1}{3}$ when $z=z^*(y_*)= 1- \frac{2(3-y_*)}{y_*}$. Therefore for all $y_*\in (3-\epsilon , 3]$ with $\epsilon = \min \{ \epsilon _1, \frac{3r}{2+r}\}$, there exists ${{\tilde{z}}}\ge z^*(y_*)$ such that $\omega ({{\tilde{z}}};y_*)=\frac{1}{3}$, which shows $(3-\epsilon ,3]\subset Y\subset {\mathcal {Y}}$.

*Proof of part (b).* Let $y_*=2$ and denote $\omega (\cdot ;2)$ by $\omega$. First we rewrite (1.24) as

$$\begin{aligned} z\omega '&= 1-2\omega -\omega + \frac{2y_*^2z^2\omega ^2}{1-y_*^2z^2\omega ^2}(\rho -\omega ) \\&= 1- 2\omega - \omega \left[ \frac{1- (y_*z\rho )^2 + (y_*z \rho -y_*z \omega )^2 }{1-y_*^2z^2\omega ^2}\right] . \end{aligned}$$

By Lemma 4.2, we have $z\omega ' \le 1-2\omega$, which implies that $\omega >\frac{1}{2}$ is an invariant set. On the other hand from (1.28) we know that

$$\begin{aligned} \omega (1)=\frac{1}{2}, \ \ \ \omega '(1)=0, \ \ \ \omega ''(1) = \frac{1}{2} \end{aligned}$$

and hence $\omega >\frac{1}{2}$ on $(1-\eta , 1)$ for sufficiently small $\eta >0$. Therefore, we conclude that $\inf _{z\in (s(2),1)}\omega (z;2)=\frac{1}{2}$ and $2\in {\mathcal {X}}$. $\square$

#### Definition 4.7

For any $y_*>0$ we define

4.171 $$\begin{aligned} z_{\frac{1}{3}}&= z_{\frac{1}{3}}(y_*) : = \inf \left\{ z \in (s(y_*), 1) \, \big | \ \ \omega (\tau ;y_*)>\frac{1}{3} \ \text { for } \ \tau \in (z,1) \right\} . \end{aligned}$$

#### Remark 4.8

Geometrically, if we follow the solution curve $z\mapsto \omega (z;y_*)$ starting at $z=1$ and going to the left, point $z_{\frac{1}{3}}$ is the first time this curve crosses the value $\frac{1}{3}$ from above. By the definition of ${\mathcal {Y}}$, for any $y_*\in {\mathcal {Y}}$ there exists an $z_{\frac{1}{3}}\in (s(y_*), 1]$ such that

$$\begin{aligned} \omega (z_{\frac{1}{3}};y_*) = \frac{1}{3}. \end{aligned}$$

Therefore, for any $y_*\in [2,3]$ we have the following possibilities:

1. $$y_*\in {\mathcal {Y}}$$ and therefore $$z_{\frac{1}{3}}(y_*)>s(y_*)\ge 0$$.
2. $y_*\in [2,3]{\setminus } {\mathcal {Y}}$ and $z_{\frac{1}{3}}(y_*)=s(y_*)>0$. In this case we must have
  4.172 $$\begin{aligned} \omega (z_{\frac{1}{3}}(y_*);y_*)=\omega (s(y_*);y_*)>\frac{1}{3}; \end{aligned}$$
  otherwise if $\omega (z_{\frac{1}{3}}(y_*);y_*)=\frac{1}{3}$ then $\omega (z_{\frac{1}{3}}(y_*);y_*) z_{\frac{1}{3}}(y_*)=\frac{1}{3} z_{\frac{1}{3}}(y_*)<1$ and thus $s(y_*)<z_{\frac{1}{3}}(y_*)$.
3. $$y_*\in [2,3]{\setminus } {\mathcal {Y}}$$ and $$z_{\frac{1}{3}}(y_*)=s(y_*)=0$$.

The sets ${\mathcal {Y}}$ and ${\mathcal {Z}}$ enjoy several important properties which we prove in the next lemma.

#### Lemma 4.9

*(a)*: For any $y_*\in {\mathcal {Y}}\cup {\mathcal {Z}}$ we have
  4.173 $$\begin{aligned} \omega (z;y_*)&<\rho (z;y_*), \ \ z\in (s(y_*), 1), \end{aligned}$$
  4.174 $$\begin{aligned} \omega (z;y_*)&<\frac{1}{3}, \ \ z\in (s(y_*), z_{\frac{1}{3}}(y_*)), \end{aligned}$$
  where (4.174) is considered trivially true in the case $s(y_*)=z_{\frac{1}{3}}(y_*)$.
*(b)*: For any $y_*\in {\mathcal {Y}}$ we have $\omega '(z_{\frac{1}{3}}(y_*);y_*)>0$. Moreover, the set ${\mathcal {Y}}$ is relatively open in [2, 3].

#### Proof

*Proof of* (4.173) Let $y_*\in {\mathcal {Y}}\cup {\mathcal {Z}}$. By (1.28) we know that $\omega (z)<\rho (z)$ for all $z\in [1-{\bar{r}},1)$ for some ${\bar{r}}\le r$, where *r* is given by Theorem 2.10. By way of contradiction, assume that there exists $z_c\in (s(y_*), 1)$ such that

4.175 $$\begin{aligned} \omega (z_c) = \rho (z_c), \ \ \rho (z)>\omega (z), \ z\in (z_c,1). \end{aligned}$$

We distinguish three cases.

*Case 1:* $z_c\in (z_{\frac{1}{3}},1)$. In this case we conclude from (1.24) that $\omega '(z_c)<0$ by (4.175) and (4.171) and from (1.23) and (4.175) $\rho '(z_c)=0$. In particular $(\rho -\omega )'(z_c)>0$ and locally strictly to the left of $z_c$ we have

4.176 $$\begin{aligned} \omega '<0, \ \rho -\omega <0, \ \rho '>0, \ \omega >\frac{1}{3}. \end{aligned}$$

We note that $\rho '>0$ follows from $\rho -\omega <0$ and (1.23), while $\omega >\frac{1}{3}$ is implied by the assumption $z_c\in (z_{\frac{1}{3}},1)$. It is easy to see that the conditions (4.176) are dynamically trapped, and since $\omega '<0$ we conclude that $\omega$ stays strictly bounded away from $\frac{1}{3}$ from above for all $z\in (z_{\frac{1}{3}},1)$. This is a contradiction to the assumption $y_*\in {\mathcal {Y}}\cup {\mathcal {Z}}$.

*Case 2:* $z_c=z_{\frac{1}{3}}$. In this case $y_*\in {\mathcal {Y}}$ necessarily and

4.177 $$\begin{aligned} \omega (z_{\frac{1}{3}}) = \rho (z_{\frac{1}{3}}) = \frac{1}{3}. \end{aligned}$$

However, since $\rho -\omega >0$ for $z\in (z_{\frac{1}{3}},1)$ equation (1.23) implies $\rho '<0$ on $(z_{\frac{1}{3}},1)$ and therefore $\rho (z_{\frac{1}{3}})>\rho (1)=\frac{1}{y_*} \ge \frac{1}{3}$, since $y_*[2,3]$. This is a contradiction to (4.177).

*Case 3:* $z_c \in (s(y_*),z_{\frac{1}{3}})$. In this case $y_*\in {\mathcal {Y}}$ necessarily. Since $z_c<z_{\frac{1}{3}}$ we know that $\rho -\omega >0$ locally around $z_{\frac{1}{3}}$. Therefore, by (1.24)–(1.23) and (4.171) we have

4.178 $$\begin{aligned} \omega '>0, \ \rho -\omega >0, \ \omega <\frac{1}{3} \ \ \text { on } \ (z_{\frac{1}{3}}-\varepsilon , z_{\frac{1}{3}}) \end{aligned}$$

for a sufficiently small $\varepsilon >0$. The region described by (4.178) is dynamically trapped and we conclude that $\rho -\omega >0$ on $(s(y_*),z_{\frac{1}{3}})$. This is a contradiction, thus completing the proof of (4.173). Inequality (4.174) follows by a similar argument, since the property (4.178) is dynamically preserved and all three properties are easily checked to hold locally to the left of $z_{\frac{1}{3}}(y_*)$.

*Proof of part (b).* For any $y_*\in {\mathcal {Y}}$ by part (a) and (1.24) we have $\omega '(z_{\frac{1}{3}}(y_*);y_*)>0$. Therefore there exists a $\delta >0$ sufficiently small so that $\omega (z;y_*)<\frac{1}{3}$ for all $z\in (z_{\frac{1}{3}}(y_*)-\delta ,z_{\frac{1}{3}}(y_*))$. By the proof of Proposition 4.5 there exists a small neighbourhood of $y_*$ such that $\omega (z;y_*)<\frac{1}{3}$ for some $z\in (z_{\frac{1}{3}}(y_*)-\delta ,z_{\frac{1}{3}}(y_*))$. Therefore ${\mathcal {Y}}$ is open. $\quad \square$

Another remarkable feature of the sets ${\mathcal {Y}}$ and ${\mathcal {Z}}$ is the following uniform lower bound on the distance to the sonic line at points *z* larger than $z_{\frac{1}{3}}(y_*)$.

#### Lemma 4.10

There exists a constant $\eta >0$ such that

$$\begin{aligned} 1 - y_*^2 z^2 \omega (z;y_*)^2 > \eta , \ \ y_*\in {\mathcal {Y}}\cup {\mathcal {Z}}, \ \ z\in (z_{\frac{1}{3}}(y_*),1-r], \end{aligned}$$

where *r* is the constant given in Theorem 2.10.

#### Proof

It is clear that there exists an $\eta >0$ such that $1 - y_*^2 z^2 \omega (z;y_*)^2 > \eta$ at $z=1-r$ for all $y_*\in [2,3]$. By (4.173) and (4.129) and since $\omega (z;{\bar{y}}_*)>\frac{1}{3}$ for all $z\in (z_{\frac{1}{3}}(y_*),1-r]$, we have

$$\begin{aligned}&1 - y_*^2 z^2\omega (z;y_*)^2> 1 - y_*^2 z^2\rho (z;y_*)^2> 1- y_*^2 (1-r)^2\rho (1(1-r);y_*)^2>\eta ,\\&\quad \ \ z\in (z_{\frac{1}{3}}(y_*),1-r], \end{aligned}$$

for all $$y_*\in {\mathcal {Y}}\cup {\mathcal {Z}}$$. $$\quad \square $$

#### Remark 4.11

It is easily seen from the proof that the uniform-in-$y_*$ lower bound from Lemma 4.10 holds as long as $\omega (z;y_*)\ge 0$.

We now use the previously shown regularity properties to obtain the key result, which states that $s({\bar{y}}_*)=0$, i.e. the LP-type solution associated with ${\bar{y}}_*=\inf Y$ extends to the left from $z=1$ all the way to $z=0$.

#### Proposition 4.12

(Existence up to the origin). Recall ${\bar{y}}_*$ defined in (4.170). The solution $(\omega (z;{\bar{y}}_*), \rho (z;{\bar{y}}_*))$ exists on (0, 1], i.e.

$$\begin{aligned} s({\bar{y}}_*)=0. \end{aligned}$$

#### Proof

*Case 1:* $z_{\frac{1}{3}}({\bar{y}}_*)=0$. In this case we are done as by definition $0\le s({\bar{y}}_*)\le z_{\frac{1}{3}}({\bar{y}}_*)=0$.

*Case 2:* $z_{\frac{1}{3}}({\bar{y}}_*)>s({\bar{y}}_*)>0$. In this case ${\bar{y}}_*\in Y$ and

$$\begin{aligned} \omega (z_{\frac{1}{3}}({\bar{y}}_*);{\bar{y}}_*) = \frac{1}{3} \end{aligned}$$

There exists a $\delta >0$ such that

4.179 $$\begin{aligned} \omega (z;{\bar{y}}_*)<\frac{1}{3}, \ \ z\in (z_{\frac{1}{3}}({\bar{y}}_*)-\delta ,z_{\frac{1}{3}}({\bar{y}}_*)), \end{aligned}$$

by Lemma 4.9. Let now $\{y^n_*\}_{n\in {\mathbb {N}}}\subset [2,3]{\setminus } Y$ satisfy

$$\begin{aligned} \lim _{n\rightarrow \infty } y_*^n = {\bar{y}}_*. \end{aligned}$$

Note that we choose $\{y^n_*\}_{n\in {\mathbb {N}}}\subset {\mathcal {X}}\cup {\mathcal {Z}}$, which is possible by the openess of *Y*, ${\mathcal {Y}}$, and the definition of ${\bar{y}}_*$, see (4.170). Since by Proposition 4.5$s({\bar{y}}_*)\ge \limsup _{n\rightarrow \infty }s(y_*^n)$ and $z_{\frac{1}{3}}({\bar{y}}_*)>s({\bar{y}}_*)$ it follows that there exists $N\in {\mathbb {N}}$ sufficiently large such that (after passing to a subsequence) $s(y_*^n)<z_{\frac{1}{3}}({\bar{y}}_*)-\delta$ for all $n>N$, where we have chosen a possibly smaller $\delta$. By part (b) of Proposition 4.5 it follows that for any $z\in (z_{\frac{1}{3}}({\bar{y}}_*)-\delta ,z_{\frac{1}{3}}({\bar{y}}_*))$ $\omega (z;{\bar{y}}_*)=\lim _{n\rightarrow \infty }\omega (z;y_*^n) \ge \frac{1}{3}$, a contradiction to (4.179).

*Case 3:* $z_{\frac{1}{3}}({\bar{y}}_*)=s({\bar{y}}_*)>0$. In this case ${\bar{y}}_*\in [2,3]{\setminus } Y$ and therefore, by definition of ${\bar{y}}_*$ we have ${\bar{y}}_*\in {\mathcal {X}}\cup {\mathcal {Z}}$. By (4.172) we have $\omega (s({\bar{y}}_*);{\bar{y}}_*)>\frac{1}{3}$. Let now $\{y^n_*\}_{n\in {\mathbb {N}}}\subset Y$ satisfy

$$\begin{aligned} \lim _{n\rightarrow \infty } y_*^n = {\bar{y}}_*. \end{aligned}$$

Define

$$\begin{aligned} {\bar{z}}_{\frac{1}{3}} : = \limsup _{n\rightarrow \infty } z_{\frac{1}{3}}(y_*^n) \end{aligned}$$

We need to distinguish two subcases.

*Subcase 1:* ${\bar{z}}_{\frac{1}{3}}>0$. By Lemma 4.9 and Remark 4.8 we have $s(y_*^n)<z_{\frac{1}{3}}(y_*^n)$. By Lemma 4.10 there exists a positive number $\eta$ such that

$$\begin{aligned} 1 - z^2(y_*^n)^2\omega (z;y_*^n)^2 > \eta , \ \ n\in {\mathbb {N}}, \ \ z\in [z_{\frac{1}{3}}(y_*^n), 1-r]. \end{aligned}$$

Upon passing to a subsequence $\{y_*^n\}_{n\in {\mathbb {N}}}$ such that $\lim _{n\rightarrow \infty }z_{\frac{1}{3}}(y_*^n)={\bar{z}}_{\frac{1}{3}}$, by part (c) of Proposition 4.5 we conclude that there exists a $T=T(\eta ,{\bar{z}}_{\frac{1}{3}})>0$ such that $s({\bar{y}}_*), s({\bar{y}}_*^n)<{\bar{z}}_{\frac{1}{3}}-T$, $n\in {\mathbb {N}}$. In particular, for any $z\in ({\bar{z}}_{\frac{1}{3}}-T,{\bar{z}}_{\frac{1}{3}})$ we conclude by part (b) of Proposition 4.5 that $\omega (z;{\bar{y}}_*)=\lim _{n\rightarrow \infty }\omega (z;y_*^n)\le \frac{1}{3}$, a contradiction to ${\bar{y}}_*\notin Y$.

*Subcase 2:* ${\bar{z}}_{\frac{1}{3}}=0$. For any fixed $Z>0$ we can apply the argument from Subcase 1 to conclude that the $s({\bar{y}}_*)<Z$. Therefore $s({\bar{y}}_*)=0$ in this case. $\quad \square$

#### Lemma 4.13

(Continuity of ${\mathcal {Y}}\ni y_*\mapsto z_{\frac{1}{3}}(y_*)$). The map

$$\begin{aligned} {\mathcal {Y}}\ni y_*\mapsto z_{\frac{1}{3}}(y_*) \end{aligned}$$

is continuous and

4.180 $$\begin{aligned} \lim _{Y\ni y\rightarrow {\bar{y}}_*} z_{\frac{1}{3}}(y) = 0 = z_{\frac{1}{3}}({\bar{y}}_*). \end{aligned}$$

#### Proof

Let $y_*\in {\mathcal {Y}}$. By Lemma 4.10 there exists a $\delta >0$ such that $s({{\tilde{y}}}_*)<z_{\frac{1}{3}}(y_*)-\delta$ for all ${{\tilde{y}}}_*$ in an open neighbourhood of $y_*$. Since by part (b) of Lemma 4.9$\omega '(z_{\frac{1}{3}}(y_*);y_*)>0$, we may now use the Implicit Function Theorem to conclude that the map $y_*\mapsto z_{\frac{1}{3}}(y_*)$ is in fact $C^1$.

To show (4.180) assume the opposite: there exists a sequence $\{y_*^n\}_{n\in {\mathbb {N}}}\subset Y\subset {\mathcal {Y}}$ such that $\lim _{n\rightarrow \infty } y_*^n={\bar{y}}_*$, but

$$\begin{aligned} \alpha :=\liminf _{n\rightarrow \infty } z_{\frac{1}{3}}(y_*^n)>0. \end{aligned}$$

Upon passing to a subsequence, we may assume without loss of generality that $\lim _{n\rightarrow \infty } z_{\frac{1}{3}}(y_*^n)=\alpha$ and $z_{\frac{1}{3}}(y_*^n)>\frac{\alpha }{2}$ for all $n\in {\mathbb {N}}$. By Lemma 4.10 and Proposition 4.5 there exists an $\epsilon =\epsilon (\alpha ,\eta )$ (here $\eta$ is the constant from Lemma 4.10) such that $s(y^n_*)<z_{\frac{1}{3}}(y_*^n)-3\epsilon$ for all $n\in {\mathbb {N}}$. We infer that (upon possibly passing to a subsequence) $\omega (z;y_*^n)<\frac{1}{3}$ for all $z\in [\alpha -2\epsilon ,\alpha -\epsilon ]$. Thus by continuity of the map $[2,3]\ni y_*\mapsto \omega (z;y_*)$ we conclude that $\omega (z;{\bar{y}}_*)\le \frac{1}{3}$ for $z\in [\alpha -2\epsilon ,\alpha -\epsilon ]$, which implies ${\bar{y}}_*\in Y$. By part (b) of Lemma 4.9 there is an open neighbourhood of ${\bar{y}}_*$ that belongs to *Y*, which is a contradiction to the minimality property of ${\bar{y}}_*$ and part (b) of Lemma 4.6. $\quad \square$

### Properties of the solution from the origin to the right

In order to complete the intersection argument in Sect. 4.4 we must better understand the solutions emanating from $z=0$ to the right. Recall that $(\rho _-(\cdot ;\rho _0),\omega _-(\cdot ;\rho _0))$ is the unique local solution to (1.23)–(1.24) satisfying the boundary conditions

4.181 $$\begin{aligned} \rho _-(0) = \rho _0>0, \ \ \omega _-(0)= \frac{1}{3}; \end{aligned}$$

existence and uniqueness are given by Theorem 2.12. Let $s_-(\rho _0)$ denote the sonic time (from the left), i.e.

$$\begin{aligned} s_-(\rho _0) : = \sup _{z\ge 0} \left\{ z \, \big | \ y_*z \omega _-(z;\rho _0)<1\right\} . \end{aligned}$$

We then have the following a priori bounds on $(\rho _-,\omega _-)$.

#### Lemma 4.14

Let $\rho _-(0)=\rho _0>\frac{1}{3}$, $\omega _-(0)=\frac{1}{3}$, and $y_*\in [2,3]$. The solution $(\rho _-(z;\rho _0),\omega _-(z;\rho _0))$ to (1.23)–(1.24) with the initial data (4.181) exists on the interval $[0,s_-(\rho _0))$ and satisfies the following bounds:

4.182 $$\begin{aligned} \omega _-(z;\rho _0)&>\frac{1}{3}, \ \ z\in [0,s_-(\rho _0)) \end{aligned}$$

4.183 $$\begin{aligned} \rho _-(z;\rho _0) + \omega _-(z;\rho _0)&< \rho _0 + \frac{1}{3}, \ \ z\in [0,s_-(\rho _0)) \end{aligned}$$

4.184 $$\begin{aligned} \rho _-(z;\rho _0) \omega _-(z;\rho _0)&< \frac{1}{3} \rho _0, \ \ z\in [0,T_*) \end{aligned}$$

4.185 $$\begin{aligned} \rho _-(z;\rho _0)&>\omega _-(z;\rho _0), \ \ z\in [0,s_-(\rho _0)) \end{aligned}$$

4.186 $$\begin{aligned} \rho _-'(z;\rho _0)&<0, \ \ z\in [0,s_-(\rho _0)). \end{aligned}$$

#### Proof

We suppress the $\rho _0$-dependence in the notation for $(\rho _-,\omega _-)$.

*Proof of* (4.182). Since $\omega ''(0)>0$ for $\rho _0>\frac{1}{3}$ for $0< z\ll 1$ (by (2.104)) it is clear that (4.182) is true for any sufficiently small $z>0$. Suppose now that (4.182) is wrong and let $0<z_1<s_-(\rho _0)$ be the first $z_1$ such that $\omega _-(z_1)=\frac{1}{3}$ and $\frac{1}{3}<\omega _-(z)$ for $0<z<z_1$. Then $\omega _-'(z_1)\le 0$. First suppose $\omega _-'(z_1)<0$. Then from (1.23)–(1.24) we deduce that $\rho _-(z_1)<\omega _-(z_1)=\frac{1}{3}$ and $\rho '_-(z_1)>0$. Hence, there should exist $0<z_2<z_1$ such that $\rho _-'(z_2)=0$ and $\rho _-(z_2)< \rho _-(z_1)<\frac{1}{3}$. Then from (1.23), $\omega _-(z_2)=\rho _-(z_2)$, which is a contradiction to the definition of $z_1$. Next let $\omega _-'(z_1)=0$. Then $\rho _-(z_1)=\omega _-(z_1)=\frac{1}{3}$ and also $\rho '(z_1)=0$. Since $z_1$ is away from the sonic line, $(\rho _-,\omega _-,)$ is smooth and the conditions $\rho _-(z_1)=\omega _-(y_1)=\frac{1}{3}$ and $\omega _-'(z_1)=\rho _-'(z_1)=0$ give $\rho _-=\omega _-=\frac{1}{3}$ in an open neighborhood, which is a contradiction. Here we have used uniqueness and the existence of the Friedman solution $(\rho _F,\omega _F)\equiv (\frac{1}{3},\frac{1}{3})$, see Remark 1.3.

*Proof of* (4.183). This follows from

$$\begin{aligned} (\rho _-+\omega _-)' = \frac{1-3\omega _-}{z} - \frac{2y_*^2z\omega _-}{ 1- (y_*z\omega _-)^2} (\rho _--\omega _-)^2 \end{aligned}$$

which is negative for $0<z<s_-(\rho _0)$ since $\omega _->\frac{1}{3}$.

*Proof of* (4.184). This follows from

4.187 $$\begin{aligned} (\rho _-\omega _-)' = \frac{\rho _-(1-3\omega _-)}{z} \end{aligned}$$

which is negative for $0<z<s_-(\rho _0)$ since $\omega _->\frac{1}{3}$.

*Proof of* (4.185) *and* (4.186). This follows from

$$\begin{aligned} (\rho _--\omega _-)' = \frac{3\omega _--1}{z} - \frac{2y_*^2z\omega _-(\rho _-+\omega _-)}{ 1- (y_*z\omega _-)^2} (\rho _--\omega _-) \end{aligned}$$

by integrating in *z*. Claim (4.186) follows from (1.23). $\quad \square$

#### Lemma 4.15

Let $\rho _0>\frac{1}{3}$ be given and consider the unique solution $(\rho _-(z;\rho _0),\omega _-(z;\rho _0))$ to the initial-value problem (1.23)–(1.24), (4.181). Assume that $\rho _-(z_0;\rho _0)>\frac{1}{y_*z_0}$ for some $z_0\in (0,s_-(\rho _0))$. Then

$$\begin{aligned} \rho _-(z;\rho _0) >\frac{1}{y_*z}, \ \ z\in [z_0,s_-(\rho _0)). \end{aligned}$$

#### Proof

Just like in the proof of Lemma 4.2 we consider

$$\begin{aligned} f_-(z) : = 1 - y_*z \rho _-(z). \end{aligned}$$

Equation (4.131) then reads

4.188 $$\begin{aligned} f_-'(z) + f_-(z) \frac{2zy_*^2\omega _-\rho _-}{1-y_*^2z^2\omega _-^2} = - \frac{y_*(1-y_*z \omega _-)^2 \rho _-}{1-y_*^2z^2\omega _-^2} \end{aligned}$$

By our assumptions $f_-(z_0)<0$. Since the right-hand side of (4.188) is negative, we conclude

$$\begin{aligned} \frac{d}{dz}\left( f_-(z) \exp \left( \int _{z_0}^z \frac{2\tau y_*^2\omega _-\rho _-}{1-y_*^2\tau ^2\omega _-^2}\,d\tau \right) \right) <0, \ \ z\in [z_0,s_-(\rho _0)), \end{aligned}$$

which gives the claim. $\quad \square$

In the following lemma we identify a spatial scale $z_0\sim \frac{1}{\rho _0}$ over which we obtain quantitative lower bounds on the density $\rho _-$ over $[0,z_0]$.

#### Lemma 4.16

Let $\rho _0>\frac{1}{3}$ and $y_*\in [2,3]$ be given and consider the unique solution $(\rho _-(z;\rho _0),\omega _-(z;\rho _0))$ to the initial-value problem (1.23)–(1.24), (4.181). For any $\rho _0>\frac{1}{3}$ let

4.189 $$\begin{aligned} z_0=z_0(\rho _0): = {\left\{ \begin{array}{ll} \frac{\sqrt{3} }{\sqrt{2} y_*\rho _0} &{} \rho _0> 1; \\ \frac{\sqrt{3} }{\sqrt{2} y_*}, &{} \frac{1}{3} < \rho _0\le 1. \end{array}\right. } \end{aligned}$$

Then $s_-(\rho _0)>z_0$ for all $\rho _0>\frac{1}{3}$ and

4.190 $$\begin{aligned} \rho _-(z;\rho _0) \ge {\left\{ \begin{array}{ll} \rho _0 \exp \left( - \rho _0^{-1}\right) , &{} \rho _0>1;\\ \rho _0 \exp \left( -1\right) , &{} \frac{1}{3} < \rho _0\le 1, \end{array}\right. } \ \ z\in [0, z_0]. \end{aligned}$$

Moreover, there exists an $R>1$ such that for all $\rho _0>R$ we have

4.191 $$\begin{aligned} \rho _-(z_0;\rho _0)&>\frac{1}{y_*z_0}. \end{aligned}$$

#### Proof

Equation (1.23) is equivalent to

4.192 $$\begin{aligned} \rho _-(z) = \rho _0 \exp \left( -\int _0^z\frac{2y_*^2 \tau \omega _-(\rho _--\omega _-)}{1- y_*^2 \tau ^2 \omega _-^2}\,d\tau \right) . \end{aligned}$$

By Lemma 4.14 we have the following bounds on the interval $(0, s_-(\rho _0))$

4.193 $$\begin{aligned} \omega _-<\rho _-&<\rho _0, \end{aligned}$$

4.194 $$\begin{aligned} \frac{1}{3}<\omega _-&<\sqrt{\frac{\rho _0}{3}}. \end{aligned}$$

In particular, $0<\rho _--\omega _-<\rho _0$. Therefore, for any $0\le z\le z_0$ using (4.194) we have

4.195 $$\begin{aligned} 1- y_*^2 z^2 \omega _-^2 \ge 1 - y_*^2 z_0^2 \frac{\rho _0}{3} =1 - \frac{1}{2\rho _0}>\frac{1}{2}, \end{aligned}$$

if $\rho _0>1$. In the case $\rho _0\in (\frac{1}{3},1]$ estimate analogous to (4.195) gives the same lower bound and thus $s_-(\rho _0)>z_0$ for all $\rho _0>0$. From (4.193), (4.184), and (4.195) for any $z\in [0,z_0]$ we obtain

$$\begin{aligned} \int _0^z\frac{2y_*^2 \tau \omega _-(\rho _--\omega _-)}{1- y_*^2 \tau ^2 \omega _-^2}\,d\tau \le \frac{4y_*^2 \rho _0}{3} \int _0^z\tau \,d\tau \le \frac{2y_*^2 \rho _0}{3} z_0^2 = {\left\{ \begin{array}{ll} \rho _0^{-1}, &{} \rho _0>1;\\ \rho _0 \le 1, &{} \frac{1}{3} < \rho _0\le 1. \end{array}\right. } \end{aligned}$$

Plugging the above bound in (4.192) we obtain (4.190). From (4.190) the bound (4.191) follows if $\exp \left( -\rho _0^{-1}\right) >\frac{\sqrt{2}}{\sqrt{3}},$ which is clearly true for sufficiently large $\rho _0$. $\quad \square$

#### Remark 4.17

Since the mapping $\rho _0\mapsto z_0(\rho _0)$ from (4.189) is nonincreasing, it follows that for any fixed $\rho _0>\frac{1}{3}$ we have the uniform bound on the sonic time:

$$\begin{aligned} s_-({{\tilde{\rho }}}_0)>z_0({{\tilde{\rho }}}_0)\ge z_0(\rho _0), \ \ \text { for all }\ \ \frac{1}{3} <{{\tilde{\rho }}}_0\le \rho _0. \end{aligned}$$

The following lemma shows the crucial monotonicity property of $\rho _-(\cdot ;\rho )$ with respect to $\rho _0$ on a time-scale of order $\sim \rho _0^{-\frac{3}{4}}$.

#### Lemma 4.18

Let $y_*\in [2,3]$. There exists a sufficiently small $\eta >0$ such that for all $\rho _0\ge \frac{1}{3}$

$$\begin{aligned} \partial _{\rho _0}\rho _-(z;\rho _0)>0 \ \ \text { for all } \ z\in [0, \eta \rho _0^{-\frac{3}{4}}]. \end{aligned}$$

#### Proof

We introduce the short-hand notation $\partial \rho _- = \partial _{\rho _0}\rho _-$ and $\partial \omega _- = \partial _{\rho _0}\omega _-$. It is easy to check that $(\partial \rho _-,\partial \omega _-)$ solve

4.196 $$\begin{aligned} \partial \omega _-'&= - \frac{3}{z} \partial \omega _- + \frac{4y_*^2 z\omega _-(\rho _- -\omega _-)}{(1-y_*^2 z^2 \omega _-^2)^2} \partial \omega _-- \frac{2y_*^2 z\omega _-^2}{1-y_*^2 z^2 \omega _-^2} \partial \omega _-+\frac{2y_*^2 z \omega _-^2}{1-y_*^2 z^2 \omega _-^2}\partial \rho _- \end{aligned}$$

4.197 $$\begin{aligned} \partial \rho _-'&= - \left( \frac{2y_*^2 z \omega _-(\rho _--\omega _-)}{1-y_*^2 z^2 \omega _-^2} + \frac{2y_*^2 z \omega _- \rho _-}{1-y_*^2 z^2 \omega _-^2} \right) \partial \rho _-\nonumber \\&\ \ \ \ - \left( \frac{2y_*^2 z (\rho _--\omega _-)\rho _-}{1-y_*^2 z^2 \omega _-^2} - \frac{2y_*^2 z \omega _- \rho _-}{1-y_*^2 z^2 \omega _-^2} + \frac{4 y_*^4 z^3 \omega _-^2 \rho _-(\rho _--\omega _-)}{\left( 1-y_*^2 z^2 \omega _-^2\right) ^2}\right) \partial \omega _-. \end{aligned}$$

At $z=0$ we have the initial values

4.198 $$\begin{aligned} \partial \rho _-(0) = 1, \ \ \partial \omega _-(0)=0. \end{aligned}$$

We multiply (4.196) by $\partial \omega _-$ and integrate over the region [0, *z*]. By (4.198) we obtain

4.199 $$\begin{aligned} \frac{1}{2}\partial \omega _-^2(z) + \int _0^z \left( \frac{3}{\tau }+\frac{2\tau y_*^2 \omega _-^2}{1- y_*^2\tau ^2 \omega _-^2 }\right) \partial \omega _-^2 \,d\tau&= \int _0^z \frac{4y_*^2 z\omega _-(\rho _- -\omega _-)}{(1-y_*^2 z^2 \omega _-^2)^2} \partial \omega _-^2\,d\tau \nonumber \\&\ \ \ \ + \int _0^z \left( \frac{2y_*^2 \tau \omega _-^2}{1-y_*^2 \tau ^2 \omega _-^2}\right) \partial \rho _-\partial \omega _-\,d\tau \end{aligned}$$

Since $\omega _-(z)^2\le \frac{\rho _0}{3}$ (by Lemma 4.14) and $y_*\le 3$ we have $1-y_*^2 \tau ^2 \omega _-^2\ge 1 - 3 \tau ^2\rho _0$. Therefore

4.200 $$\begin{aligned} 1-y_*^2 \tau ^2 \omega _-^2 \ge \frac{1}{2}, \text { for any } \ \tau \in [0, (6\rho _0)^{-\frac{1}{2}}]. \end{aligned}$$

Using the bounds $y_*\le 3$, (4.193)–(4.194), (4.200), and $\rho _-\omega _-\le \frac{\rho _0}{3}$ (Lemma 4.14), we obtain from (4.199)

4.201 $$\begin{aligned}&\frac{1}{2}\partial \omega _-^2(z) + \int _0^z \left( \frac{3}{\tau }+\frac{2\tau y_*^2 \omega _-^2}{1- y_*^2\tau ^2 \omega _-^2 }\right) \partial \omega _-^2 \,d\tau \nonumber \\&\le C \int _0^z \rho _0\tau \partial \omega _-^2\,d\tau + C \rho _0 \int _0^z \tau |\partial \rho _-| |\partial \omega _-| \, d\tau , \ \ z\le (6\rho _0)^{-\frac{1}{2}} \end{aligned}$$

Let $Z = \eta \rho _0^{-\frac{3}{4}}$ with a sufficiently small $\eta >0$ to be specified later. Note that $Z< (6\rho _0)^{-\frac{1}{2}}$ for all $\rho _0\ge 1$ and $\eta$ chosen sufficiently small and independent of $\rho _0$. For any $\tau \in [0,Z]$ we have $\rho _0 \le \eta ^{\frac{4}{3}} \tau ^{-\frac{4}{3}}$. Therefore $\rho _0 \tau \le \eta ^{\frac{4}{3}} \tau ^{-\frac{1}{3}}$. From these estimates and (4.201) we conclude

4.202 $$\begin{aligned}&\frac{1}{2}\partial \omega _-^2(z) + \int _0^z \left( \frac{3}{\tau }+\frac{2\tau y_*^2 \omega _-^2}{1- y_*^2\tau ^2 \omega _-^2 }\right) \partial \omega _-^2 \,d\tau \nonumber \\&\le C \int _0^z \eta ^{\frac{4}{3}} \tau ^{-\frac{1}{3}}\partial \omega _-^2\,d\tau + \frac{C}{\sqrt{3}} \rho _0 \left( \int _0^z\frac{3}{\tau }\partial \omega _-^2\,d\tau \right) ^{\frac{1}{2}} \left( \int _0^z \tau ^3 \partial \rho _-^2 \, d\tau \right) ^{\frac{1}{2}} \nonumber \\&\le C \int _0^z \eta ^{\frac{4}{3}} \tau ^{-\frac{1}{3}}\partial \omega _-^2\,d\tau + \frac{1}{2} \int _0^z\frac{3}{\tau }\partial \omega _-^2\,d\tau + \frac{C^2}{6} \rho _0^2 \Vert \partial \rho _-\Vert _{\infty }^2 \int _0^z \tau ^3\,d\tau , \ \ z\in [0,Z]. \end{aligned}$$

With $\eta$ chosen sufficiently small, but independent of $\rho _0$, we can absorb the first two integrals on the right-most side into the term $\int _0^z \frac{3}{\tau }\partial \omega _-^2\,d\tau$ on the left-hand side. Since $\int _0^z\tau ^3\,d\tau = \frac{1}{4} \eta ^4\rho _0^{-3}$ we conclude

4.203 $$\begin{aligned} \left| \partial \omega _-(z)\right| \le C\eta ^2 \rho _0^{-\frac{1}{2}} \Vert \partial \rho _-\Vert _{\infty }, \ \ z\in [0,Z]. \end{aligned}$$

We now integrate (4.197) and conclude from (4.198)

$$\begin{aligned} \left| \partial \rho _-(z) - 1\right|&\le C\rho _0 \Vert \partial \rho _-\Vert _{\infty } \int _0^z \tau \,d\tau + C\Vert \partial \omega _-\Vert _{\infty } \int _0^z \left( \rho _0^2 \tau + \rho _0 \tau + \rho _0^{2}\tau ^3\right) \,d\tau \nonumber \\&\le C\eta ^2 \Vert \partial \rho _-\Vert _{\infty }, \ \ z\in [0,Z], \end{aligned}$$

where we have used (4.203), (4.184), and $0\le z\le \eta \rho _0^{-\frac{3}{4}}$. Therefore,

$$\begin{aligned} \Vert \partial \rho _-\Vert _{\infty } \le 1 + C\eta ^2 \Vert \partial \rho _-\Vert _{\infty } \end{aligned}$$

and thus, for $\eta$ sufficiently small so that $C\eta ^2<\frac{1}{3}$, we have $\Vert \partial \rho _-\Vert _\infty \le \frac{3}{2}$. From here we infer

$$\begin{aligned} \partial \rho _-(z) \ge 1 - \frac{3}{2}C\eta ^2>\frac{1}{2}>0, \ \ z\in [0,Z]. \end{aligned}$$

$$\square $$

### Existence of the LP-solution connecting the origin to the sonic point

The goal of this section is to carry out the intersection argument to show that $\lim _{z\rightarrow 0^+}\omega (z;{\bar{y}}_*)=\frac{1}{3}$. Before that we prove an important technical lemma that will be used later on.

#### Lemma 4.19

Let $x_*\in {\mathcal {X}}$ (see (4.166) for the definition of ${\mathcal {X}}$) and assume that $s(x_*)=0$. Then

- $$\begin{aligned} \rho (z;x_*)>\omega (z;x_*), \ \ z\in (0,1); \end{aligned}$$
- $$\begin{aligned} \limsup _{z\rightarrow 0} z \omega (z;x_*) >0. \end{aligned}$$

#### Proof

*Proof of Part (a).* If not let

$$\begin{aligned} z_c : = \sup _{z\in (0,1)} \left\{ \rho (\tau ;x_*)-\omega (\tau ;x_*)>0, \ \tau \in (z,1) , \ \ \rho (z;x_*)=\omega (z;x_*)\ \right\} >0. \end{aligned}$$

At $z_c$ we have from (1.23)–(1.24) $\omega '(z_c;x_*) = \frac{1-3\omega }{z_c}<0$ and $\rho '(z_c;x_*)=0$. Therefore there exists a neighbourhood strictly to the right of $z_c$ such that $\omega '<0$, $\rho <\omega$, and $\rho '>0$. It is easily checkes that this property is dynamically trapped and we conclude

4.204 $$\begin{aligned} \omega '(z;x_*) \le \frac{1-3\omega (z;x_*)}{z} , \ \ z\le z_c. \end{aligned}$$

Integrating the above equation over $[z,z_c]$ we conclude

$$\begin{aligned} \omega (z;x_*) z^3 \ge \omega (z_c;x_*) z_c^3 - \frac{1}{3} z_c^3 = \left( \omega (z_c;x_*)-\frac{1}{3}\right) z_c^3 =:c>0. \end{aligned}$$

In other words $\omega (z;x_*)z\ge \frac{c}{z^2}\gg 1$ for sufficiently small *z*, which implies $s(x_*)>0$. A contradiction.

*Proof of Part (b).* By way of contradiction we assume that $\lim _{z\rightarrow 0}z\omega (z;x_*)=0$. For any $\epsilon >0$ choose $\delta >0$ so small that

$$\begin{aligned} 1- x_*^2 z^2 \omega (z;x_*)^2>1-\epsilon ^2, \ \ \text { i. e. }z x_*\omega (z;x_*)<\epsilon , \ \ z\in (0,\delta ). \end{aligned}$$

From (1.24) and (4.127) we then conclude

4.205 $$\begin{aligned} \omega '&\le \frac{1-3\omega }{z} + \frac{2 \epsilon x_*\omega }{1-\epsilon ^2} \frac{1}{x_*z} = \frac{1-\left( 3-C_*\epsilon \right) \omega }{z}, \end{aligned}$$

where $C_*=\frac{2}{1-\epsilon ^2}$. Letting $i_*:=\inf _{z\in (0,1]}\omega (z;x_*)>\frac{1}{3}$, we choose $\epsilon >0$ so small that

$$\begin{aligned} 1-\left( 3-C_*\epsilon \right) \omega (z;x_*)< 1-\left( 3-C_*\epsilon \right) i_*< - c_*<0, \ \ c_*: = -\frac{1-3i_*}{2}. \end{aligned}$$

From (4.204)

4.206 $$\begin{aligned} \omega ' \le -\frac{c_*}{z}, \ \ z\in (0,\delta ). \end{aligned}$$

Therefore

$$\begin{aligned} \omega (z;x_*) = \omega (\delta ;x_*) - \int _{z}^\delta \omega '(\tau ;x_*)\,d\tau \ge \omega (\delta ;x_*) +c_*\log \frac{\delta }{z} \longrightarrow _{z\rightarrow 0} \infty . \end{aligned}$$

As a consequence of (4.205), for sufficiently small $\epsilon$ and $z\ll 1$ we have

$$\begin{aligned} \omega ' \le -\frac{2\omega }{z}, \end{aligned}$$

which in turn implies $\omega (z;x_*)\ge C z^{-2}$ for sufficiently small *z*. Therefore, since $\omega (z;x_*)z >1$ for sufficiently small *z* we conclude $s({\bar{x}}_*)>0$, a contradiction to the assumption $s(x_*)=0$. $\quad \square$

We now recall Definition 1.4, where the notion of an upper and a lower solution is introduced. The next lemma shows that we can find a lower solution at a point $0<z_0\ll 1$ arbitrarily close to $z=0$.

#### Lemma 4.20

(Existence of a lower solution). There exists an $$\eta >0$$ such that for any $$z_0<\eta $$ there exists an $$y_{**}\in [{\bar{y}}_*,3]$$ such that $$(\rho (\cdot ;y_{**}),\omega (\cdot ;y_{**}))$$ is a lower solution at $$z_0$$. Moreover, there exists a universal constant *C* such that $$\rho _1<\frac{C}{z_0}$$, where $$\rho _-(z_0;\rho _1)=\rho (z_0;y_{**})$$.

#### Proof

For any $y_*\in Y$ we consider the function

4.207 $$\begin{aligned} F(y_*) : = \sup _{{{\tilde{y}}}_*\in [{\bar{y}}_*, y_*]} \left\{ z_{\frac{1}{3}}({{\tilde{y}}}_*)\right\} . \end{aligned}$$

The function $$y_*\mapsto F(y_*)$$ is clearly increasing, continuous, and by Lemma 4.13$$\lim _{y_*\rightarrow {\bar{y}}_*}F(y_*)=0$$. Therefore, the range of *F* is of the form $$[0,\eta ]$$ for some $$\eta >0$$. For any $$y_*\in Y$$, by Lemma 4.13, the supremum in (4.207) is attained, i.e. there exists $$y_{**}\in [{\bar{y}}_*, y_*]$$ such that $$F(y_*) = z_{\frac{1}{3}}(y_{**})=:z_0$$. Therefore, for any $${\bar{y}}_*< y_*< y_{**}$$ we have

$$\begin{aligned} s(y_*)<z_{\frac{1}{3}}(y_*)\le z_0. \end{aligned}$$

Due to  (4.173) we have the bound $$\rho (z_0;y_{**})>\omega (z_0;y_{**})=\frac{1}{3}$$. By Lemma 4.16 choosing $$\rho _0 = \rho _0(z_0) = \frac{\sqrt{3}}{\sqrt{2} y_{**} z_0}>1$$ we have

$$\begin{aligned} \rho _-(z_0;\rho _0)>\frac{1}{y_{**}z_0}>\rho (z_0;y_{**}), \end{aligned}$$

where we have used Lemma 4.2 in the last bound. On the other hand $$\rho _-(z_0;\frac{1}{3})=\frac{1}{3}<\rho (z_0;y_{**})$$ (where we recall that $$\rho _-(\cdot ;\frac{1}{3})$$ is the Friedman solution, see Remark 1.3). Using Remark 4.17 and the Intermediate Value Theorem, there exists a $$\rho _1\in (\frac{1}{3},\rho _0)$$ such that

$$\begin{aligned} \rho (z_0;y_{**}) = \rho _-(z_0;\rho _1). \end{aligned}$$

By (4.182) $$\omega _-(z_0;\rho _1)>\frac{1}{3} = \omega (z_0;y_{**})$$ and therefore $$(\rho (\cdot ,y_{**}),\omega (\cdot ;y_{**}))$$ is a lower solution at $$z_0$$. The upper bound on $$\rho _1$$ follows from our choice of $$\rho _0$$. $$\quad \square $$

The most delicate argument in this section is the following lemma, which states that $(\rho (\cdot ;y_*), \omega (\cdot ;y_*))$ is an upper solution at some $z_0\ll 1$ if $\lim _{z\rightarrow 0}\omega (z;y_*)\ne \frac{1}{3}$.

#### Lemma 4.21

If

$$\begin{aligned} \lim _{z\rightarrow 0} \omega (z;{\bar{y}}_*) \ne \frac{1}{3}, \end{aligned}$$

then there exists a universal constant *C* and an arbitrarily small $z_0>0$ such that $(\rho (\cdot ;{\bar{y}}_*),\omega (\cdot ;{\bar{y}}_*))$ is an upper solution at $z_0$ and $\rho _1<\frac{C}{z_0}$, where $\rho _-(z_0;\rho _1)=\rho (z_0;{\bar{y}}_*)$.

#### Proof

It is clear that $\liminf _{z\rightarrow 0}\omega (z;{\bar{y}}_*)\ge \frac{1}{3}$ as otherwise we would have ${\bar{y}}_*\in Y$, a contradiction to the definition (4.170) of ${\bar{y}}_*$ and the openness of *Y*. We distinguish three cases.

*Case 1.*

$$\begin{aligned} \liminf _{z\rightarrow 0} \omega (z;{\bar{y}}_*) >\frac{1}{3}. \end{aligned}$$

In this case ${\bar{y}}_*\in {\mathcal {X}}$ and by Proposition 4.12 we have $s({\bar{y}}_*)=0$. By part (b) of Lemma 4.19 there exists a constant $C>0$ and a sequence $\{z_n\}_{n\in {\mathbb {N}}}\subset (0,1)$ such that $\lim _{n\rightarrow \infty }z_n=0$ and

4.208 $$\begin{aligned} \omega (z_n;{\bar{y}}_*)>\frac{C}{{\bar{y}}_*z_n}. \end{aligned}$$

For any such $z_n$ we have by part (a) of Lemma 4.19 and Lemma 4.2

4.209 $$\begin{aligned} \rho (z_n;{\bar{y}}_*)>\omega (z_n;{\bar{y}}_*)>\frac{C}{{\bar{y}}_*z_n} > C \rho (z_n; {\bar{y}}_*). \end{aligned}$$

For any $0<z_n\ll 1$ sufficiently small consider $(\rho _-(\cdot ;\rho ^n_0),\omega _-(\cdot ;\rho ^n_0))$ with $\rho ^n_0=\rho _0(z_n)= \frac{\sqrt{3} }{\sqrt{2} {\bar{y}}_*z_n}>1$. By Lemmas 4.16 and 4.19

$$\begin{aligned} \rho _-(z_n;\rho ^n_0)> \frac{1}{{\bar{y}}_*z_n}>\rho (z_n;{\bar{y}}_*)>\omega (z_n;{\bar{y}}_*)>\frac{1}{3}. \end{aligned}$$

On the other hand,

$$\begin{aligned} \rho (z_n;{\bar{y}}_*)>\frac{1}{3} = \rho _-(z_n;\frac{1}{3}), \end{aligned}$$

where we recall that $\rho _-(\cdot ;\frac{1}{3})\equiv \frac{1}{3}$ is the Friedman solution. Moreover, by Remark 4.17$[0,z_n]\subset [0,s_-({{\tilde{\rho }}}_0))$ for all ${{\tilde{\rho }}}_0\subset [\frac{1}{3},\rho ^n_0]$. By the continuity of the map $[\frac{1}{3},\rho ^n_0]\ni {{\tilde{\rho }}}_0\mapsto \rho _-(z_n;{{\tilde{\rho }}}_0)$ the Intermediate Value Theorem implies that there exists $\rho ^n_1\in (\frac{1}{3},\rho ^n_0)$ such that

4.210 $$\begin{aligned} \rho _-(z_n;\rho _1^n) = \rho (z_n;{\bar{y}}_*) \ \ \hbox { for all sufficiently large}\ n\in {\mathbb {N}}. \end{aligned}$$

Let

$$\begin{aligned} c^n_1: = {\left\{ \begin{array}{ll} \exp \left( - \left( \rho ^n_1\right) ^{-1}\right) , &{} \text { if }\ \rho ^n_1> 1; \\ \exp \left( -1 \right) , &{} \text { if }\ \frac{1}{3} <\rho ^n_1 \le 1. \end{array}\right. } \end{aligned}$$

Clearly $c_1^n\ge e^{-1}=:c_1$ for all $n\in {\mathbb {N}}$. By Lemma 4.16$\rho _-(z_n;\rho ^n_1)\ge c_1\rho ^n_1$. Since $\omega _-(z_n;\rho ^n_1)< \left( \frac{\rho ^n_1}{3}\right) ^{\frac{1}{2}}$, we conclude together with (4.209) and (4.210) that for all *n* sufficiently large

4.211 $$\begin{aligned} \omega _-(z_n;\rho ^n_1)<\frac{1}{\sqrt{3c_1}}\rho _-(z_n;\rho ^n_1)^{\frac{1}{2}} = \frac{1}{\sqrt{3c_1}}\rho (z_n;{\bar{y}}_*)^{\frac{1}{2}} \le \frac{1}{\sqrt{3c_1 C}} \omega (z_n;{\bar{y}}_*)^{\frac{1}{2}}.\nonumber \\ \end{aligned}$$

By (4.209) $\omega (z_n;{\bar{y}}_*)$ grows to positive infinity as $z_n$ approaches zero. Therefore, we may choose a sufficiently large $N\in {\mathbb {N}}$ and set $z_0=z_N\ll 1$, $\rho _0=\rho ^N_0$, $\rho _1=\rho _1^N$ so that $\frac{1}{\sqrt{3c_1 C}} \omega (z_0;{\bar{y}}_*)^{\frac{1}{2}}<\omega (z_0;{\bar{y}}_*)$. Together with (4.211) this gives

$$\begin{aligned} \omega _-(z_0;\rho _1) < \omega (z_0;{\bar{y}}_*). \end{aligned}$$

We conclude that $(\rho (\cdot ;{\bar{y}}_*), \omega (\cdot ;{\bar{y}}_*))$ is an upper solution (see Definition 1.4) at $z_0$ and the upper bound on $\rho _1$ follows from our choice of $\rho _0$.

*Case 2.*

4.212 $$\begin{aligned} \frac{1}{3}<\limsup _{z\rightarrow 0} \omega (z;{\bar{y}}_*) <\infty , \ \ \liminf _{z\rightarrow 0}\omega (z;{\bar{y}}_*)=\frac{1}{3}. \end{aligned}$$

In particular ${\bar{y}}_*\in {\mathcal {Z}}$ (see (4.168)) and by Lemma 4.9$\rho (z;{\bar{y}}_*)>\omega (z;{\bar{y}}_*)$. Assumption (4.212) also implies that there exists a constant $c>0$ independent of *z* such that

4.213 $$\begin{aligned} \omega (z;{\bar{y}}_*)<c, \ \ z\in (0,1]. \end{aligned}$$

From (1.23) and the bound (4.127) we conclude

$$\begin{aligned} \rho '(z;{\bar{y}}_*) \ge - C \rho (z;{\bar{y}}_*), \end{aligned}$$

or equivalently $\left( \rho e^{Cz}\right) '\ge 0$; here $C>0$. This implies the boundedness of $\rho (\cdot ;{\bar{y}}_*)$, i.e.

4.214 $$\begin{aligned} \rho (z;{\bar{y}}_*)<c, \ \ z\in (0,1], \end{aligned}$$

where we have (possibly) enlarged *c* so that (4.213) and (4.214) are both true. There exists an $\eta >0$ and a sequence $\left\{ z_n\right\} _{n\in {\mathbb {N}}}$ such that $\lim _{n\rightarrow \infty }z_n=0$ and

$$\begin{aligned} \frac{1}{3} + \eta < \omega (z_n;{\bar{y}}_*), \ \ \text { and } \ \ \lim _{n\rightarrow \infty } \omega (z_n;{\bar{y}}_*) = \limsup _{z\rightarrow 0} \omega (z;{\bar{y}}_*). \end{aligned}$$

Since $\{ \rho (z_n;{\bar{y}}_*)\}_{n\in {\mathbb {N}}}$ is bounded, by Lemma 4.16 we can choose a $\rho _0>1$ such that $\rho _-(z_n;\rho _0) > \rho (z_n;{\bar{y}}_*)$ for all $n\in {\mathbb {N}}$. On the other hand $\rho (z_n;{\bar{y}}_*)>\frac{1}{3}=\rho _-(z_n;\frac{1}{3})$. By the intermediate value theorem there exists a sequence $\{\rho _0^n\}_{n\in {\mathbb {N}}}\subset (\frac{1}{3},\rho _0)$ such that

$$\begin{aligned} \rho _-(z_n;\rho _0^n) = \rho (z_n;{\bar{y}}_*). \end{aligned}$$

Since $\omega _-(z;\rho _0^n)^2\le \frac{\rho _0^n}{3}<\frac{\rho _0}{3}$ and $\rho _-(z;\rho _0^n)<\rho _0^n<\rho _0$ (Lemma 4.14) we conclude from (1.23)–(1.24) and Theorem 2.12 that $\left| \rho _-'(z_n;\rho _0^n) \right|$ and $\left| \omega _-'(z_n;\rho _0^n)\right|$ are bounded uniformly-in-*n*, by some constant, say *C*. Therefore

$$\begin{aligned} \omega _-(z_n;\rho _0^n) \le \frac{1}{3} + C z_n. \end{aligned}$$

We thus conclude that for a fixed *n* sufficiently large $\omega _-(z_n;\rho _0^n)<\frac{1}{3} + \eta <\omega (z_n;{\bar{y}}_*)$. Therefore, $\omega (\cdot ;{\bar{y}}_*)$ is an upper solution (see Definition 1.4) at $z_0:=z_n$ with $\rho _1=\rho _0^n$. The claimed upper bound on $\rho _1$ is clear.

*Case 3.*

$$\begin{aligned} \frac{1}{3}<\limsup _{z\rightarrow 0} \omega (z;{\bar{y}}_*) =\infty , \ \ \liminf _{z\rightarrow 0}\omega (z;{\bar{y}}_*)=\frac{1}{3}. \end{aligned}$$

As $\omega (\cdot ;{\bar{y}}_*)$ must oscillate between $\frac{1}{3}$ and $\infty$ we can use the mean value theorem to conclude that there exists a sequence $\left\{ z_n\right\} _{n\in {\mathbb {N}}}$ such that $\lim _{n\rightarrow \infty }z_n=0$ and

4.215 $$\begin{aligned} \omega (z_n;{\bar{y}}_*)>n, \ \ \text { and } \ \ \omega '(z_n;{\bar{y}}_*)=0. \end{aligned}$$

We claim that there exist $N_0>0$ and $0<\eta \ll 1$ such that

4.216 $$\begin{aligned} \omega (z_n;{\bar{y}}_*)\ge \frac{r-\eta }{{\bar{y}}_*z_n}, \ \ n\ge N_0, \end{aligned}$$

where $r<1$ is the positive root of the quadratic polynomial $3x^2+2x-3$. To prove this, assume that (4.216) is not true. Then there exists a subsequence of $\{z_n\}_{n\in {\mathbb {N}}}$ such that $\omega (z_n;{\bar{y}}_*)< \frac{r-\eta }{{\bar{y}}_*z_n}$ and therefore

$$\begin{aligned} \frac{2{\bar{y}}_*z_n\omega (z_n;{\bar{y}}_*)}{1 - ({\bar{y}}_*z_n\omega (z_n;{\bar{y}}_*))^2} < 3 - C(\eta ), \end{aligned}$$

for some $C(\eta )>0$. Since $\rho -\omega <\frac{1}{{\bar{y}}_*z}$ by (4.127) we have

$$\begin{aligned} \omega '(z_n;{\bar{y}}_*)&<\frac{1-3\omega (z_n;{\bar{y}}_*)}{z_n} + \frac{2{\bar{y}}_*z_n\omega (z_n;{\bar{y}}_*)}{1 - ({\bar{y}}_*z_n\omega (z_n;{\bar{y}}_*))^2}\frac{\omega (z_n; {\bar{y}}_*)}{z_n} \\&= \frac{\omega (z_n;{\bar{y}}_*)}{z_n}\left( \frac{1}{\omega (z_n; {\bar{y}}_*)}-3 + \frac{2{\bar{y}}_*z_n\omega (z_n;{\bar{y}}_*)}{1 - ({\bar{y}}_*z_n\omega (z_n;{\bar{y}}_*))^2} \right) \\&< (\frac{1}{\omega (z_n;{\bar{y}}_*)} -C(\eta )) \frac{\omega (z;{\bar{y}}_*)}{z} <0 \ \ \text { for }n \text { sufficiently large}. \end{aligned}$$

This is a contradiction to (4.215). We can therefore repeat the same argument following (4.208) to conclude that $\omega (\cdot ;{\bar{y}}_*)$ is an upper solution at $z_0:=z_n$, for some *n* sufficiently large. The upper bound on $\rho _1$ follows in the same way. $\quad \square$

We now use a continuity argument to show the following key proposition.

#### Proposition 4.22

The limit $\lim _{z\rightarrow 0}\omega (z;{\bar{y}}_*)$ exists and

$$\begin{aligned} \lim _{z\rightarrow 0}\omega (z;{\bar{y}}_*) = \frac{1}{3}. \end{aligned}$$

#### Proof

Assume that the claim is not true. By Lemmas 4.20 and 4.21 we can find a $$0<z_0\ll 1$$ and $$y_{**}\in Y$$ so that $$(\rho (\cdot ;y_{**}),\omega (\cdot ;y_{**}))$$ and $$(\rho (\cdot ;{\bar{y}}_*),\omega (\cdot ;{\bar{y}}_*))$$ are respectively a lower and an upper solution at $$z_0$$. Without loss of generality let

$$\begin{aligned} A: =\rho (z_0;y_{**})< \rho (z_0;{\bar{y}}_*)=:B. \end{aligned}$$

By Lemmas 4.20 and 4.21 there exist $\rho _A,\rho _B>\frac{1}{3}$ such that $A=\rho _-(z_0;\rho _A)$, $B=\rho _-(z_0;\rho _B)$, and $\rho _A,\rho _B\in (\frac{1}{3},\rho _0)$, where $\rho _0\gg 1$ and $z_0 \le C\frac{1}{\rho _0}$. Therefore by Lemma 4.18, $\partial _{\rho _0}\rho _-(z_0;{{\tilde{\rho }}}_0)>0$ for all ${{\tilde{\rho }}}_0\in [\frac{1}{3},\rho _0]$, since $\rho _0^{-\frac{3}{4}}\gg \rho _0^{-1}$ for $\rho _0$ large. By the inverse function theorem, there exists a continuous function $\tau \mapsto f(\tau )$ such that

$$\begin{aligned} \rho _-(z_0;f(\tau ))&= \tau , \ \ \tau \in [A,B] \\ f(\rho _A)&= A. \end{aligned}$$

By strict monotonicity of ${{\tilde{\rho }}}_0\mapsto \rho _-(z_0;{{\tilde{\rho }}}_0)$ on $(0,\rho _0]$ the inverse *f* is in fact injective and therefore $f(\rho _B)=B$. We consider the map

$$\begin{aligned}{}[{\bar{y}}_*,y_{**}] \ni y_*\mapsto \omega (z_0;y_*) - \omega _-(z_0;f(\rho (z_0;y_*))) = : h(y_*). \end{aligned}$$

By the above discussion *h* is continuous, $$h(A)<0$$ and $$h(B)>0$$. Therefore, by the Intermediate Value Theorem there exists a $$y_s\in ({\bar{y}}_*,y_{**})$$ such that $$h(y_s)=0$$. The solution $$(\rho (\cdot ;y_s),\omega (\cdot ;y_s))$$ exists on [0, 1], satisfies $$\omega (0)=\frac{1}{3}$$ and belongs to *Y*. This is a contradiction to (4.174). $$\quad \square $$

It remains to show that the solution is regular at $z=0$ and we do this by showing that it coincides with a solution $(\rho _-(\cdot ;\rho _*),\omega _-(\cdot ;\rho _*))$ emanating from the origin, with the correct choice of $\rho _*$.

#### Proposition 4.23

There exists a constant $C_*>0$ so that

$$\begin{aligned} \left| \rho (z;{\bar{y}}_*)\right| + \left| \omega (z;{\bar{y}}_*)\right| + \left| \frac{\omega (z;{\bar{y}}_*)-\frac{1}{3}}{z^2}\right| \le C_*, \ \ z\in (0,1]. \end{aligned}$$

The solution $\rho (\cdot ;{\bar{y}}_*):(0,1]\rightarrow {\mathbb {R}}_{>0}$ extends continuously to $z=0$ and $\rho _*:=\rho (0;{\bar{y}}_*)<\infty$. Moreover, the solution $(\rho (\cdot ;{\bar{y}}_*),\omega (\cdot ;{\bar{y}}_*))$ coincides with $(\rho _-(\cdot ;\rho _*), \omega _-(\cdot ;\rho _*))$ and it is therefore analytic at $z=0$ by Theorem 2.12.

#### Proof

By Proposition 4.22 it is clear that $\omega (\cdot ;{\bar{y}}_*)$ is bounded on [0, 1]. From (1.23) and (4.127) we conclude $|\rho '| \le C\rho$ and thus $\rho$ is bounded up to $z=0$. Using the boundedness of $\rho$ and $\omega$, equation (1.23) immediately implies $|\rho '(z)|\lesssim z$ for all $z\in [0,1]$.

Since $\rho (\cdot ;{\bar{y}}_*)-\omega (\cdot ;{\bar{y}}_*)\ge 0$ on (0, 1] and both $\rho (\cdot ;{\bar{y}}_*)$ and $\omega (\cdot ;{\bar{y}}_*)$ are positive, we conclude from (1.23) that $\rho '\le 0$ and therefore the limit $\rho _*:=\lim _{z\rightarrow 0}\rho (z;{\bar{y}}_*)$ exists and by the above it is finite. Let

$$\begin{aligned} \zeta = \omega -\frac{1}{3}\ge 0. \end{aligned}$$

It is then easy to check from (1.24)

$$\begin{aligned} \left( \zeta z^3\right) ' = z^3 \frac{2 {\bar{y}}_*^2 z \omega ^2(\rho -\omega )}{1-{\bar{y}}_*^2 z^2\omega ^2} \end{aligned}$$

and therefore, since $\omega$ and $\rho$ are uniformly bounded for any $0<z_1<z$ we obtain

$$\begin{aligned} \zeta (z) z^3 - \zeta (z_1) z_1^3 \le C \int _{z_1}^{z}\tau ^4\,d\tau = \frac{C}{5}\left( z^5-z_1^5\right) . \end{aligned}$$

We now let $z_1\rightarrow 0^+$ and conclude

4.217 $$\begin{aligned} \zeta (z)\lesssim z^2 \end{aligned}$$

Consider now ${{\bar{\rho }}}(z):=\rho (z;{\bar{y}}_*)-\rho _-(z;\rho _*)$ and ${{\bar{\omega }}}(z):=\omega (z;{\bar{y}}_*)-\omega _-(z;\rho _*)$, both are defined in a (right) neighbourhood of $z=0$ and satisfy

$$\begin{aligned} {{\bar{\rho }}}'&= O(1){{\bar{\rho }}} + O(1){{\bar{\omega }}} \\ {{\bar{\omega }}}'&= - 3\frac{{{\bar{\omega }}}}{z} + O(1){{\bar{\rho }}} + O(1){{\bar{\omega }}}, \end{aligned}$$

where ${{\bar{\rho }}}(0)={{\bar{\omega }}}(0)=0$. Here we have used the already proven boundedness of $(\rho (\cdot ;{\bar{y}}_*),\omega (\cdot ;{\bar{y}}_*))$ and the boundedness of $(\rho (\cdot ;\rho _*),\omega (\cdot ;\rho _*))$, see Lemma 4.14. We multiply the first equation by ${{\bar{\rho }}}$, the second by ${{\bar{\omega }}}$, integrate over [0, *z*] and use Cauchy-Schwarz to get

4.218 $$\begin{aligned} {{\bar{\rho }}}(z)^2+{{\bar{\omega }}}(z)^2 + 3\int _0^z\frac{{{\bar{\omega }}}(\tau )^2}{\tau }\,d\tau \le C \int _0^z \left( {{\bar{\rho }}}(\tau )^2+{{\bar{\omega }}}(\tau )^2\right) \,d\tau . \end{aligned}$$

We note that $\int _0^z\frac{{{\bar{\omega }}}(\tau )^2}{\tau }\,d\tau$ is well-defined, since ${{\bar{\omega }}} = \zeta - \zeta _-$, where $\zeta _-=\omega _--\frac{1}{3}$; we use (4.217) and observe $\zeta _-\lesssim z^2$ in the vicinity of $z=0$ by the analyticity of $\omega _-$, see Theorem 2.12. Therefore ${{\bar{\rho }}}(z)^2+{{\bar{\omega }}}(z)^2=0$ by (4.218). The analyticity claim now follows from Theorem 2.12. $\quad \square$

## Proof of the Main Theorem

The existence of an LP-type solution, the corresponding boundary conditions stated in Theorem 1.2, and (1.18) follow directly from Propositions  3.3, 4.12, 4.23. The real analyticity locally around the sonic point and the origin follows from Theorems 2.10 and 2.12 respectively, while away from these two points it follows from the standard ODE-theory. Unwinding the change of variables (1.22) and (1.8) we easily obtain (1.19) from (2.122) when $z\ge 1$ or equivalently $y\ge {\bar{y}}_*$. To get (1.19) for $z<1$ we first rewrite (1.24) in the form

$$\begin{aligned} \omega ' = \frac{1-\omega }{z} + \frac{-2\omega +2{\bar{y}}_*^2z^2\omega ^2\rho }{z \left( 1-{\bar{y}}_*^2z^2\omega ^2\right) }. \end{aligned}$$

For any $z\in (0,1)$ we have $\frac{1}{{\bar{y}}_*^2z^2}\omega (z;{\bar{y}}_*)\rho (z;{\bar{y}}_*) <1$ by (4.127) and the bound $\omega <\rho$ which follows from (4.173) and ${\bar{y}}_*\in {\mathcal {Z}}$. This yields

5.219 $$\begin{aligned} \omega '<\frac{1-\omega }{z}, \ \ z\in (0,1). \end{aligned}$$

Let $z_c:=\sup \{z\in (0,1)\,\big | \omega (z;{\bar{y}}_*)<1\}$. We use a contradiction argument and assume $z_c<1$. Since $\omega (0;{\bar{y}}_*)=\frac{1}{3}$ it follows by continuity that $z_c>0$. At $z_c$ we must therefore have $\omega (z_c)=1$ and $\omega '(z_c)\ge 0$. On the other hand, from (5.219) we conclude

$$\begin{aligned} \omega '(z_c;{\bar{y}}_*)<\frac{1-\omega (z_c;{\bar{y}}_*)}{z_c} =0, \end{aligned}$$

a contradiction. We conclude that for any $z\in [0,1)$ we have $\omega (z)<1$. By Proposition 4.12 we also have $\omega (z,{\bar{y}}_*)\ge \frac{1}{3}$ and therefore (1.19) follows also in the region $z\in [0,1)$ or equivalently $y\in [0,{\bar{y}}_*)$.
